# Burden of metabolic syndrome in the global adult HIV-infected population: a systematic review and meta-analysis

**DOI:** 10.1186/s12889-024-20118-3

**Published:** 2024-09-28

**Authors:** Deondara Trachunthong, Mathuros Tipayamongkholgul, Suchintana Chumseng, Worrayot Darasawang, Kanitta Bundhamcharoen

**Affiliations:** 1 Department of Epidemiology, Faculty of Public Health, Mahidol University, Bangkok, Thailand; 2 ASEAN Institute for Health Development, Mahidol University, 999 Phuttamontol Sai 4, Salaya, Phuttamontol, Nakhon Pathom, 73170 Thailand; 3 Institute of HIV Research and Innovation (IHRI), Bangkok, Thailand; 4 Department of Social Medicine, Buriram Hospital, Buriram, Thailand; 5 International Health Policy Program (IHPP), Ministry of Public Health, Nonthaburi, Thailand

**Keywords:** Metabolic syndrome, Global adult HIV-infected population, People living with HIV/AIDS, HIV infection, Antiretroviral therapy

## Abstract

**Background:**

Metabolic syndrome (MetS) elevates the risk of heart disease and stroke. In recent decades, the escalating prevalence of MetS among people living with HIV/AIDS (PLWHA) has garnered global attention. Despite MetS development being associated with both traditional and HIV-related factors, evidence from prior studies has shown variability across geographical regions. This study aimed to conduct a systematic review and meta-analysis of MetS burdens in adult PLWHA at the regional and global levels, focusing on the common effect size of HIV infection and antiretroviral therapy (ART) on MetS.

**Methods:**

This review followed the PRISMA 2020 guidelines. A comprehensive search and review of original articles related to MetS and HIV published in peer-reviewed journals between January 2000 and December 2023 were conducted. A random effects model was used to calculate the pooled prevalence/incidence of MetS and the common effect size of HIV infection and ART exposure on MetS.

**Results:**

A total of 102 studies from five continents comprising 78,700 HIV-infected participants were included. The overall pooled prevalence of MetS was 25.3%, 25.6% for PLWHA on ART, and 18.5% for those not receiving treatment. The pooled incidence of MetS, calculated from five studies, was 9.19 per 100 person-years. The highest pooled prevalence of MetS was observed in the Americas (30.4%), followed by the Southeast Asia/Western Pacific regions (26.7%). HIV-infected individuals had 1.6 times greater odds of having MetS than non-HIV-infected individuals did (pooled OR = 1.604; 95% CI 1.154–2.230), and ART exposure had 1.5 times greater odds of having MetS than nontreatment had (pooled OR = 1.504; 95% CI 1.217–1.859).

**Conclusions:**

HIV infection and ART exposure contribute significantly to the increased burden of MetS. Regions with a high burden of HIV and MetS should prioritize awareness and integrated care plans for major noncommunicable diseases (NCDs), such as heart disease and stroke. The implementation of integrated care for HIV/AIDS patients and NCDs is essential for addressing the high burden of multimorbidity in PLWHA.

**Registration number:**

INPLASY202290018

**Supplementary Information:**

The online version contains supplementary material available at 10.1186/s12889-024-20118-3.

## Introduction

Currently, biological and epidemiological evidence indicates the risk of noncommunicable diseases (NCDs) due to preexisting infectious diseases [1]. Human immunodeficiency virus (HIV) infection is one example [2]. An increasing trend toward increasing NCD incidence among people living with HIV/AIDS (PLWHA) with prolonged longevity related to the coverage and effectiveness of antiretroviral therapy (ART) has been reported worldwide [3–5].

Over the past few decades, there has been a growing body of evidence indicating linkages between HIV and NCDs [6–8]. The evidence indicates hypothesis-determinant factors of an excess NCDs incidence among PLWHA are: (1) HIV causes persistent immune activation and chronic inflammation leading to an increased risk of metabolic complications and organ damage [9, 10]; (2) ART side effects such as metabolic complications may contribute to organ damage, however evidence of side effects lowered among newer generations of ART [11]; and (3) NCDs traditional risk factors including older age, smoking, excess consumption of alcohol, unhealthy diet and physical inactivity [12, 13]. These three hypothesis-determinant factors likely induce metabolic syndrome (MetS), which is a cluster of interrelated risk factors that include glucose intolerance, hypertension, dyslipidemia and obesity [14]. It is well documented that MetS is a strong predictor of type 2 diabetes, cardiovascular disease, renal disease and premature mortality [15–20]. The prevalence of MetS among PLWHA varies across regions worldwide and may be related to the diversity of social contexts, health systems, health-related behaviors, and disparities in ART management across these regions [21–24]. Therefore, epidemiologic evidence on the global and regional burdens of MetS is essential for identifying the most vulnerable regions and informing specific prevention priorities across regions.

The development of MetS in PLWHA is caused by the complex interplay of various hypothetical factors mentioned earlier, but the effects of HIV infection [25–27] and ART exposure [28–31] have not been fully elucidated. Therefore, it is necessary to assemble research findings from different continents worldwide to estimate the common effect size of HIV and ART exposure on MetS development. This information will be beneficial for identifying high-risk regions for MetS and planning the prevention and treatment of high-risk patients. In this study, we conducted a systematic review and meta-analysis to synthesize data on the burdens of MetS in the adult HIV population at the regional and global levels as well as to evaluate the common effect size of HIV infection and the use of ART.

## Methods

### Protocol and registration

A study protocol was developed and published prior to the conduction of this review. The protocol was registered with the International Platform of Registered Systematic Review and Meta-Analysis Protocols (INPLASY) (registration number INPLASY202290018). Metabolic syndrome was selected from four major noncommunicable diseases e.g., metabolic syndrome, diabetes, cardiovascular disease, and chronic kidney disease of the study protocol for analysis and presentation.

### Eligibility criteria

Since our systematic review and meta-analysis aims to identify overall effect of HIV related factors and conventional factors on MetS which designs of the observational studies can ethically evidence the issue. Thus, we included observational studies conducted among PLWHA aged 15 years and older during the era of highly active antiretroviral therapy (HAART) availability since 1996. We limited our search to peer-reviewed original articles published in English and Thai from January 2000 to December 2023. Studies reporting the prevalence/incidence of MetS and odds ratios/relative risk of HIV status and use of ART for MetS were included. For duplicated longitudinal published studies, the study with the longest follow-up period was selected. The full inclusion and exclusion criteria are presented in the aforementioned published protocol [32]. Currently, seven different definitions of MetS exist and are used in studies across the globe. All the definitions are shown in additional file 1.

### Source of information and search strategies

This review was guided and written according to the Preferred Reporting Items for Systematic Review and Meta-Analysis Protocols (PRISMA-P) 2020 statement [33]. We searched PubMed/MEDLINE, Scopus, Embase, EBSCO, Thai journals online (ThaiJO), Thai Digital Collection (TDC), Thai Journal Index (TJI), and Thai Journal citation index (TCI) using the keywords ‘HIV’, ‘ART’, and ‘metabolic syndrome’. The search strategy is shown in additional file 2. All the search terms used were translated into Thai for the ThaiJO, TDC, TJI and TCI databases. The Thai databases were chosen because the project will further estimate the MetS burden among Thai people living with HIV.

### Study selection and data extraction

Study eligibility and data extraction were independently performed by the DT and SC, and interrater agreement was assessed by Cohen’s kappa coefficient. Study selection disagreements were resolved by a third reviewer (WD). Discrepancies in the data extraction were resolved through discussion among the reviewers (DT and ST), and all the processes were approved by the senior investigator (MT). The following data were extracted: author, publication year, study period, study site, country of study origin, WHO region, study design, sample size, length of follow-up, age, proportion of sex, race, body mass index (BMI), comorbidity, HIV status, ART status, duration of HIV infection, duration of ART, CD4 cell count, viral load, prevalence, incidence, and number of MetS patients.

### Quality assessment

We used the Newcastle–Ottawa scale (N-O scale) to critically assess the quality of the observational studies [34] based on the study design. The instrument comprises three domains, i.e., participant selection, comparability and exposure/outcome assessment [35]. For cohort and case‒control studies, the N-O scale was categorized into three levels according to the number of stars in each of the three domains recommended by the Standards of the Agency for Healthcare Research and Quality (AHRQ). Good quality (> 3 stars in the selection domain, 1–2 stars in the comparability domain, 2–3 stars in the outcome/exposure domain); fair quality (2 stars in the selection domain and 1–2 stars in the comparability domain and 2–3 stars in the outcome/exposure domain); poor quality (fewer than one star in all domains) [35]. For cross-sectional studies, we used the adapted N-O scale developed by Herzog et al., which was divided into four categories: very good studies (9–10 points), good studies (7–8 points), satisfactory studies (5–6 points) and unsatisfactory studies (0 to 4 points) [36]. The quality assessment tools used are shown in additional file 3.

### Data synthesis and analysis

We meta-analyzed the global pooled prevalence and incidence of MetS in PLWHA regardless of the MetS definition and stratified by WHO region. Pooled estimates of odds ratios /risk ratios were calculated for two factors, i.e., HIV status and ART status. Before undertaking the meta-analysis, the effect sizes of all the selected studies were converted to one single effect measure with the same unit of measurement. All pooled estimates were calculated according to appropriate procedures and are presented in forest plots. The Cochrane Q test and I^2^ test were used to identify the proper technique for calculating the pooled estimate. A fixed-effects model using the inverse variance method was applied when the p value of the Cochrane Q test was ≥ 0.1 and I^2^ was < 25%, while a random-effects model with restricted maximum likelihood (REML) method was used when the p value of the Cochrane Q test was < 0.1 and I^2^ was ≥ 25%. Meta-regression and subgroup stratified analyses were performed to explore potential sources of heterogeneity among the studies. A sensitivity analysis was carried out to evaluate the robustness of the results of the individual studies and datasets. To measure any changes in the estimation, the analysis was repeated according to differences in WHO regions, MetS definition, age of study sample, and study quality. The existence of publication bias was assumed when the funnel plot showed asymmetrical spread of the dots or the p value of Egger’s test was < 0.05. The analyses of this study were conducted in STATA (version 15; STATA Corporation, College Station, Texas, USA). The report for systematic review and meta-analysis adhered to the PRISMA 2020 checklist, as shown in additional 4.

## Results

**Fig. 1:**
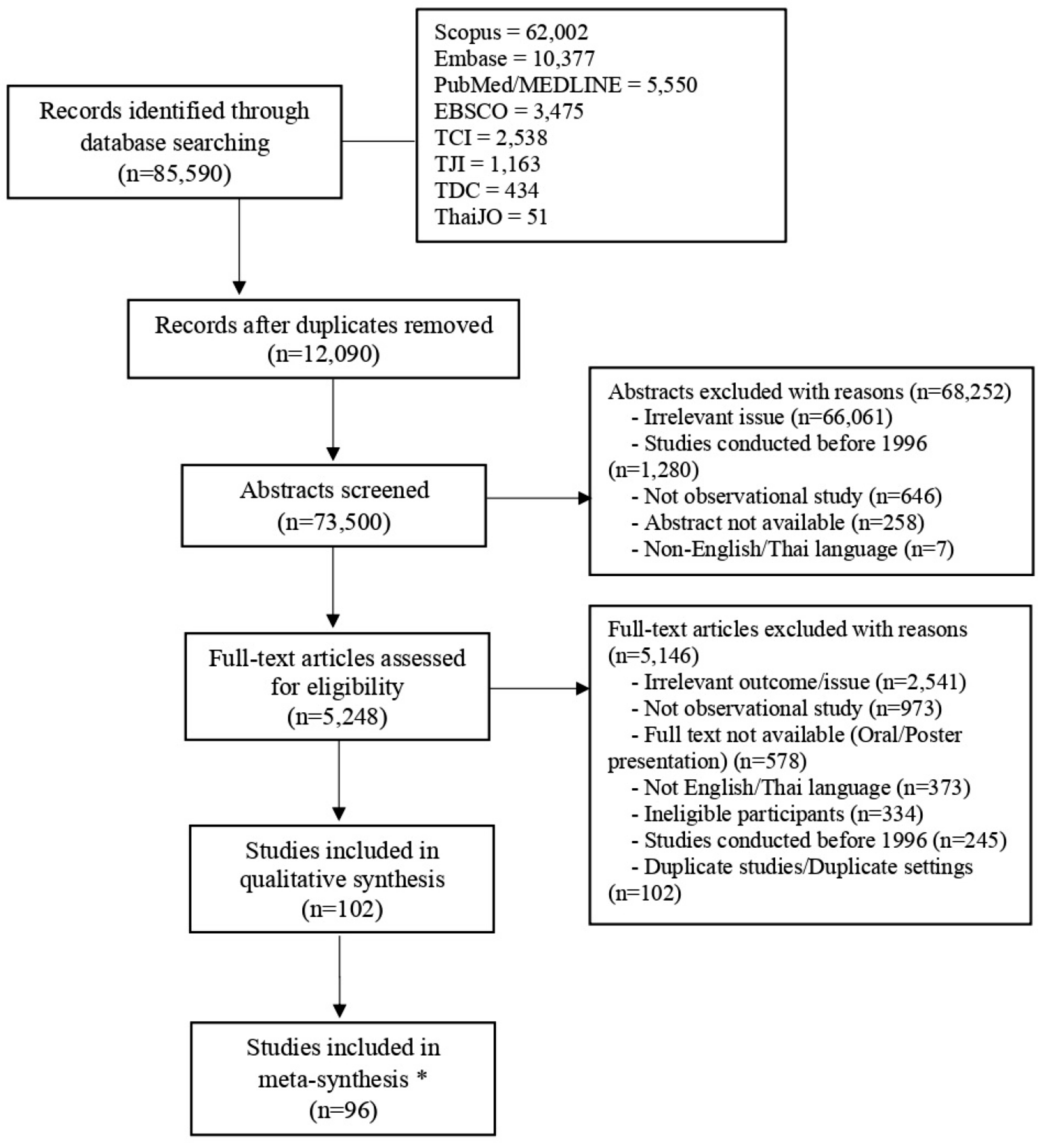
PRISMA flow diagram of study selection process

The process for selecting the relevant studies is summarized in Fig. 1. In total, 85,590 records were identified via database searches. After removing all the duplicates, we scanned the titles and abstracts of 73,500 articles, 5,248 of which were further reviewed via full texts. Of these, 102 articles met the inclusion criteria and were selected for this review. The authors’ performance agreement was tested by Cohen’s kappa statistic; the kappa statistic was 0.829 for abstract selection and 0.669 for full-text selection.

### Characteristics of included studies

The characteristics of the included studies are summarized in additional file 5. Of the 102 eligible studies, 34 were from the African region (AFR), 26 from the European region (EUR), 21 from the region of the Americas (AMR), 20 from Southeast Asia and the Western Pacific region (SEAR and WPR), and one from the international. Among the 102 articles, 87 were cross-sectional studies, 12 were cohort studies and 3 were case‒control studies, 59.8% of which were good quality. A total of 78,700 HIV-infected individuals were sampled; 65,639 were on ART, and 4,262 were not treated (the ART status of 8,799 patients was not reported). The patients’ mean/median age ranged from 20.3 to 54 years [37, 38], and their BMI was in the 19 to 31 kg/m^2^ range [39, 40]. The majority of HIV-infected samples contained a greater percentage of males than females; however, four studies included only females, and three studies included males. The mean/median duration of HIV infection ranged from 7.6 months to 21.8 years [41, 42], the mean/median duration of ART ranged from 13 months to 18.8 years [43, 44], and the mean/median CD4 + cell count was between 105.3 and 803 cells/µL [39, 45].

### Meta-analysis for pooled prevalence and incidence

**Fig. 2:**
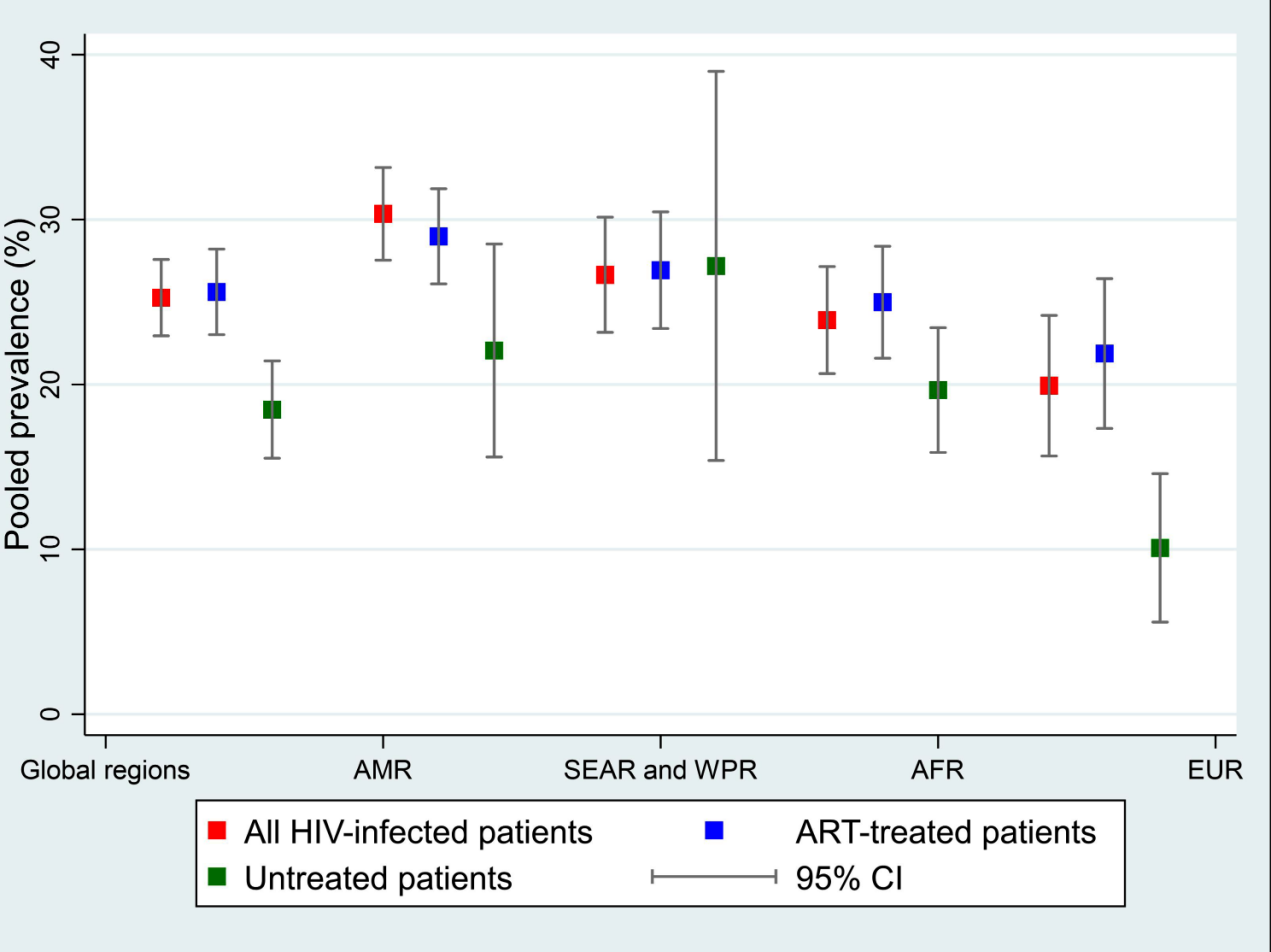
Pooled prevalence of Metabolic syndrome by regions. AMR: Region of the Americas; SEAR and WPR: South-East Asia and Western Pacific regions; AFR: African region; EUR: European region

The prevalence of MetS among the global HIV-infected population was reported in 92 studies and ranged from 5.2 to 52.8% [46, 47]. The pooled prevalence of MetS among HIV-infected patients was 25.26% (95% CI, 22.95–27.58; I^2^ = 97.9%). Patients receiving ART had a higher pooled prevalence of MetS than non-treatment patients 25.61% (95% CI, 23.02–28.21; I^2^ = 97.8%) vs. 18.48% (95% CI, 15.53–21.43; I^2^ = 85.3%), respectively. The highest pooled prevalence was found in AMR (pooled prevalence = 30.35%; 95% CI, 27.54–33.16; I^2^ = 91%) followed by SEAR and WPR (pooled prevalence = 26.65%; 95% CI, 23.16–30.15; I^2^ = 93.6%), AFR (pooled prevalence = 23.90%; 95% CI, 20.66–27.15; I^2^ = 94.7%); EUR (pooled prevalence = 19.93%; 95% CI, 15.66–24.19; I^2^ = 97.2%) (Fig. 2). One international study conducted in Europe, the United States, and Australia revealed that the prevalence of MetS among PLWHA under ART increased from 19.4% in 2000 to 41.6% in 2006–2007 [48]. Forest plots of the MetS prevalence by WHO regions for overall, ART and untreated PLWHA are shown in additional file 6. The pooled prevalence was not significantly different according to MetS definitions, age groups, and quality of studies; sensitivity analysis is shown in additional file 7. Only five studies from the AFR [49], AMR [28], EUR [50, 51], SEAR, and WPR [52] were included for estimating pooled incidence of MetS at approximately 9.19 per 100 person-years (95% CI, 3.24–15.15; I^2^ = 98.8%). Forest plot of the MetS incidence is shown in additional file 8.

### Meta-analysis for the effect of HIV infection

**Fig. 3:**
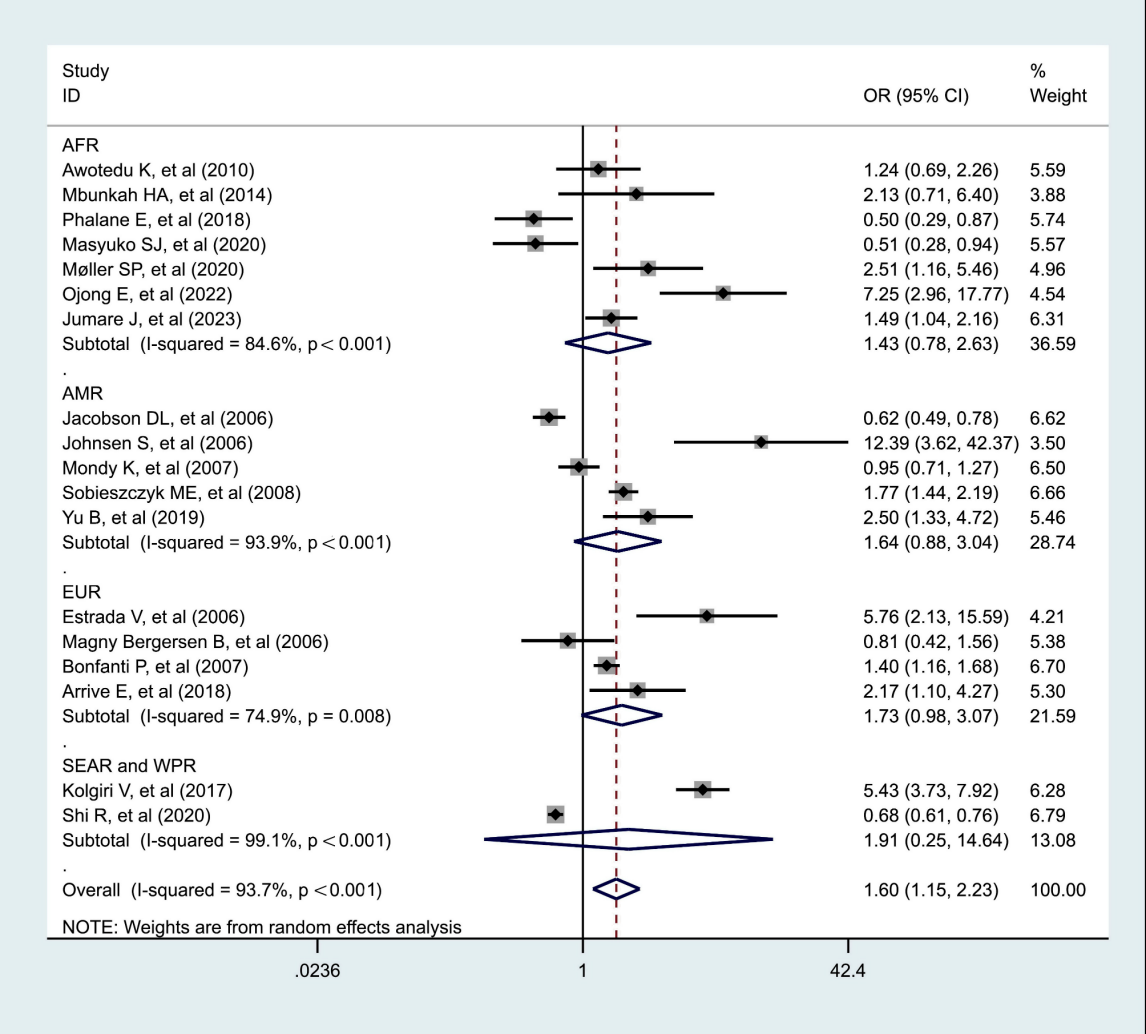
Forest plot showing the pooled OR of Metabolic syndrome associated with HIV infection by regions. AFR: African region; AMR: Region of the Americas; EUR: European region; SEAR and WPR: South-East Asia and Western Pacific regions. (For each study the black box represents the study estimate and the horizontal bar represent the 95% CI. The diamond at the lower tail is the pooled effect estimates from random effect model.)

Eighteen studies reported the odds of MetS comparing between HIV-infected and non-HIV infected people. The global pooled odds ratio for developing MetS in according with HIV infection was approximately 1.604 (95% CI, 1.154–2.230, *p* = 0.005; I^2^ = 93.7%). SEAR and WPR showed the highest pooled odds ratio 1.906 (95% CI, 0.248–14.638, *p* = 0.54 I^2^ = 99.1%), followed by EUR (pooled odds ratio = 1.733; 95% CI, 0.978–3.068, *p* = 0.059, I^2^ = 74.9%); AMR (pooled odds ratio = 1.637; 95% CI, 0.882–3.038, *p* = 0.118; I^2^ = 93.9%), and AFR (pooled odds ratio = 1.428; 95% CI, 0.776–2.628, *p* = 0.252; I^2^ = 84.6%) (Fig. 3). Pooled odds ratios for developing MetS due to HIV infection in the sensitivity analysis varied depending on age groups, and study quality. Sensitivity analysis revealed the pooled odds ratio among patients aged > 41 years was 1.111 (95% CI, 0.716–1.726, *p* = 0.638; I^2^ = 95.3) and those ≤ 41 years was 2.513(95% CI, 1.535–4.116, *p* < 0.001; I^2^ = 84.1%). The pooled odds ratios for developing MetS among good and fair studies were 1.248(95% CI, 0.920–1.692, *p* = 0.154; I^2^ = 92%), and 3.716(95% CI, 1.416–9.750, *p* = 0.008; I^2^ = 66.2%), respectively. Sensitivity analysis, meta-regression and subgroup analysis are shown in additional file 9.

### Meta-analysis for the effect of antiretroviral treatment

**Fig. 4:**
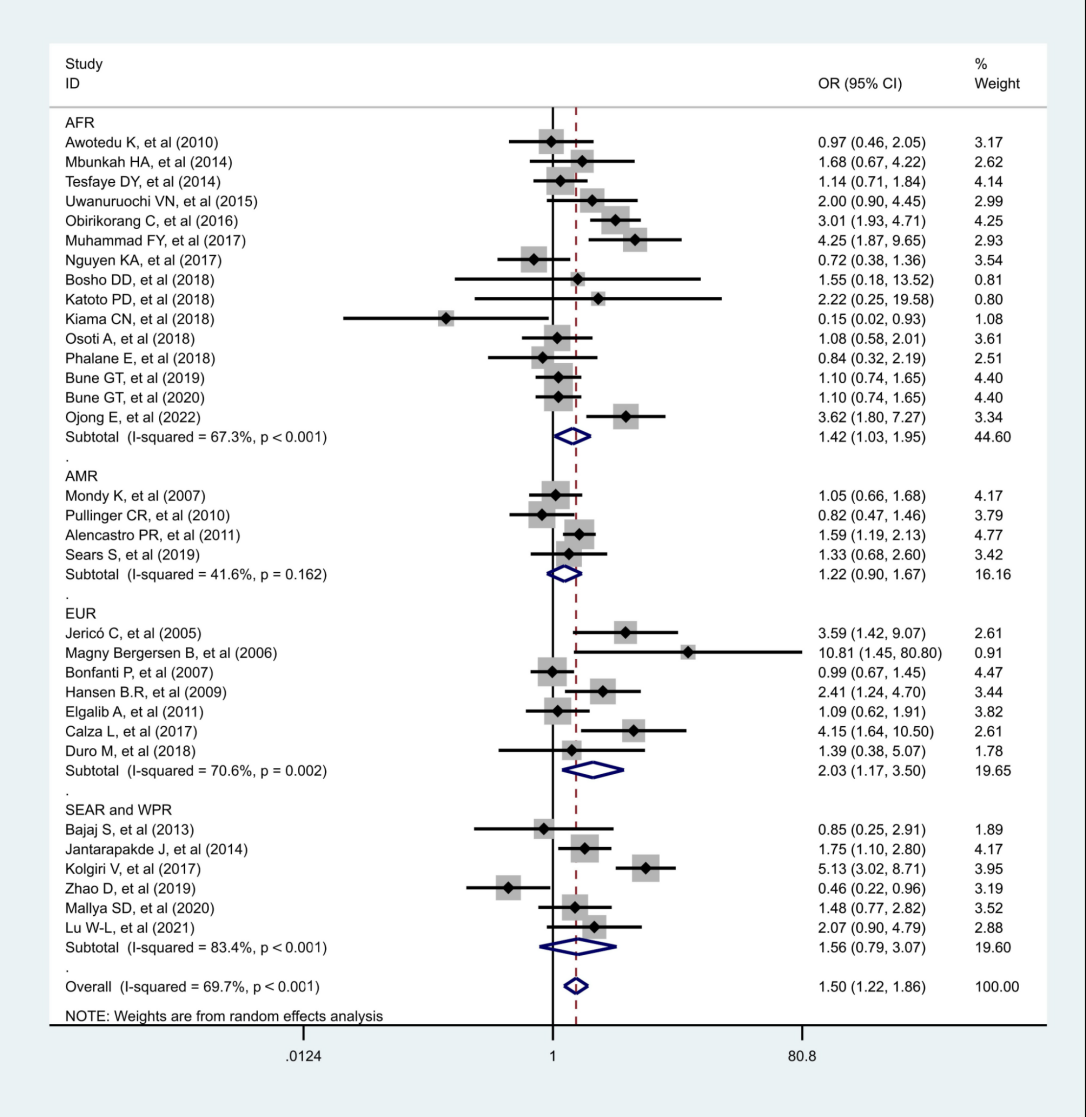
Forest plot showing the pooled OR of Metabolic syndrome associated with antiretroviral therapy by regions. AFR: African region; AMR: Region of the Americas; EUR: European region; SEAR and WPR: South-East Asia and Western Pacific regions. (For each study the black box represents the study estimate and the horizontal bar represent the 95% CI. The diamond at the lower tail is the pooled effect estimates from random effect model.)

Thirty-two studies reported odds of MetS between ART status among PLWHA. The global pooled odds ratio for developing MetS in according to ART exposure was approximately 1.504 (95% CI, 1.217–1.859, *p* < 0.001; I^2^ = 69.7%). EUR showed the highest pooled odds ratio of MetS 2.027(95% CI, 1.174–3.499, *p* = 0.01; I^2^ = 70.6%) followed by SEAR and WPR 1.556(95% CI, 0.788–3.074, *p* = 0.203; I^2^ = 83.4%); AFR 1.417(95% CI, 1.029–1.952, *p* = 0.033; I^2^ = 67.3%), and AMR 1.225(95% CI, 0.900-1.667, *p* = 0.197, I^2^ = 41.6%), respectively (Fig. 4). In sensitivity analysis, pooled odds ratios of MetS in according with ART exposure were slightly different by age groups and study quality. Sensitivity analysis, meta-regression and subgroup analysis are shown in additional file 9.

### Publication bias

An analysis of publication bias by Egger’s test and Funnel plot showed that there was no evidence of publication bias in the estimation of pooled odds ratio. The assessments of publication bias are shown in additional file 10.

## Discussion

This meta-analysis among PLWHA revealed the global pooled prevalence of MetS at 25.26%, specific pooled prevalence among PLWHA under ART at 25.61%, and specific pooled prevalence among non-treat PLWHA at 18.48%, and the global pooled incidence rate of MetS was 9.19 per 100 person-years. The pooled prevalence varied across the regions; the AMR (30.4%), and the SEAR and WPR (26.7%) had the highest prevalence and the geographic distribution is similar with a meta-analysis of MetS in the global general population [53]. Of 1,129 prevalence data (28,193,768 participants), the prevalence in general population was significantly higher in the Eastern Mediterranean Region/EMR (30.5%), AMR (29.5%), SEAR (29.3%) and WPR (28.6%) [53]. In general, MetS affected patients, causing them to face higher morbidity and mortality, and resulted in an enormous economic burden [54–59]. Several studies suggested that individuals with MetS had higher risk of diabetes by 5 times than non-MetS, while risk for chronic kidney disease, myocardial infarction, stroke, heart failure, cardiogenic shock, death from cardiovascular disease and all-cause deaths ranged from 1.22 to 1.55 times [54–56]. Furthermore, the economic burden of MetS was significant with increased medical and health care costs by 20–60% higher for individuals with MetS than those without MetS [57–59]. However, evidence regarding the risk of MetS affecting these NCDs and mortality in PLWHA as well as economic impact is still insufficient; more research is therefore required.

The AMR, SEAR and WPR showed the greatest prevalence of MetS among HIV-infected patients, and followed by AFR, and EUR. Interestingly, this is despite the fact that the main HIV burden is in sub-Saharan Africa, with a total 25.6 million PLWHA out of a world total of 38 million. Inequality in ART coverage may be involved in the burden of MetS, as ART coverage is highest in Western/Central Europe (79%) and East/Southern Africa (67%) [24]. The significant variation in the geographical distribution of MetS prevalence suggests the presence of population- or country-specific drivers such as social contexts and health-related behaviors [21, 22]. Our study found different characteristics of study population from articles across regions, study population in the AMR were older, had longer duration of HIV infections, and ART exposure than those in other regions and these factors increased the risk of developing MetS in HIV-positive patients, similarly to previous studies [49, 60–66]. In addition, 13 of the 21 studies in the AMR were conducted in the United States (U.S.), and a larger proportion of African Americans, ranging from 19 to 83% [37, 67] were reported among those studies. The prevalence of MetS in this region may be influenced by race and low socioeconomic status (SES), which corresponds to the U.S. national survey data that found the highest MetS prevalence among Black/African Americans (27.5%), those with low income (16.8%) and low education (17.6%) [68]. Since African Americans have had the lowest median household income in the U.S for the previous 50 years, they are the poorest ethnic group in this country [69]. Poverty is a social determinant of health and induces risk behaviors leading to a high prevalence of diseases and poor health outcomes among this marginalized group [70].

The high MetS prevalence in the SEAR and WPR may likely be explained by socio-cultural epidemiological transition. The socio-cultural epidemiological change is one of the main underlying risk factors for the increase in NCDs in the Asia-Pacific region. This region has undergone drastic changes in the population’s lifestyles as a result of economic development and rapid urbanization [71, 72]. Evidence indicates that several lifestyle risk factors for MetS and NCDs such as low physical activity/sedentary life style, unhealthy diet, increased consumption of alcohol and tobacco, are linked to urbanization [71, 73]. Regarding SES, studies in South Asian countries revealed that people with high SES had a higher prevalence of the MetS [74, 75]. On the other hand, studies in China showed a significantly higher prevalence of MetS among individuals with a lower SES; they were more likely to be exposed to unhealthy food and behavior, unaware of disease prevention and control and had inequalities in access to healthcare [76, 77]. This relationship is quite complex and differs between developing and developed countries. SES is positively associated with MetS in developing countries [74, 75], but adversely correlated with MetS in developed countries [78, 79]. This situation likely be explained by social determinant of health and health literacy concept. It is well recognized that lower SES people tended to live in unhealthy lifestyles and consume unhealthy foods due to lack of knowledge, affordability and social opportunities and caused greater risk of Mets compare to higher SES. In developing countries, the effects of SES on Mets may be even worse. Firstly, food choices in developing countries are mostly rich of oil, and carbohydrates, and less protein compare to developed countries. Secondly, public and social welfare such as outreach health education, health care service for lower SES in developing countries may slightly less than developed countries [74–79].

In this meta-analysis, HIV infection and ART exposure had a statistically significant association with the risk of MetS. According to this analysis, HIV-infected patients had a 1.6 times higher chance of developing MetS than the general population. Surprisingly, five studies showed that the prevalence of MetS was lower in the HIV-infected patients as compared to the uninfected individuals [28, 80–83]. There are two possible explanations for the lower MetS prevalence among HIV-infected participants. Firstly, compared to non-HIV infected persons, HIV infected persons had lower obesity and blood pressure [28, 80, 81, 83]. Additionally, in these studies, HIV-negative individuals do not have the same access to preventative healthcare as HIV-positive patients do leading to regularly receive education on risk reduction and nutrition counseling in HIV clinics [28, 80–83]. Regarding the effect of ART, patients receiving ART were at approximately 1.5 times more likely to develop MetS than those not receiving treatment. By systematically reviewing the literature, the risk of MetS increased with the use of ART, namely the nucleoside reverse transcriptase inhibitors/NRTIs (i.e., didanosine, stavudine, and abacavir) [28, 52, 60, 82, 84–86], the non-nucleoside reverse transcriptase inhibitors/NNRTIs (i.e., efavirenz) [87] and the protease inhibitors/PIs (i.e., ritonavir, lopinavir, darunavir, atazanavir, nelfinavir, and saquinavir) [28, 84, 88, 89]. The use of the integrase strand transfer inhibitors/INSTIs like raltegravir and dolutegravir did not adversely influence MetS and may possibly even have a beneficial impact [89]. On the contrary, one study revealed that the MetS risk could be increased with the use of dolutegravir. Nevertheless, because INSTIs are relatively new to sub-Saharan Africa and there hasn’t been much experience with them at the time the study was conducted, more research is recommended to confirm this finding [90].

This meta-analysis suggests effects of HIV infection and ART exposure on significant excess burden of MetS among PLWHA. Persistent immune activation and chronic inflammation due to HIV lead to release of inflammatory cytokines, which cause metabolic complications and organ damage [9, 10]. Consequently, patients with late HIV diagnosis who do not receive treatment may be at risk for developing MetS and other NCDs [9, 10, 15–18]. According to this systematic review, patients with advanced stage of HIV infection [52, 91, 92], low CD4 cell count [52, 88, 93] and high HIV viral load [28, 52, 90, 94] were more likely to develop MetS. Therefore, routine HIV screening of general population and frequent HIV testing of persons at high risk of contracting HIV are necessary to reduce late HIV diagnosis and its consequences, and enable patients to receive immediate ART initiation [95]. The effective ART results in rapid control of HIV infection and restoration of immune function, leading to prohibit in the advance stage of disease and prevent various complications [95]. However, cumulative toxicities from ART exposure for a long time can cause MetS [65, 66]. Although less toxicity ART has been prescribed in many countries, however, in order to lessen the burden of HIV-NCD comorbidities, screening, early detection of MetS and other traditional risk factors of NCDs, behavior change counseling, and routine monitoring are extremely important [96]. These findings support the integration of HIV/AIDS and NCD; currently, there are growing calls for integrated healthcare services for HIV/AIDS and NCD in order to cope with a high burden of multimorbidity in PLWHA, particularly in low- and middle-income countries [2, 97]. Integration of HIV/AIDS and NCD would benefit patients and the health system such as reducing duplication and fragmentation of services, improving resource use, and supporting patients to remain in care by lowering costs and inconvenience for those with multiple morbidities [98–100].

### Strengths and limitations

#### Strengths

This is the comprehensive review and meta-analysis which attempt to describe the MetS burden and estimate the common effect size of HIV infection and ART on MetS development at the global and regional levels in the HAART era. To maximize the robustness and reliability of the applied methodology, this review strictly complies with the PRISMA guidelines. We also conducted a thorough search across different databases using replicable criteria to capture the greatest number of studies on MetS burden worldwide.

#### Limitations

Using Cohen’s kappa to measure agreement in selecting the abstracts of both researchers, the kappa was 0.829, which was seen as an almost perfect agreement. Although, the kappa for full texts selection was substantial agreement, 0.669 due to the definitions of MetS. However, the disagreements were resolved by the third researcher and senior investigator to reduce the possibility of excluding the relevant studies. The analysis of the individual MetS burden subcomponents was limited given the inadequate reporting in the included studies. Further study is required to understand which MetS subcomponent is most prevalent and influences MetS development by HIV status and ART exposure. Only five cohort studies were included for estimating the pooled incidence. Therefore, estimates of common effect size for HIV infection and ART use were derived from cross-sectional and case-control studies which cannot confirm the causality between HIV infection, ART, and the development of MetS. Our study was unable to determine the common effect size of individual ART on the risk of MetS because of the difference in designs and definitions of ART exposure. Bias should be taken into consideration when evaluating the effect of individual ART; the channeling bias could have occurred if patients perceived to be at high risk for MetS were either started on or switched to ART regimens thought to be MetS protective. Furthermore, not all studies reported the duration of ART in study subjects, and the duration of ART varied among studies, which increased the heterogeneity between studies. Data were missing on some characteristics that could be used in meta-regression. In addition, the inconsistent number of studies across subgroup precluded meta-regression analyses to investigate the possible contribution of each variable to heterogeneity. Due to variations in sample characteristics, sample selection, HIV treatment, and methodological designs of the included studies, the estimation of the pooled prevalence/incidence and pooled odds ratio may have been affected by this heterogeneity, as evidenced by the very high I^2^ statistic.

## Conclusion

The burden of MetS in global HIV-infected population is high, particularly in region of the Americas, the South-East Asian and Western Pacific regions. These findings suggest HIV infection and ART exposure appear to contribute to a significant excess burden of MetS, which may lead to an increase in the burden of other NCDs. Therefore, early screening of MetS and MetS subcomponents among PLWHA is essential for reducing costs associated with the HIV and NCD epidemics, especially in the era of an increased aging population. It is important to raise public awareness and understanding at the global and country levels for integrating NCDs prevention and control into HIV/AIDS strategies, policies and programs with a focus on people living with and at risk of HIV and HIV-NCDs comorbidities.

## Electronic supplementary material

Below is the link to the electronic supplementary material.

Additional File 1

Additional File 2

Additional File 3

Additional File 4

Additional File 5

Additional File 6

Additional File 7

Additional File 8

Additional File 9

Additional File 10

## Data Availability

The datasets used and/or analysed during the current study available from the corresponding author on reasonable request.

## Abbreviations

AFR: African region
AHRQ: Agency for Healthcare Research and Quality
AMR: Region of the Americas
ART: Antiretroviral therapy/Antiretroviral treatment
BMI: Body mass index
EMR: Eastern Mediterranean Region
EUR: European region
HAART: Highly Active Antiretroviral Therapy
HIV: Human Immunodeficiency Virus
INSTIs: Integrase strand transfer inhibitors
MetS: Metabolic syndrome
NCDs: Noncommunicable diseases
N-O: Scale Newcastle-Ottawa scale
NRTIs: Nucleoside reverse transcriptase inhibitors
NNRTIs: Non-nucleoside reverse transcriptase inhibitors
OR: Odds ratio
PIs: Protease inhibitors
PLWHA: People living with HIV/AIDS
REML: Restricted maximum likelihood
SEAR and WPR: South-East Asia and Western Pacific regions
SES: Socioeconomic status
TCI: Thai-journal citation index
TDC: Thai digital collection
ThaiJO: Thai journals online
TJI: Thai journal index
U.S: The United State
