# Supplementary material for: Burden of metabolic syndrome in the global adult HIV-infected population: a systematic review and meta-analysis
Source: BMC Public Health. 2024 Sep 28;24:2657. doi: 10.1186/s12889-024-20118-3 (PMC11438355; doi:10.1186/s12889-024-20118-3)
Supplement: Supplementary file 1 — Additional File 1 [file 12889_2024_20118_MOESM1_ESM.docx]

**Additional file 1**

**Table S1 Definition of review outcomes.**

|  | **Definitions** |
| --- | --- |
| **Metabolic syndrome (MetS)** | MetS is specified as the cluster of interrelated risk factors including glucose intolerance, hypertension, dyslipidemia and obesity. The diagnostic criteria for identifying MetS for this study include: [1-4] |
|  | NCEP/ATP III criteria: this guideline consists of three or more of the following; waist circumference of more than 102 cm (40 in) in men and more than 88 cm (35 in) in women, triglyceride levels of at least 150 mg per dL (1.70 mmol per L), high-density lipoprotein cholesterol levels of less than 40 mg per dL (1.04 mmol per L) in men and less than 50 mg per dL (1.30 mmol per L) in women, blood pressure of at least 130/85 mm Hg, and fasting glucose levels of at least 110 mg per dL (6.10 mmol per L). |
|  | IDF criteria: a central obesity and any 2 or 4 additional risk factors; raised triglyceride (≥1.7 mmol/L (150 mg/dL), reduced HDL-cholesterol (male <1.03 mmol/L (40 mg/dL), women <1.29 mmol/L (50 mg/dL)), raised blood pressure (≥130/≥85 mm Hg) or antihypertensive drug, raised fasting plasma glucose (≥5.6 mmol/L (100 mg/dL)) or diagnosed type 2 DM. |
|  | EGIR criteria: a plasma insulin >75th percentile with any 2 risk factors; waist circumference (men ≥94 cm, women ≥80 cm), triglyceride (≥150 mg/dL) and/or HDL-C (<39 mg/dL) in men or women, blood pressure ≥140 mm Hg or on antihypertensive medication, impaired glucose tolerance or impaired fasting glucose but not DM. |
|  | WHO criteria: diagnosis of insulin resistance (either, type 2 DM, impaired fasting glucose, impaired glucose tolerance, normal fasting glucose levels (110 mg/dL) or glucose uptake below the lowest quartile). In addition, diagnosis of 2 other risk factors is sufficient. Antihypertensive medication and/or high blood pressure (≥140 mm Hg systolic or ≥90 mm Hg diastolic), plasma triglycerides ≥150 mg/dL (≥1.7 mmol/L), HDL cholesterol (men 35 mg/dL (0.9 mmol/L), women 39 mg/dL (1.0 mmol/L)), BMI 30 kg/m^2^ and/or waist: hip ratio (men >0.9, women >0.85), urinary albumin excretion rate (≥20 g/min) or albumin: creatinine ratio (≥30 mg/g). |
|  | The AHA and NHLBI criteria: having three or more of the following five components: abdominal obesity (waist circumference≥90 cm for men, ≥80 cm for women), hypertriglyceridemia (≥150 mg/dL), low HDL (men < 40 mg/dL, women < 50 mg/dL), high blood pressure (≥130/85 mmHg), and high fasting plasma glucose (FPG≥100 mg/dL) |
|  | JIS criteria: three of five criteria had to be fulfilled for a person to be identified with MS. The clinical criteria included elevated waist circumference ≥94 cm for men and≥80 cm for women), elevated serum triglycerides ≥1.7 mmol/L), reduced HDL-C ≤1.0 mmol/L for men and ≤1.3 mmol/L for women), elevated blood pressure (systolic ≥130 and/or diastolic≥85 mm/Hg), and elevated fasting plasma glucose ≥5.6 mmol/L |
|  | HJSS criteria: which is the presence of any three of the five following risk factors; 1) waist circumference > 94cm for men or > 80cm for women, 2) triglycerides > 1.7 mmol/L or specific treatment for this abnormality, 3) HDL-Cholesterol < 1.03 mmol/L for men or < 1.29 mmol/L for women or specific treatment for this abnormality, 4) elevated blood pressure > 130/85 mmHg or treatment of previously diagnosed hypertension and 5) elevated fasting glucose > 5.6 mmol/L or treatment of previously diagnosed diabetes. |

NCEP/ATP III, the National Cholesterol Education Program’s Adult Treatment Panel III; IDF, International Diabetes Federation; EGIR, European Group for Study of Insulin Resistance; WHO, World Health Organization; the AHA and NHLBI, the American Heart Association and National Heart, Lung, and Blood Institute; JIS, Joint Interim Statement; HJSS, the harmonized Joint Scientific Statement.

**References**

1. Grundy SM, Brewer Jr HB, Cleeman JI, Smith Jr SC, Lenfant C. Definition of metabolic syndrome: report of the National Heart, Lung, and Blood Institute/American Heart Association conference on scientific issues related to definition. Circulation. 2004;109(3):433-8.

2. Grundy SM, Cleeman JI, Daniels SR, Donato KA, Eckel RH, Franklin BA, et al. Diagnosis and management of the metabolic syndrome: an American Heart Association/National Heart, Lung, and Blood Institute scientific statement. Circulation. 2005;112(17):2735-52.

3. Grundy SM. Metabolic syndrome scientific statement by the American Heart Association and the National Heart, Lung, and Blood Institute. Arterioscler Thromb Vasc Biol. 2005; 25:2243–2244.

4. Alberti KG, Eckel RH, Grundy SM, Zimmet PZ, Cleeman JI, Donato KA, Fruchart JC, et al. Harmonizing the metabolic syndrome: a joint interim statement of the International Diabetes Federation Task Force on Epidemiology and Prevention; National Heart, Lung, and Blood Institute; American Heart Association; World Heart Federation; International Atherosclerosis Society; and International Association for the Study of Obesity Circulation. 2009; 120(16):1640–1645.
