# Supplementary material for: Burden of metabolic syndrome in the global adult HIV-infected population: a systematic review and meta-analysis
Source: BMC Public Health. 2024 Sep 28;24:2657. doi: 10.1186/s12889-024-20118-3 (PMC11438355; doi:10.1186/s12889-024-20118-3)
Supplement: Supplementary file 2 — Additional File 2 [file 12889_2024_20118_MOESM2_ESM.docx]

**Additional file 2**

**Table S2.1 Search results.**

| **International databases** | **Frequency** |  | |  |
| --- | --- | --- | --- | --- |
| Scopus | 62,002 |  | |  |
| Embase | 10,377 |  | |  |
| PubMed/MEDLINE | 5,550 | **Summary: International databases** | | |
| EBSCO | 3,475 | Number of duplicates | | 11,253 |
| **Total** | 81,404 | Number of abstracts screened | | 70,151 |
|  |  |  | |  |
| **Thai databases** | **Frequency** |  | |  |
| Thai-journal citation index (TCI) | 2,538 |  | |  |
| Thai journal index (TJI) | 1,163 |  | |  |
| Thai digital collection (TDC) | 434 | **Summary: Thai databases** | | |
| Thai journals online (Thai JO) | 51 | Number of duplicates | | 837 |
| **Total** | 4,186 | Number of abstracts screened | | 3,349 |
|  |  |  | |  |
| **Summary: Both International and Thai databases** | | | | |
|  | | | **Frequency** | |
| Total | | | 85,590 | |
| Number of duplicates | | | 12,090 | |
| **Number of abstracts screened** | | | 73,500 | |

**Table S2.2 Search strategy and terms guide.**

| **Search** | **Search terms** |
| --- | --- |
|  | **Population** |
| #1 | (HIV) OR (HIV-infected adults) OR (HIV-infected individuals) OR (HIV-infected patients) OR (HIV-positive adults) OR (HIV-positive individuals) OR (HIV-positive patients) OR (People living with HIV) OR (PLWHIV) OR (PLWHA) OR (persons living with HIV) |
|  | **Intervention/Comparison** |
| #2 | (ART) OR (ARV) OR (HAART) OR (antiretroviral therapy) OR (treated with ART) OR (treated with ARV) OR (treated with HAART) OR (treated with antiretroviral therapy) OR (on ART) OR (on ARV) OR (on HAART) OR (on antiretroviral therapy) OR (untreated HIV) OR (naïve) OR (HIV-uninfected individuals) OR (HIV-uninfected people) OR (HIV-negative individuals) OR (HIV-negative people) |
|  | **Outcomes** |
| #3 | (metabolic syndrome) OR (metabolic complication) OR (cardiovascular syndrome) OR (cardiovascular risk factors) |
| #4 | (diabetes mellitus) OR (diabetes) OR (DM) OR (hyperglycemia) |
| #5 | (cardiovascular disease) OR (cardiovascular event) OR (CVD) OR (coronary artery disease) OR (CAD) OR (myocardial infraction) OR (MI) OR (ischemic heart disease) OR (IHD) OR (heart disease) OR (stroke) OR (cerebrovascular disease) OR (cerebrovascular event) |
| #6 | (kidney diseases) OR (chronic kidney disease) OR (CKD) OR (chronic kidney failure) OR (chronic renal failure) OR (end stage renal disease) OR (ESRD) OR (abnormal eGFR) OR (reduced eGFR) OR (kidney dysfunction) |
| #7 | #3 OR #4 OR #5 OR #6 |
|  | **Population and Intervention/Comparator** |
| #8 | #1 AND #2 |
|  | **Population and Outcome** |
| #9 | #1 AND #7 |
|  | **Intervention/Comparator and Outcome** |
| #10 | #2 AND #7 |
|  | **Population, Intervention/Comparator and Outcome** |
| #11 | #1 AND #2 AND #7 Limit 01/01/2000 to 31/12/2023 |

**Table S2.3 Scopus searching.**

| **No** | **Search Terms** | **Results** |
| --- | --- | --- |
| 11 | ( ALL ( hiv  OR  "HIV-infected adults"  OR  "HIV-infected individuals"  OR  "HIV-infected patients"  OR  "HIV-positive adults"  OR  "HIV-positive individuals"  OR  hiv-positive  AND  patients  OR  "People living with HIV"  OR  plwhiv  OR  plwha  OR  "persons living with HIV" ) )  AND  ( ALL ( art  OR  arv  OR  haart  OR  "antiretroviral therapy"  OR  "treated with ART"  OR  "treated with ARV"  OR  "treated with HAART"  OR  "treated with antiretroviral therapy"  OR  "on ART"  OR  "on ARV"  OR  "on HAART"  OR  "on antiretroviral therapy"  OR  "untreated HIV"  OR  naïve  OR  "HIV-uninfected individuals"  OR  "HIV-uninfected people"  OR  "HIV-negative individuals"  OR  "HIV-negative people" ) )  AND  ( ( ALL ( "metabolic syndrome"  OR  "metabolic complication"  OR  "cardiovascular syndrome"  OR  "cardiovascular risk factors" ) )  OR  ( ALL ( "diabetes mellitus"  OR  diabetes  OR  dm  OR  hyperglycemia ) )  OR  ( ALL ( "cardiovascular disease"  OR  "cardiovascular event"  OR  cvd  OR  "coronary artery disease"  OR  cad  OR  "myocardial infraction"  OR  mi  OR  "ischemic heart disease"  OR  ihd  OR  "heart disease"  OR  stroke  OR  "cerebrovascular disease"  OR  "cerebrovascular event" ) )  OR  ( ALL ( "kidney diseases"  OR  "chronic kidney disease"  OR  ckd  OR  "chronic kidney failure"  OR  "chronic renal failure"  OR  "end-stage renal disease"  OR  esrd  OR  "abnormal eGFR"  OR  "reduced eGFR"  OR  "kidney dysfunction" ) ) ) ...View More | **62,002** |
| 10 | ( ALL ( art  OR  arv  OR  haart  OR  "antiretroviral therapy"  OR  "treated with ART"  OR  "treated with ARV"  OR  "treated with HAART"  OR  "treated with antiretroviral therapy"  OR  "on ART"  OR  "on ARV"  OR  "on HAART"  OR  "on antiretroviral therapy"  OR  "untreated HIV"  OR  naïve  OR  "HIV-uninfected individuals"  OR  "HIV-uninfected people"  OR  "HIV-negative individuals"  OR  "HIV-negative people" ) )  AND  ( ( ALL ( "metabolic syndrome"  OR  "metabolic complication"  OR  "cardiovascular syndrome"  OR  "cardiovascular risk factors" ) )  OR  ( ALL ( "diabetes mellitus"  OR  diabetes  OR  dm  OR  hyperglycemia ) )  OR  ( ALL ( "cardiovascular disease"  OR  "cardiovascular event"  OR  cvd  OR  "coronary artery disease"  OR  cad  OR  "myocardial infraction"  OR  mi  OR  "ischemic heart disease"  OR  ihd  OR  "heart disease"  OR  stroke  OR  "cerebrovascular disease"  OR  "cerebrovascular event" ) )  OR  ( ALL ( "kidney diseases"  OR  "chronic kidney disease"  OR  ckd  OR  "chronic kidney failure"  OR  "chronic renal failure"  OR  "end-stage renal disease"  OR  esrd  OR  "abnormal eGFR"  OR  "reduced eGFR"  OR  "kidney dysfunction" ) ) ) ...View More | 538,786 |
| 9 | ( ALL ( hiv  OR  "HIV-infected adults"  OR  "HIV-infected individuals"  OR  "HIV-infected patients"  OR  "HIV-positive adults"  OR  "HIV-positive individuals"  OR  hiv-positive  AND  patients  OR  "People living with HIV"  OR  plwhiv  OR  plwha  OR  "persons living with HIV" ) )  AND  ( ( ALL ( "metabolic syndrome"  OR  "metabolic complication"  OR  "cardiovascular syndrome"  OR  "cardiovascular risk factors" ) )  OR  ( ALL ( "diabetes mellitus"  OR  diabetes  OR  dm  OR  hyperglycemia ) )  OR  ( ALL ( "cardiovascular disease"  OR  "cardiovascular event"  OR  cvd  OR  "coronary artery disease"  OR  cad  OR  "myocardial infraction"  OR  mi  OR  "ischemic heart disease"  OR  ihd  OR  "heart disease"  OR  stroke  OR  "cerebrovascular disease"  OR  "cerebrovascular event" ) )  OR  ( ALL ( "kidney diseases"  OR  "chronic kidney disease"  OR  ckd  OR  "chronic kidney failure"  OR  "chronic renal failure"  OR  "end-stage renal disease"  OR  esrd  OR  "abnormal eGFR"  OR  "reduced eGFR"  OR  "kidney dysfunction" ) ) ) ...View More | 178,306 |
| 8 | ( ALL ( hiv  OR  "HIV-infected adults"  OR  "HIV-infected individuals"  OR  "HIV-infected patients"  OR  "HIV-positive adults"  OR  "HIV-positive individuals"  OR  hiv-positive  AND  patients  OR  "People living with HIV"  OR  plwhiv  OR  plwha  OR  "persons living with HIV" ) )  AND  ( ALL ( art  OR  arv  OR  haart  OR  "antiretroviral therapy"  OR  "treated with ART"  OR  "treated with ARV"  OR  "treated with HAART"  OR  "treated with antiretroviral therapy"  OR  "on ART"  OR  "on ARV"  OR  "on HAART"  OR  "on antiretroviral therapy"  OR  "untreated HIV"  OR  naïve  OR  "HIV-uninfected individuals"  OR  "HIV-uninfected people"  OR  "HIV-negative individuals"  OR  "HIV-negative people" ) ) ...View More | 221,211 |
| 7 | ( ALL ( "metabolic syndrome"  OR  "metabolic complication"  OR  "cardiovascular syndrome"  OR  "cardiovascular risk factors" ) )  OR  ( ALL ( "diabetes mellitus"  OR  diabetes  OR  dm  OR  hyperglycemia ) )  OR  ( ALL ( "cardiovascular disease"  OR  "cardiovascular event"  OR  cvd  OR  "coronary artery disease"  OR  cad  OR  "myocardial infraction"  OR  mi  OR  "ischemic heart disease"  OR  ihd  OR  "heart disease"  OR  stroke  OR  "cerebrovascular disease"  OR  "cerebrovascular event" ) )  OR  ( ALL ( "kidney diseases"  OR  "chronic kidney disease"  OR  ckd  OR  "chronic kidney failure"  OR  "chronic renal failure"  OR  "end-stage renal disease"  OR  esrd  OR  "abnormal eGFR"  OR  "reduced eGFR"  OR  "kidney dysfunction" ) ) ...View More | 7,299,793 |
| 6 | ALL ( "kidney diseases"  OR  "chronic kidney disease"  OR  ckd  OR  "chronic kidney failure"  OR  "chronic renal failure"  OR  "end-stage renal disease"  OR  esrd  OR  "abnormal eGFR"  OR  "reduced eGFR"  OR  "kidney dysfunction" ) | 807,072 |
| 5 | ALL ( "cardiovascular disease"  OR  "cardiovascular event"  OR  cvd  OR  "coronary artery disease"  OR  cad  OR  "myocardial infraction"  OR  mi  OR  "ischemic heart disease"  OR  ihd  OR  "heart disease"  OR  stroke  OR  "cerebrovascular disease"  OR  "cerebrovascular event" ) | 5,065,666 |
| 4 | ALL ( "diabetes mellitus"  OR  diabetes  OR  dm  OR  hyperglycemia ) | 2,725,157 |
| 3 | ALL ( "metabolic syndrome"  OR  "metabolic complication"  OR  "cardiovascular syndrome"  OR  "cardiovascular risk factors" ) | 790,682 |
| 2 | ALL ( art  OR  arv  OR  haart  OR  "antiretroviral therapy"  OR  "treated with ART"  OR  "treated with ARV"  OR  "treated with HAART"  OR  "treated with antiretroviral therapy"  OR  "on ART"  OR  "on ARV"  OR  "on HAART"  OR  "on antiretroviral therapy"  OR  "untreated HIV"  OR  naïve  OR  "HIV-uninfected individuals"  OR  "HIV-uninfected people"  OR  "HIV-negative individuals"  OR  "HIV-negative people" ) | 4,529,012 |
| 1 | ALL ( hiv  OR  "HIV-infected adults"  OR  "HIV-infected individuals"  OR  "HIV-infected patients"  OR  "HIV-positive adults"  OR  "HIV-positive individuals"  OR  hiv-positive  AND  patients  OR  "People living with HIV"  OR  plwhiv  OR  plwha  OR  "persons living with HIV" ) | 797,619 |

**Table S2.4 Embase searching.**

| **No** | **Search Terms** | **Results** |
| --- | --- | --- |
| 11 | #1 AND #2 AND #7 | **10,377** |
| 10 | #2 AND #7 | 45,657 |
| 9 | #1 AND #7 | 30,686 |
| 8 | #1 AND #2 |  |
| 7 | #3 OR #4 OR #5 OR #6 | 3,497,526 |
| 6 | 'kidney diseases' OR 'chronic kidney disease' OR 'ckd' OR 'chronic kidney failure' OR 'chronic renal failure' OR 'end-stage renal disease' OR 'esrd' OR 'abnormal egfr' OR 'reduced egfr' OR 'kidney dysfunction':ui | 319,995 |
| 5 | 'cardiovascular disease' OR 'cardiovascular event' OR 'cvd' OR 'coronary artery disease' OR 'cad' OR 'myocardial infraction' OR 'mi' OR 'ischemic heart disease' OR 'ihd' OR 'heart disease' OR 'stroke' OR 'cerebrovascular disease' OR 'cerebrovascular event':ui | 2,148,953 |
| 4 | 'diabetes mellitus' OR 'diabetes' OR 'dm' OR 'hyperglycemia':ui | 1,415,467 |
| 3 | 'metabolic syndrome' OR 'metabolic complication' OR 'cardiovascular syndrome' OR 'cardiovascular risk factors':ui | 124,859 |
| 2 | 'art' OR 'arv' OR 'haart' OR 'antiretroviral therapy' OR 'treated with art' OR 'treated with arv' OR 'treated with haart' OR 'treated with antiretroviral therapy' OR 'on art' OR 'on arv' OR 'on haart' OR 'on antiretroviral therapy' OR 'untreated hiv' OR 'naïve' OR 'hiv-uninfected individuals' OR 'hiv-uninfected people' OR 'hiv-negative individuals' OR 'hiv-negative people':ui | 576,027 |
| 1 | 'hiv' OR 'hiv-infected adults' OR 'hiv-infected individuals' OR 'hiv-infected patients' OR 'hiv-positive adults' OR 'hiv-positive individuals' OR 'hiv-positive patients' OR 'people living with hiv' OR 'plwhiv' OR 'plwha' OR 'persons living with hiv':ui | 495,966 |

**Table S2.5 PubMed/MEDLINE searching.**

| **No** | **Search Terms** | **Search Details** | **Results** |
| --- | --- | --- | --- |
| 11 | #1 AND #2 AND #7 | ("hiv"[MeSH Terms] OR "hiv"[All Fields] OR ("HIV-infected"[All Fields] AND "adult"[MeSH Terms]) OR ("HIV-infected"[All Fields] AND "patients"[MeSH Terms]) OR (("hiv seropositivity"[MeSH Terms] OR ("hiv"[All Fields] AND "seropositivity"[All Fields]) OR "hiv seropositivity"[All Fields] OR ("hiv"[All Fields] AND "positive"[All Fields]) OR "HIV-positive"[All Fields]) AND "adult"[MeSH Terms]) OR (("hiv seropositivity"[MeSH Terms] OR ("hiv"[All Fields] AND "seropositivity"[All Fields]) OR "hiv seropositivity"[All Fields] OR ("hiv"[All Fields] AND "positive"[All Fields]) OR "HIV-positive"[All Fields]) AND "patients"[MeSH Terms]) OR ((("people s"[All Fields] OR "peopled"[All Fields] OR "peopling"[All Fields] OR "persons"[MeSH Terms] OR "persons"[All Fields] OR "people"[All Fields] OR "peoples"[All Fields]) AND ("lived"[All Fields] OR "lives"[All Fields] OR "living"[All Fields] OR "livings"[All Fields])) AND "hiv"[MeSH Terms]) OR ((("person s"[All Fields] OR "personable"[All Fields] OR "personableness"[All Fields] OR "personal"[All Fields] OR "personalisation"[All Fields] OR "personalise"[All Fields] OR "personalised"[All Fields] OR "personalising"[All Fields] OR "personality"[MeSH Terms] OR "personality"[All Fields] OR "personalities"[All Fields] OR "personality s"[All Fields] OR "personalization"[All Fields] OR "personalize"[All Fields] OR "personalized"[All Fields] OR "personalizes"[All Fields] OR "personalizing"[All Fields] OR "personally"[All Fields] OR "personals"[All Fields] OR "persons"[MeSH Terms] OR "persons"[All Fields] OR "person"[All Fields]) AND ("lived"[All Fields] OR "lives"[All Fields] OR "living"[All Fields] OR "livings"[All Fields])) AND "hiv"[MeSH Terms])) AND ("art"[MeSH Terms] OR "art"[All Fields] OR ("arv"[Journal] OR "arv"[All Fields]) OR ("antiretroviral therapy, highly active"[MeSH Terms] OR ("antiretroviral"[All Fields] AND "therapy"[All Fields] AND "highly"[All Fields] AND "active"[All Fields]) OR "highly active antiretroviral therapy"[All Fields] OR "haart"[All Fields] OR "haarts"[All Fields]) OR (("anti retroviral agents"[Pharmacological Action] OR "anti retroviral agents"[MeSH Terms] OR ("anti retroviral"[All Fields] AND "agents"[All Fields]) OR "anti retroviral agents"[All Fields] OR "antiretroviral"[All Fields] OR "antiretrovirally"[All Fields] OR "antiretrovirals"[All Fields]) AND ("therapeutics"[MeSH Terms] OR "therapeutics"[All Fields] OR "therapies"[All Fields] OR "therapy"[MeSH Subheading] OR "therapy"[All Fields] OR "therapy s"[All Fields] OR "therapys"[All Fields])) OR (("therapy"[MeSH Subheading] OR "therapy"[All Fields] OR "treat"[All Fields] OR "therapeutics"[MeSH Terms] OR "therapeutics"[All Fields] OR "treating"[All Fields] OR "treated"[All Fields] OR "treats"[All Fields]) AND "art"[MeSH Terms]) OR (("therapy"[MeSH Subheading] OR "therapy"[All Fields] OR "treat"[All Fields] OR "therapeutics"[MeSH Terms] OR "therapeutics"[All Fields] OR "treating"[All Fields] OR "treated"[All Fields] OR "treats"[All Fields]) AND "antiretroviral therapy, highly active"[MeSH Terms]) OR ((("therapy"[MeSH Subheading] OR "therapy"[All Fields] OR "treat"[All Fields] OR "therapeutics"[MeSH Terms] OR "therapeutics"[All Fields] OR "treating"[All Fields] OR "treated"[All Fields] OR "treats"[All Fields]) AND ("anti retroviral agents"[Pharmacological Action] OR "anti retroviral agents"[MeSH Terms] OR ("anti retroviral"[All Fields] AND "agents"[All Fields]) OR "anti retroviral agents"[All Fields] OR "antiretroviral"[All Fields] OR "antiretrovirally"[All Fields] OR "antiretrovirals"[All Fields])) AND "therapeutics"[MeSH Terms]) OR ("art"[MeSH Terms]) OR ("antiretroviral therapy, highly active"[MeSH Terms]) OR (("anti retroviral agents"[Pharmacological Action] OR "anti retroviral agents"[MeSH Terms] OR ("anti retroviral"[All Fields] AND "agents"[All Fields]) OR "anti retroviral agents"[All Fields] OR "antiretroviral"[All Fields] OR "antiretrovirally"[All Fields] OR "antiretrovirals"[All Fields]) AND "therapeutics"[MeSH Terms]) OR ("untreated"[All Fields] AND "hiv"[MeSH Terms]) OR ("HIV-uninfected"[All Fields] AND "persons"[MeSH Terms]) OR ("HIV-negative"[All Fields] AND "persons"[MeSH Terms])) AND ("metabolic syndrome"[MeSH Terms] OR (("cardiovascular system"[MeSH Terms] OR ("cardiovascular"[All Fields] AND "system"[All Fields]) OR "cardiovascular system"[All Fields] OR "cardiovascular"[All Fields] OR "cardiovasculars"[All Fields]) AND "syndrome"[MeSH Terms]) OR "heart disease risk factors"[MeSH Terms] OR ("diabetes mellitus"[MeSH Terms] OR ("diabetes mellitus"[MeSH Terms] OR "diabetes insipidus"[MeSH Terms]) OR "hyperglycemia"[MeSH Terms]) OR ("cardiovascular diseases"[MeSH Terms] OR "coronary artery disease"[MeSH Terms] OR ("myocardial ischemia"[MeSH Terms] OR "coronary artery disease"[MeSH Terms]) OR "heart diseases"[MeSH Terms] OR "stroke"[MeSH Terms] OR "cerebrovascular disorders"[MeSH Terms]) OR ("kidney diseases"[MeSH Terms] OR "renal insufficiency, chronic"[MeSH Terms] OR "kidney failure, chronic"[MeSH Terms] OR "kidney failure, chronic"[MeSH Terms] OR "kidney failure, chronic"[MeSH Terms] OR "kidney failure, chronic"[MeSH Terms] OR (("abnormal"[All Fields] OR "abnormalities"[MeSH Subheading] OR "abnormalities"[All Fields] OR "congenital abnormalities"[MeSH Terms] OR ("congenital"[All Fields] AND "abnormalities"[All Fields]) OR "congenital abnormalities"[All Fields] OR "abnormality"[All Fields] OR "abnormally"[All Fields] OR "abnormals"[All Fields] OR "abnormities"[All Fields] OR "abnormity"[All Fields]) AND "erbb receptors"[MeSH Terms]) OR (("reduce"[All Fields] OR "reduced"[All Fields] OR "reduces"[All Fields] OR "reducing"[All Fields]) AND "erbb receptors"[MeSH Terms]))) | **5,550** |
| 10 | #2 AND #7 | ("art"[MeSH Terms] OR "art"[All Fields] OR ("arv"[Journal] OR "arv"[All Fields]) OR ("antiretroviral therapy, highly active"[MeSH Terms] OR ("antiretroviral"[All Fields] AND "therapy"[All Fields] AND "highly"[All Fields] AND "active"[All Fields]) OR "highly active antiretroviral therapy"[All Fields] OR "haart"[All Fields] OR "haarts"[All Fields]) OR (("anti retroviral agents"[Pharmacological Action] OR "anti retroviral agents"[MeSH Terms] OR ("anti retroviral"[All Fields] AND "agents"[All Fields]) OR "anti retroviral agents"[All Fields] OR "antiretroviral"[All Fields] OR "antiretrovirally"[All Fields] OR "antiretrovirals"[All Fields]) AND ("therapeutics"[MeSH Terms] OR "therapeutics"[All Fields] OR "therapies"[All Fields] OR "therapy"[MeSH Subheading] OR "therapy"[All Fields] OR "therapy s"[All Fields] OR "therapys"[All Fields])) OR (("therapy"[MeSH Subheading] OR "therapy"[All Fields] OR "treat"[All Fields] OR "therapeutics"[MeSH Terms] OR "therapeutics"[All Fields] OR "treating"[All Fields] OR "treated"[All Fields] OR "treats"[All Fields]) AND "art"[MeSH Terms]) OR (("therapy"[MeSH Subheading] OR "therapy"[All Fields] OR "treat"[All Fields] OR "therapeutics"[MeSH Terms] OR "therapeutics"[All Fields] OR "treating"[All Fields] OR "treated"[All Fields] OR "treats"[All Fields]) AND "antiretroviral therapy, highly active"[MeSH Terms]) OR ((("therapy"[MeSH Subheading] OR "therapy"[All Fields] OR "treat"[All Fields] OR "therapeutics"[MeSH Terms] OR "therapeutics"[All Fields] OR "treating"[All Fields] OR "treated"[All Fields] OR "treats"[All Fields]) AND ("anti retroviral agents"[Pharmacological Action] OR "anti retroviral agents"[MeSH Terms] OR ("anti retroviral"[All Fields] AND "agents"[All Fields]) OR "anti retroviral agents"[All Fields] OR "antiretroviral"[All Fields] OR "antiretrovirally"[All Fields] OR "antiretrovirals"[All Fields])) AND "therapeutics"[MeSH Terms]) OR ("art"[MeSH Terms]) OR ("antiretroviral therapy, highly active"[MeSH Terms]) OR (("anti retroviral agents"[Pharmacological Action] OR "anti retroviral agents"[MeSH Terms] OR ("anti retroviral"[All Fields] AND "agents"[All Fields]) OR "anti retroviral agents"[All Fields] OR "antiretroviral"[All Fields] OR "antiretrovirally"[All Fields] OR "antiretrovirals"[All Fields]) AND "therapeutics"[MeSH Terms]) OR ("untreated"[All Fields] AND "hiv"[MeSH Terms]) OR ("HIV-uninfected"[All Fields] AND "persons"[MeSH Terms]) OR ("HIV-negative"[All Fields] AND "persons"[MeSH Terms])) AND ("metabolic syndrome"[MeSH Terms] OR (("cardiovascular system"[MeSH Terms] OR ("cardiovascular"[All Fields] AND "system"[All Fields]) OR "cardiovascular system"[All Fields] OR "cardiovascular"[All Fields] OR "cardiovasculars"[All Fields]) AND "syndrome"[MeSH Terms]) OR "heart disease risk factors"[MeSH Terms] OR ("diabetes mellitus"[MeSH Terms] OR ("diabetes mellitus"[MeSH Terms] OR "diabetes insipidus"[MeSH Terms]) OR "hyperglycemia"[MeSH Terms]) OR ("cardiovascular diseases"[MeSH Terms] OR "coronary artery disease"[MeSH Terms] OR ("myocardial ischemia"[MeSH Terms] OR "coronary artery disease"[MeSH Terms]) OR "heart diseases"[MeSH Terms] OR "stroke"[MeSH Terms] OR "cerebrovascular disorders"[MeSH Terms]) OR ("kidney diseases"[MeSH Terms] OR "renal insufficiency, chronic"[MeSH Terms] OR "kidney failure, chronic"[MeSH Terms] OR "kidney failure, chronic"[MeSH Terms] OR "kidney failure, chronic"[MeSH Terms] OR "kidney failure, chronic"[MeSH Terms] OR (("abnormal"[All Fields] OR "abnormalities"[MeSH Subheading] OR "abnormalities"[All Fields] OR "congenital abnormalities"[MeSH Terms] OR ("congenital"[All Fields] AND "abnormalities"[All Fields]) OR "congenital abnormalities"[All Fields] OR "abnormality"[All Fields] OR "abnormally"[All Fields] OR "abnormals"[All Fields] OR "abnormities"[All Fields] OR "abnormity"[All Fields]) AND "erbb receptors"[MeSH Terms]) OR (("reduce"[All Fields] OR "reduced"[All Fields] OR "reduces"[All Fields] OR "reducing"[All Fields]) AND "erbb receptors"[MeSH Terms]))) | 14,426 |
| 9 | #1 AND #7 | ("hiv"[MeSH Terms] OR "hiv"[All Fields] OR ("HIV-infected"[All Fields] AND "adult"[MeSH Terms]) OR ("HIV-infected"[All Fields] AND "patients"[MeSH Terms]) OR (("hiv seropositivity"[MeSH Terms] OR ("hiv"[All Fields] AND "seropositivity"[All Fields]) OR "hiv seropositivity"[All Fields] OR ("hiv"[All Fields] AND "positive"[All Fields]) OR "HIV-positive"[All Fields]) AND "adult"[MeSH Terms]) OR (("hiv seropositivity"[MeSH Terms] OR ("hiv"[All Fields] AND "seropositivity"[All Fields]) OR "hiv seropositivity"[All Fields] OR ("hiv"[All Fields] AND "positive"[All Fields]) OR "HIV-positive"[All Fields]) AND "patients"[MeSH Terms]) OR ((("people s"[All Fields] OR "peopled"[All Fields] OR "peopling"[All Fields] OR "persons"[MeSH Terms] OR "persons"[All Fields] OR "people"[All Fields] OR "peoples"[All Fields]) AND ("lived"[All Fields] OR "lives"[All Fields] OR "living"[All Fields] OR "livings"[All Fields])) AND "hiv"[MeSH Terms]) OR ((("person s"[All Fields] OR "personable"[All Fields] OR "personableness"[All Fields] OR "personal"[All Fields] OR "personalisation"[All Fields] OR "personalise"[All Fields] OR "personalised"[All Fields] OR "personalising"[All Fields] OR "personality"[MeSH Terms] OR "personality"[All Fields] OR "personalities"[All Fields] OR "personality s"[All Fields] OR "personalization"[All Fields] OR "personalize"[All Fields] OR "personalized"[All Fields] OR "personalizes"[All Fields] OR "personalizing"[All Fields] OR "personally"[All Fields] OR "personals"[All Fields] OR "persons"[MeSH Terms] OR "persons"[All Fields] OR "person"[All Fields]) AND ("lived"[All Fields] OR "lives"[All Fields] OR "living"[All Fields] OR "livings"[All Fields])) AND "hiv"[MeSH Terms])) AND ("metabolic syndrome"[MeSH Terms] OR (("cardiovascular system"[MeSH Terms] OR ("cardiovascular"[All Fields] AND "system"[All Fields]) OR "cardiovascular system"[All Fields] OR "cardiovascular"[All Fields] OR "cardiovasculars"[All Fields]) AND "syndrome"[MeSH Terms]) OR "heart disease risk factors"[MeSH Terms] OR ("diabetes mellitus"[MeSH Terms] OR ("diabetes mellitus"[MeSH Terms] OR "diabetes insipidus"[MeSH Terms]) OR "hyperglycemia"[MeSH Terms]) OR ("cardiovascular diseases"[MeSH Terms] OR "coronary artery disease"[MeSH Terms] OR ("myocardial ischemia"[MeSH Terms] OR "coronary artery disease"[MeSH Terms]) OR "heart diseases"[MeSH Terms] OR "stroke"[MeSH Terms] OR "cerebrovascular disorders"[MeSH Terms]) OR ("kidney diseases"[MeSH Terms] OR "renal insufficiency, chronic"[MeSH Terms] OR "kidney failure, chronic"[MeSH Terms] OR "kidney failure, chronic"[MeSH Terms] OR "kidney failure, chronic"[MeSH Terms] OR "kidney failure, chronic"[MeSH Terms] OR (("abnormal"[All Fields] OR "abnormalities"[MeSH Subheading] OR "abnormalities"[All Fields] OR "congenital abnormalities"[MeSH Terms] OR ("congenital"[All Fields] AND "abnormalities"[All Fields]) OR "congenital abnormalities"[All Fields] OR "abnormality"[All Fields] OR "abnormally"[All Fields] OR "abnormals"[All Fields] OR "abnormities"[All Fields] OR "abnormity"[All Fields]) AND "erbb receptors"[MeSH Terms]) OR (("reduce"[All Fields] OR "reduced"[All Fields] OR "reduces"[All Fields] OR "reducing"[All Fields]) AND "erbb receptors"[MeSH Terms]))) | 13,016 |
| 8 | #1 AND #2 | ("hiv"[MeSH Terms] OR "hiv"[All Fields] OR ("HIV-infected"[All Fields] AND "adult"[MeSH Terms]) OR ("HIV-infected"[All Fields] AND "patients"[MeSH Terms]) OR (("hiv seropositivity"[MeSH Terms] OR ("hiv"[All Fields] AND "seropositivity"[All Fields]) OR "hiv seropositivity"[All Fields] OR ("hiv"[All Fields] AND "positive"[All Fields]) OR "HIV-positive"[All Fields]) AND "adult"[MeSH Terms]) OR (("hiv seropositivity"[MeSH Terms] OR ("hiv"[All Fields] AND "seropositivity"[All Fields]) OR "hiv seropositivity"[All Fields] OR ("hiv"[All Fields] AND "positive"[All Fields]) OR "HIV-positive"[All Fields]) AND "patients"[MeSH Terms]) OR ((("people s"[All Fields] OR "peopled"[All Fields] OR "peopling"[All Fields] OR "persons"[MeSH Terms] OR "persons"[All Fields] OR "people"[All Fields] OR "peoples"[All Fields]) AND ("lived"[All Fields] OR "lives"[All Fields] OR "living"[All Fields] OR "livings"[All Fields])) AND "hiv"[MeSH Terms]) OR ((("person s"[All Fields] OR "personable"[All Fields] OR "personableness"[All Fields] OR "personal"[All Fields] OR "personalisation"[All Fields] OR "personalise"[All Fields] OR "personalised"[All Fields] OR "personalising"[All Fields] OR "personality"[MeSH Terms] OR "personality"[All Fields] OR "personalities"[All Fields] OR "personality s"[All Fields] OR "personalization"[All Fields] OR "personalize"[All Fields] OR "personalized"[All Fields] OR "personalizes"[All Fields] OR "personalizing"[All Fields] OR "personally"[All Fields] OR "personals"[All Fields] OR "persons"[MeSH Terms] OR "persons"[All Fields] OR "person"[All Fields]) AND ("lived"[All Fields] OR "lives"[All Fields] OR "living"[All Fields] OR "livings"[All Fields])) AND "hiv"[MeSH Terms])) AND ("art"[MeSH Terms] OR "art"[All Fields] OR ("arv"[Journal] OR "arv"[All Fields]) OR ("antiretroviral therapy, highly active"[MeSH Terms] OR ("antiretroviral"[All Fields] AND "therapy"[All Fields] AND "highly"[All Fields] AND "active"[All Fields]) OR "highly active antiretroviral therapy"[All Fields] OR "haart"[All Fields] OR "haarts"[All Fields]) OR (("anti retroviral agents"[Pharmacological Action] OR "anti retroviral agents"[MeSH Terms] OR ("anti retroviral"[All Fields] AND "agents"[All Fields]) OR "anti retroviral agents"[All Fields] OR "antiretroviral"[All Fields] OR "antiretrovirally"[All Fields] OR "antiretrovirals"[All Fields]) AND ("therapeutics"[MeSH Terms] OR "therapeutics"[All Fields] OR "therapies"[All Fields] OR "therapy"[MeSH Subheading] OR "therapy"[All Fields] OR "therapy s"[All Fields] OR "therapys"[All Fields])) OR (("therapy"[MeSH Subheading] OR "therapy"[All Fields] OR "treat"[All Fields] OR "therapeutics"[MeSH Terms] OR "therapeutics"[All Fields] OR "treating"[All Fields] OR "treated"[All Fields] OR "treats"[All Fields]) AND "art"[MeSH Terms]) OR (("therapy"[MeSH Subheading] OR "therapy"[All Fields] OR "treat"[All Fields] OR "therapeutics"[MeSH Terms] OR "therapeutics"[All Fields] OR "treating"[All Fields] OR "treated"[All Fields] OR "treats"[All Fields]) AND "antiretroviral therapy, highly active"[MeSH Terms]) OR ((("therapy"[MeSH Subheading] OR "therapy"[All Fields] OR "treat"[All Fields] OR "therapeutics"[MeSH Terms] OR "therapeutics"[All Fields] OR "treating"[All Fields] OR "treated"[All Fields] OR "treats"[All Fields]) AND ("anti retroviral agents"[Pharmacological Action] OR "anti retroviral agents"[MeSH Terms] OR ("anti retroviral"[All Fields] AND "agents"[All Fields]) OR "anti retroviral agents"[All Fields] OR "antiretroviral"[All Fields] OR "antiretrovirally"[All Fields] OR "antiretrovirals"[All Fields])) AND "therapeutics"[MeSH Terms]) OR ("art"[MeSH Terms]) OR ("antiretroviral therapy, highly active"[MeSH Terms]) OR (("anti retroviral agents"[Pharmacological Action] OR "anti retroviral agents"[MeSH Terms] OR ("anti retroviral"[All Fields] AND "agents"[All Fields]) OR "anti retroviral agents"[All Fields] OR "antiretroviral"[All Fields] OR "antiretrovirally"[All Fields] OR "antiretrovirals"[All Fields]) AND "therapeutics"[MeSH Terms]) OR ("untreated"[All Fields] AND "hiv"[MeSH Terms]) OR ("HIV-uninfected"[All Fields] AND "persons"[MeSH Terms]) OR ("HIV-negative"[All Fields] AND "persons"[MeSH Terms])) | 110,069 |
| 7 | #3 OR #4 OR #5 OR #6 | "metabolic syndrome"[MeSH Terms] OR (("cardiovascular system"[MeSH Terms] OR ("cardiovascular"[All Fields] AND "system"[All Fields]) OR "cardiovascular system"[All Fields] OR "cardiovascular"[All Fields] OR "cardiovasculars"[All Fields]) AND "syndrome"[MeSH Terms]) OR "heart disease risk factors"[MeSH Terms] OR ("diabetes mellitus"[MeSH Terms] OR ("diabetes mellitus"[MeSH Terms] OR "diabetes insipidus"[MeSH Terms]) OR "hyperglycemia"[MeSH Terms]) OR ("cardiovascular diseases"[MeSH Terms] OR "coronary artery disease"[MeSH Terms] OR ("myocardial ischemia"[MeSH Terms] OR "coronary artery disease"[MeSH Terms]) OR "heart diseases"[MeSH Terms] OR "stroke"[MeSH Terms] OR "cerebrovascular disorders"[MeSH Terms]) OR ("kidney diseases"[MeSH Terms] OR "renal insufficiency, chronic"[MeSH Terms] OR "kidney failure, chronic"[MeSH Terms] OR "kidney failure, chronic"[MeSH Terms] OR "kidney failure, chronic"[MeSH Terms] OR "kidney failure, chronic"[MeSH Terms] OR (("abnormal"[All Fields] OR "abnormalities"[MeSH Subheading] OR "abnormalities"[All Fields] OR "congenital abnormalities"[MeSH Terms] OR ("congenital"[All Fields] AND "abnormalities"[All Fields]) OR "congenital abnormalities"[All Fields] OR "abnormality"[All Fields] OR "abnormally"[All Fields] OR "abnormals"[All Fields] OR "abnormities"[All Fields] OR "abnormity"[All Fields]) AND "erbb receptors"[MeSH Terms]) OR (("reduce"[All Fields] OR "reduced"[All Fields] OR "reduces"[All Fields] OR "reducing"[All Fields]) AND "erbb receptors"[MeSH Terms])) | 3,430,276 |
| 6 | (((((((((kidney diseases[MeSH Terms]) OR (chronic kidney disease[MeSH Terms])) OR (CKD[MeSH Terms])) OR (chronic kidney failure[MeSH Terms])) OR (chronic renal failure[MeSH Terms])) OR (end stage renal disease[MeSH Terms])) OR (ESRD[MeSH Terms])) OR (abnormal eGFR[MeSH Terms])) OR (reduced eGFR[MeSH Terms])) OR (kidney dysfunction[MeSH Terms]) | "kidney diseases"[MeSH Terms] OR "renal insufficiency, chronic"[MeSH Terms] OR "kidney failure, chronic"[MeSH Terms] OR "kidney failure, chronic"[MeSH Terms] OR "kidney failure, chronic"[MeSH Terms] OR "kidney failure, chronic"[MeSH Terms] OR (("abnormal"[All Fields] OR "abnormalities"[MeSH Subheading] OR "abnormalities"[All Fields] OR "congenital abnormalities"[MeSH Terms] OR ("congenital"[All Fields] AND "abnormalities"[All Fields]) OR "congenital abnormalities"[All Fields] OR "abnormality"[All Fields] OR "abnormally"[All Fields] OR "abnormals"[All Fields] OR "abnormities"[All Fields] OR "abnormity"[All Fields]) AND "erbb receptors"[MeSH Terms]) OR (("reduce"[All Fields] OR "reduced"[All Fields] OR "reduces"[All Fields] OR "reducing"[All Fields]) AND "erbb receptors"[MeSH Terms]) | 563,902 |
| 5 | ((((((((((((cardiovascular disease[MeSH Terms]) OR (cardiovascular event[MeSH Terms])) OR (CVD[MeSH Terms])) OR (coronary artery disease[MeSH Terms])) OR (CAD[MeSH Terms])) OR (myocardial infraction[MeSH Terms])) OR (MI[MeSH Terms])) OR (ischemic heart disease[MeSH Terms])) OR (IHD[MeSH Terms])) OR (heart disease[MeSH Terms])) OR (stroke[MeSH Terms])) OR (cerebrovascular disease[MeSH Terms])) OR (cerebrovascular event[MeSH Terms]) | "cardiovascular diseases"[MeSH Terms] OR "coronary artery disease"[MeSH Terms] OR "myocardial ischemia"[MeSH Terms] OR "coronary artery disease"[MeSH Terms] OR "heart diseases"[MeSH Terms] OR "stroke"[MeSH Terms] OR "cerebrovascular disorders"[MeSH Terms] | 2,620,373 |
| 4 | (((diabetes mellitus[MeSH Terms]) OR (diabetes[MeSH Terms])) OR (DM[MeSH Terms])) OR (hyperglycemia[MeSH Terms]) | "diabetes mellitus"[MeSH Terms] OR "diabetes mellitus"[MeSH Terms] OR "diabetes insipidus"[MeSH Terms] OR "hyperglycemia"[MeSH Terms] | 505,355 |
| 3 | (((metabolic syndrome[MeSH Terms]) OR (metabolic complication[MeSH Terms])) OR (cardiovascular syndrome[MeSH Terms])) OR (cardiovascular risk factors[MeSH Terms]) | "metabolic syndrome"[MeSH Terms] OR (("cardiovascular system"[MeSH Terms] OR ("cardiovascular"[All Fields] AND "system"[All Fields]) OR "cardiovascular system"[All Fields] OR "cardiovascular"[All Fields] OR "cardiovasculars"[All Fields]) AND "syndrome"[MeSH Terms]) OR "heart disease risk factors"[MeSH Terms] | 56,647 |
| 2 | (((((((((((((((((ART) OR (ARV)) OR (HAART)) OR (antiretroviral therapy)) OR (treated with ART[MeSH Terms])) OR (treated with ARV[MeSH Terms])) OR (treated with HAART[MeSH Terms])) OR (treated with antiretroviral therapy[MeSH Terms])) OR (on ART[MeSH Terms])) OR (on ARV[MeSH Terms])) OR (on HAART[MeSH Terms])) OR (on antiretroviral therapy[MeSH Terms])) OR (untreated HIV[MeSH Terms])) OR (naïve[MeSH Terms])) OR (HIV-uninfected individuals[MeSH Terms])) OR (HIV-uninfected people[MeSH Terms])) OR (HIV-negative individuals[MeSH Terms])) OR (HIV-negative people[MeSH Terms]) | "art"[MeSH Terms] OR "art"[All Fields] OR ("arv"[Journal] OR "arv"[All Fields]) OR ("antiretroviral therapy, highly active"[MeSH Terms] OR ("antiretroviral"[All Fields] AND "therapy"[All Fields] AND "highly"[All Fields] AND "active"[All Fields]) OR "highly active antiretroviral therapy"[All Fields] OR "haart"[All Fields] OR "haarts"[All Fields]) OR (("anti retroviral agents"[Pharmacological Action] OR "anti retroviral agents"[MeSH Terms] OR ("anti retroviral"[All Fields] AND "agents"[All Fields]) OR "anti retroviral agents"[All Fields] OR "antiretroviral"[All Fields] OR "antiretrovirally"[All Fields] OR "antiretrovirals"[All Fields]) AND ("therapeutics"[MeSH Terms] OR "therapeutics"[All Fields] OR "therapies"[All Fields] OR "therapy"[MeSH Subheading] OR "therapy"[All Fields] OR "therapy s"[All Fields] OR "therapys"[All Fields])) OR (("therapy"[MeSH Subheading] OR "therapy"[All Fields] OR "treat"[All Fields] OR "therapeutics"[MeSH Terms] OR "therapeutics"[All Fields] OR "treating"[All Fields] OR "treated"[All Fields] OR "treats"[All Fields]) AND "art"[MeSH Terms]) OR (("therapy"[MeSH Subheading] OR "therapy"[All Fields] OR "treat"[All Fields] OR "therapeutics"[MeSH Terms] OR "therapeutics"[All Fields] OR "treating"[All Fields] OR "treated"[All Fields] OR "treats"[All Fields]) AND "antiretroviral therapy, highly active"[MeSH Terms]) OR ((("therapy"[MeSH Subheading] OR "therapy"[All Fields] OR "treat"[All Fields] OR "therapeutics"[MeSH Terms] OR "therapeutics"[All Fields] OR "treating"[All Fields] OR "treated"[All Fields] OR "treats"[All Fields]) AND ("anti retroviral agents"[Pharmacological Action] OR "anti retroviral agents"[MeSH Terms] OR ("anti retroviral"[All Fields] AND "agents"[All Fields]) OR "anti retroviral agents"[All Fields] OR "antiretroviral"[All Fields] OR "antiretrovirally"[All Fields] OR "antiretrovirals"[All Fields])) AND "therapeutics"[MeSH Terms]) OR ("art"[MeSH Terms]) OR ("antiretroviral therapy, highly active"[MeSH Terms]) OR (("anti retroviral agents"[Pharmacological Action] OR "anti retroviral agents"[MeSH Terms] OR ("anti retroviral"[All Fields] AND "agents"[All Fields]) OR "anti retroviral agents"[All Fields] OR "antiretroviral"[All Fields] OR "antiretrovirally"[All Fields] OR "antiretrovirals"[All Fields]) AND "therapeutics"[MeSH Terms]) OR ("untreated"[All Fields] AND "hiv"[MeSH Terms]) OR ("HIV-uninfected"[All Fields] AND "persons"[MeSH Terms]) OR ("HIV-negative"[All Fields] AND "persons"[MeSH Terms]) | 325,162 |
| 1 | ((((((((((HIV) OR (HIV-infected adults[MeSH Terms])) OR (HIV-infected individuals[MeSH Terms])) OR (HIV-infected patients[MeSH Terms])) OR (HIV-positive adults[MeSH Terms])) OR (HIV-positive individuals[MeSH Terms])) OR (HIV-positive patients[MeSH Terms])) OR (People living with HIV[MeSH Terms])) OR (PLWHIV[MeSH Terms])) OR (PLWHA[MeSH Terms])) OR (persons living with HIV[MeSH Terms]) | "hiv"[MeSH Terms] OR "hiv"[All Fields] OR ("HIV-infected"[All Fields] AND "adult"[MeSH Terms]) OR ("HIV-infected"[All Fields] AND "patients"[MeSH Terms]) OR (("hiv seropositivity"[MeSH Terms] OR ("hiv"[All Fields] AND "seropositivity"[All Fields]) OR "hiv seropositivity"[All Fields] OR ("hiv"[All Fields] AND "positive"[All Fields]) OR "HIV-positive"[All Fields]) AND "adult"[MeSH Terms]) OR (("hiv seropositivity"[MeSH Terms] OR ("hiv"[All Fields] AND "seropositivity"[All Fields]) OR "hiv seropositivity"[All Fields] OR ("hiv"[All Fields] AND "positive"[All Fields]) OR "HIV-positive"[All Fields]) AND "patients"[MeSH Terms]) OR ((("people s"[All Fields] OR "peopled"[All Fields] OR "peopling"[All Fields] OR "persons"[MeSH Terms] OR "persons"[All Fields] OR "people"[All Fields] OR "peoples"[All Fields]) AND ("lived"[All Fields] OR "lives"[All Fields] OR "living"[All Fields] OR "livings"[All Fields])) AND "hiv"[MeSH Terms]) OR ((("person s"[All Fields] OR "personable"[All Fields] OR "personableness"[All Fields] OR "personal"[All Fields] OR "personalisation"[All Fields] OR "personalise"[All Fields] OR "personalised"[All Fields] OR "personalising"[All Fields] OR "personality"[MeSH Terms] OR "personality"[All Fields] OR "personalities"[All Fields] OR "personality s"[All Fields] OR "personalization"[All Fields] OR "personalize"[All Fields] OR "personalized"[All Fields] OR "personalizes"[All Fields] OR "personalizing"[All Fields] OR "personally"[All Fields] OR "personals"[All Fields] OR "persons"[MeSH Terms] OR "persons"[All Fields] OR "person"[All Fields]) AND ("lived"[All Fields] OR "lives"[All Fields] OR "living"[All Fields] OR "livings"[All Fields])) AND "hiv"[MeSH Terms]) | 429,286 |

**Table S2.6 EBSCO searching.**

| **No** | **Search Terms** | **Results** |
| --- | --- | --- |
| 11 | SU S1 AND S2 AND S7 | **3,475** |
| 10 | SU S2 AND S7 | 11,413 |
| 9 | SU S1 AND S7 | 30,283 |
| 8 | SU S1 AND S2 | 121,623 |
| 7 | SU S3 OR S4 OR S5 OR S6 | 7,960,192 |
| 6 | SU “kidney diseases” OR “chronic kidney disease” OR “CKD” OR “chronic kidney failure” OR “chronic renal failure” OR “end stage renal disease” OR “ESRD” OR “abnormal eGFR” OR “reduced eGFR” OR “kidney dysfunction” | 609,332 |
| 5 | SU “cardiovascular disease” OR “cardiovascular event” OR “CVD” OR “coronary artery disease” OR “CAD” OR “myocardial infraction” OR “MI” OR “ischemic heart disease” OR “ihd” OR “heart disease” OR “stroke” OR “cerebrovascular disease” OR “cerebrovascular event” | 3,366,018 |
| 4 | SU “diabetes mellitus” OR “diabetes” OR “DM” OR “hyperglycemia” | 4,005,697 |
| 3 | SU “metabolic syndrome” OR “metabolic complication” OR “cardiovascular syndrome” OR “cardiovascular risk factors” | 277,042 |
| 2 | SU “ART” OR “ARV” OR “HAART” OR “antiretroviral therapy” OR “treated with ART” OR “treated with ARV” OR “treated with HAART” OR “treated with antiretroviral therapy” OR “on ART” OR “on ARV” OR “on HAART” OR “on antiretroviral therapy” OR “untreated HIV” OR “naïve” OR “HIV-uninfected individuals” OR “HIV-uninfected people” OR “HIV-negative individuals” OR “HIV-negative people” | 9,113,870 |
| 1 | SU "HIV" OR "HIV-infected adults" OR "HIV-infected individuals" OR "HIV-infected patients" OR "HIV-positive adults" OR "HIV-positive individuals" OR "HIV-positive patients" OR "People living with HIV" OR "PLWHIV" OR "PLWHA" OR "persons living with HIV" | 1,813,839 |

**Table S2.7 TCI searching.**

| **Search Terms** | **Result** |
| --- | --- |
| 1. MetS |  |
| HIV infected patients people living with HIV/AIDS AND metabolic syndrome | 7 |
| 2. DM |  |
| HIV infected patients people living with HIV/AIDS AND diabetes mellitus | 992 |
| 3. CVD |  |
| HIV infected patients people living with HIV/AIDS AND cardiovascular diseases | 107 |
| HIV infected patients people living with HIV/AIDS AND coronary artery disease | 194 |
| HIV infected patients people living with HIV/AIDS AND myocardial infarction | 89 |
| HIV infected patients people living with HIV/AIDS AND stroke | 884 |
| 4. CKD |  |
| HIV infected patients people living with HIV/AIDS AND chronic kidney disease | 265 |
| **Total** | **2,538** |

**Table S2.8 TJI searching.**

| **Search Terms** | **Result** |
| --- | --- |
| 1. MetS |  |
| HIV infected patients people living with HIV/AIDS AND metabolic syndrome | 100 |
| 2. DM |  |
| HIV infected patients people living with HIV/AIDS AND diabetes mellitus | 341 |
| 3. CVD |  |
| HIV infected patients people living with HIV/AIDS AND cardiovascular diseases | 139 |
| HIV infected patients people living with HIV/AIDS AND coronary artery disease | 196 |
| HIV infected patients people living with HIV/AIDS AND myocardial infarction | 107 |
| HIV infected patients people living with HIV/AIDS AND stroke | 143 |
| 4. CKD |  |
| HIV infected patients people living with HIV/AIDS AND chronic kidney disease | 137 |
| **Total** | **1,163** |

**Table S2.9 TDC searching.**

| **Search Terms** | **Result** |
| --- | --- |
| 1. MetS |  |
| HIV infected patients people living with HIV/AIDS AND metabolic syndrome | 40 |
| 2. DM |  |
| HIV infected patients people living with HIV/AIDS AND diabetes mellitus | 132 |
| 3. CVD |  |
| HIV infected patients people living with HIV/AIDS AND cardiovascular diseases | 94 |
| HIV infected patients people living with HIV/AIDS AND coronary artery disease | 30 |
| HIV infected patients people living with HIV/AIDS AND myocardial infarction | 5 |
| HIV infected patients people living with HIV/AIDS AND stroke | 127 |
| 4. CKD |  |
| HIV infected patients people living with HIV/AIDS AND chronic kidney disease | 6 |
| **Total** | **434** |

**Table S2.10 ThaiJO searching.**

| **Search terms** | **Result** |
| --- | --- |
| 1. MetS |  |
| HIV infected patients people living with HIV/AIDS AND metabolic syndrome | 1 |
| 2. DM |  |
| HIV infected patients people living with HIV/AIDS AND diabetes mellitus | 20 |
| 3. CVD |  |
| HIV infected patients people living with HIV/AIDS AND cardiovascular diseases | 14 |
| HIV infected patients people living with HIV/AIDS AND coronary artery disease | 0 |
| HIV infected patients people living with HIV/AIDS AND myocardial infarction | 0 |
| HIV infected patients people living with HIV/AIDS AND stroke | 2 |
| 4. CKD |  |
| HIV infected patients people living with HIV/AIDS AND chronic kidney disease | 14 |
| **Total** | **51** |
