# Supplementary material for: Burden of metabolic syndrome in the global adult HIV-infected population: a systematic review and meta-analysis
Source: BMC Public Health. 2024 Sep 28;24:2657. doi: 10.1186/s12889-024-20118-3 (PMC11438355; doi:10.1186/s12889-024-20118-3)
Supplement: Supplementary file 3 — Additional File 3 [file 12889_2024_20118_MOESM3_ESM.docx]

**Additional file 3**

**Table** **S3.1 Quality assessment of the included cross-sectional studies using the Newcastle-Ottawa scale.**

| **Question** | | |
| --- | --- | --- |
| **Selection: (Maximum 5 stars)** | | |
| 1 | **Representativeness of the sample** | |
|  | 1. Truly representative of the average in the target population.   (All subjects or random sampling) | ***** |
|  | 1. Somewhat representative of the average in the target population. (Non-random sampling) | ***** |
|  | 1. Selected group of users or convenience sample   e.g., volunteers, members, nurses |  |
|  | 1. No description of the sampling strategy |  |
| 2 | **Sample size** | |
|  | 1. Justified and satisfactory | ***** |
|  | 1. Not justified. |  |
| 3 | **Non-respondents** | |
|  | 1. Comparability between respondents and non-respondents characteristics is established, and the response rate is satisfactory. | * |
|  | 1. The response rate is unsatisfactory, or the comparability between respondents and non-respondents is unsatisfactory. |  |
|  | 1. No description of the response rate or the characteristics of the responders and the non-responders. |  |
| 4 | **Ascertainment of exposure (risk factor)** |  |
|  | 1. Secure record (medical charts) or validated measurement tool. | ** |
|  | 1. Non-validated measurement tool, but the tool is available or   described or Self report. | * |
|  | c) No description of the measurement tool. |  |
| **Comparability: (Maximum 2 stars)** | | |
| 1 | **The subjects in different outcome groups are comparable, based on the study design or analysis. Confounding factors are controlled.** |  |
|  | 1. The study controls for the most important factor, i.e., other factors potentially impairing cognitive outcome. | * |
|  | 1. The study control for any additional factor. | * |
| **Outcome: (Maximum 3 stars)** | | |
| 1 | **Assessment of outcome** |  |
|  | 1. Independent structured assessment or blind assessment | ** |
|  | 1. Record linkage | ** |
|  | 1. Self-report | * |
|  | 1. No description |  |
| 2 | **Statistical test** |  |
|  | 1. The statistical test used to analyze the data is clearly described and appropriate, and the measurement of the association is presented, including confidence intervals and the probability level (p-value). | * |
|  | 1. The statistical test is not appropriate, not described or incomplete. |  |

This scale has been adapted from the Newcastle-Ottawa scale created for cross-sectional studies by Herzog et al [1].

**Scores for cross-sectional studies**

Very good studies: 9-10 points

Good studies: 7-8 points

Satisfactory studies: 5-6 points

Unsatisfactory studies: 0-4 points

**Reference**

1. Herzog R, Álvarez-Pasquin M, Díaz C, Del Barrio JL, Estrada JM, Gil Á. Are healthcare workers’ intentions to vaccinate related to their knowledge, beliefs and attitudes? A systematic review. BMC public health. 2013;13(1):1-17.

**Table S3.2 Quality assessment of the included cohort studies using the Newcastle-Ottawa scale.**

| **Question** | | |
| --- | --- | --- |
| **Selection** | | |
| 1 | **Representativeness of the exposed cohort** | |
|  | 1. truly representative of the average in the target population in the community | ***** |
|  | 1. somewhat representative of the average in the target population in the community | ***** |
|  | 1. selected group of users (e.g., patients receiving ART, selection based on hospital admission) |  |
|  | 1. no description of the derivation of the cohort |  |
| 2 | **Selection of the non-exposed cohort (same source population)** | |
|  | 1. drawn from the same community as the exposed cohort | ***** |
|  | 1. drawn from a different source |  |
|  | 1. no description of the derivation of the non-exposed cohort |  |
| 3 | **Ascertainment of exposure** | |
|  | 1. secure record (e.g., surgical records) | ***** |
|  | 1. structured interview | ***** |
|  | 1. written self-report |  |
|  | 1. no description |  |
| 4 | **Demonstration that outcome of interest was not present at start of study** | |
|  | 1. yes | ***** |
|  | 1. no |  |
| **Comparability** | | |
| 1 | **Comparability of cohorts on the basis of the design or analysis** | |
|  | 1. study controls for _____________ (select the most important factor) | ***** |
|  | 1. study controls for any additional factor (These criteria could be modified to indicate specific control for a second important factor.) | ***** |
| **Outcome** | | |
| 1 | **Assessment of outcome** | |
|  | 1. independent blind assessment | ***** |
|  | 1. record linkage | ***** |
|  | 1. self-report |  |
|  | 1. no description |  |
| 2 | **Was follow-up long enough for outcomes to occur** | |
|  | 1. yes (select an adequate follow up period for outcome of interest) | ***** |
|  | 1. no |  |
| 3 | **Adequacy of follow-up of cohorts** | |
|  | 1. complete follow up - all subjects accounted for | ***** |
|  | 1. subjects lost to follow up unlikely to introduce bias - small number lost: <10% |  |
|  | 1. follow up rate <90% and no description of those lost |  |
|  | 1. no statement or insufficient information on missing data |  |

**Thresholds for converting the Newcastle-Ottawa scales to the Agency for Healthcare Research and Quality (AHRQ) standards (good, fair, and poor):**

**Good quality:** > 3 stars in the selection domain AND 1–2 stars in the comparability domain AND 2–3 stars in the outcome/exposure domain.

**Fair quality:** 2 stars in the selection domain AND 1–2 stars in the comparability domain AND 2–3 stars in the outcome/exposure domain.

**Poor quality:** 0-1 star in selection domain OR 0 star in the comparability domain OR 0-1 star in the outcome/exposure domain.

**Table S3.3 Quality assessment of the included case-control study using the Newcastle-Ottawa scale.**

| **Question** | | |
| --- | --- | --- |
| **Selection** | | |
| 1 | **Is the case definition adequate?** | |
|  | 1. yes, with independent validation | ***** |
|  | 1. yes, e.g., record linkage or based on self-reports |  |
|  | 1. no description |  |
| 2 | **Representativeness of the cases** | |
|  | 1. consecutive or obviously representative series of cases | ***** |
|  | 1. potential for selection biases or not stated |  |
| 3 | **Selection of Controls** | |
|  | 1. controls from same community (same source population) | ***** |
|  | 1. hospital controls or different source |  |
|  | 1. no description |  |
| 4 | **Definition of Controls** | |
|  | 1. no history of diseases (endpoint) | ***** |
|  | 1. no description of sources |  |
| **Comparability** | | |
| 1 | **Comparability of cases and controls on the basis of the design or analysis** | |
|  | 1. study controls for (Select the most important factor.) | ***** |
|  | 1. study controls for any additional factor (These criteria could be modified to indicate specific control for a second important factor.) | ***** |
| **Exposure** | | |
| 1 | **Ascertainment of exposure** | |
|  | 1. secure record (e.g., hospital records, registry data) | ***** |
|  | 1. structured interview where blind to case/control status | ***** |
|  | 1. interview not blinded to case-control status |  |
|  | 1. written self-report or medical record only |  |
|  | 1. no description |  |
| 2 | **Same method of ascertainment for cases and controls** | |
|  | 1. yes | ***** |
|  | 1. no |  |
| 3 | **Non-Response rate** | |
|  | 1. same rate for both groups | ***** |
|  | 1. non respondents described |  |
|  | 1. rate different and no designation |  |

**Thresholds for converting the Newcastle-Ottawa scales to the Agency for Healthcare Research and Quality (AHRQ) standards (good, fair, and poor):**

**Good quality:** > 3 stars in the selection domain AND 1–2 stars in the comparability domain AND 2–3 stars in the outcome/exposure domain.

**Fair quality:** 2 stars in the selection domain AND 1–2 stars in the comparability domain AND 2–3 stars in the outcome/exposure domain.

**Poor quality:** 0-1 star in selection domain OR 0 star in the comparability domain OR 0-1 star in the outcome/exposure domain.
