# Supplementary material for: Burden of metabolic syndrome in the global adult HIV-infected population: a systematic review and meta-analysis
Source: BMC Public Health. 2024 Sep 28;24:2657. doi: 10.1186/s12889-024-20118-3 (PMC11438355; doi:10.1186/s12889-024-20118-3)
Supplement: Supplementary file 5 — Additional File 5 [file 12889_2024_20118_MOESM5_ESM.docx]

**Additional file 5**

**Table S5 Characteristics of the studies included in the review.**

| **ID** | **Author** | **Year*** | **Study design** | **Country** | **Study size** | | | **Age of HIV-infected patients (years)** | | | | **Gender (%)** | | **Duration of HIV infection (years)** | | | | | | | **Quality** |
| --- | --- | --- | --- | --- | --- | --- | --- | --- | --- | --- | --- | --- | --- | --- | --- | --- | --- | --- | --- | --- | --- |
|  |  |  |  |  | **All** | **ART*** | **non-ART*** | **Mean** | **SD** | **Median** | **IQR** | **M*** | **F*** | **Mean** | | **SD** | **Median** | | | **IQR** |  |
| **African Region** | | | | | | | | | | | | | | | | | | | | | |
| 1 | Zannou DM, et al [1] | 2009 | Prospective cohort study | Benin | 88 | 88 | - | 38 | 9.7 | - | - | 40.5 | 59.5 | - | | - | - | | - | | Poor |
| 2 | Awotedu K,  et al [2] | 2010 | Cross-sectional study | South Africa | 191 | 85 | 106 | 37.7 | 9.2 | - | - | 12.8 | 87.2 | - | | - | - | | - | | Good |
| 3 | Berhane T,  et al [3] | 2012 | Cross-sectional study | Ethiopia | 313 | 313 | - | Age 18-20 years=33.9%;  30-39 years=40.9%;  >40 years=25.2% | | | | 34.8 | 65.2 | - | | - | - | | - | | Good |
| 4 | Tesfaye DY, et al [4] | 2014 | Cross-sectional study | Ethiopia | 374 | 188 | 186 | 32.7 | 9.7 | - | - | 31.2 | 68.8 | - | | - | - | | - | | Good |
| 5 | Mbunkah HA, et al [5] | 2014 | Cross-sectional study | Cameroon | 173 | 112 | 61 | 1st Line ART group: mean(sd)=41.1(11.2);  2nd Line ART group: mean(sd)=38.7(11.3) years | | | | 29.5 | 70.5 | Patients with MetS mean(sd)=3.6(1.1); without MetS mean(sd)=2.7(0.8) years | | | | | | | Fair |
| 6 | Uwanuruochi VN, et al [6] | 2015 | Cross-sectional study | Nigeria | 165 | 105 | 60 | 41.35 | 10.41 | - | - | 45.5 | 54.5 | - | | - | - | | - | | Fair |
| 7 | Longo-Mbenza B,  et al [7] | 2015 | Cross-sectional study | Congo | 116 | - | - | 42 | 9 | - | - | 46.6 | 53.4 | - | | - | - | | - | | Fair |
| 8 | Guira O, et al [8] | 2016 | Cross-sectional study | Burkina Faso | 300 | 300 | - | 44.8 | 7.4 | - | - | 31 | 69 | - | | - | - | | - | | Fair |
| 9 | Sobieszczyk ME, et al [9] | 2016 | Cohort study | South Africa | 160 | - | 160 | - | - | 24 | 21-28 | 0 | 100 | Time after seroconversion:  median(iqr)= 42 (28–62) days | | | | | | | Good |
| 10 | Obirikorang C, et al [10] | 2016 | Cross-sectional study | Ghana | 433 | 294 | 139 | 40.3 | 0.8 | - | - | 41.3 | 58.7 | Duration<1 years=41.1%;  1-5 years=46.7%;  >5 years=12.2% | | | | | | | Fair |
| 11 | Nguyen KA, et al [11] | 2017 | Cross-sectional study | South Africa | 748 | 653 | 46 | - | - | 38 | 32-44 | 21 | 79 | - | | - | 5 | | 2-9 | | Fair |
| 12 | Muhammad FY, et al [12] | 2017 | Cross-sectional study | Nigeria | 300 | 150 | 150 | 34.8 | 9.9 | - | - | 36 | 64 | - Patients on ART group:  Patients with MetS mean(sd)=7.4(4.3); without MetS mean(sd)=4.3(2.7) years  - Naïve patients’ group:  Patients with MetS mean(sd)=3.4(1.4); without MetS mean(sd)=1.7(2.7) years | | | | | | | Good |
| 13 | Labhardt ND, et al [13] | 2017 | Cross-sectional study | South Africa | 1166 | 1166 | - | - | - | 44.4 | 35.3-54.4 | 65.8 | 34.2 | - | | - | - | | - | | Good |
| 14 | Ikama M, et al [14] | 2018 | Cross-sectional study | Congo | 135 | 64 | 71 | 42.6 | 2.9 | - | - | 61.5 | 38.5 | - | | - | - | | - | | Fair |
| 15 | Osoti A, et al [15] | 2018 | Cross-sectional study | Kenya | 300 | 164 | 136 | 43.1 | 9.4 | - | - | 42.7 | 57.3 | Patients on ART mean(sd)=6.6(4.7);  Naïve patients mean(sd)=3.7(4.4) years | | | | | | | Good |
| 16 | Kiama CN,  et al [16] | 2018 | Cross-sectional study | Kenya | 360 | - | - | Age 18-24 years=3.1%;  25-34 years=25%;  35-44 years=35%;  45-54 years=28.1%;  >55 years=9.9% | | | | 30.1 | 66.9 | - | | - | - | | - | | Good |
| 17 | Bosho DD,  et al [17] | 2018 | Cross-sectional study | Ethiopia | 268 | - | - | 39.32 | 10.63 | - | - | 21.3 | 78.7 | Duration<6 years=45.5%;  ≥6 years=54.5% | | | | | | | Good |
| 18 | Katoto PD,  et al [18] | 2018 | Cross-sectional study | Congo | 495 | 474 | 21 | - | - | 43 | 36-51 | 28 | 72 | - | | - | - | | - | | Good |
| 19 | Phalane E,  et al [19] | 2018 | Cross-sectional analysis from multinational longitudinal study | South Africa | 114 | 85 | 25 | 53.4 | 5.6 | - | - | 20.2 | 79.8 | - | | - | - | | - | | Good |
| 20 ^a^ | Bune GT, et al [20] | 2019 | Cross-sectional study | Ethiopia | 633 | 422 | 211 | 36.4 | 8.7 | - | - | 40.6 | 59.4 | - | | - | - | | - | | Fair |
| 21 ^a^ | Bune GT, et al [21] | 2020 | Case-control study | Ethiopia | 633 | 422 | 211 | Age ≤34 years=50%; 35-44 years=32%; ≥45 years=18% | | | | 37.8 | 62.2 | Duration<1 years=14%; 1-4 years=35%; 5-9 years=10% | | | | | | | Good |
| 22 | Møller SP,  et al [22] | 2020 | Cross-sectional study | Ethiopia | 329 | - | 329 | 33 | - | - | - | 32.8 | 67.2 | - | | - | - | | - | | Good |
| 23 | Gebrie A, et al [23] | 2020 | Cross-sectional study | Ethiopia | 407 | 407 | - | 38.6 | 10.3 | - | - | 39.6 | 60.4 | Duration<5 years=18.9%;  5-10 years=43.7%;  >10 years=37.3% | | | | | | | Good |
| 24 | Masyuko SJ, et al [24] | 2020 | Cross-sectional study | Kenya | 300 | 300 | - |  |  | 45 | 39.5-53 | 50 | 50 | - | | - | - | | - | | Good |
| 25 | Hamooya BM, et al [25] | 2021 | Cross-sectional study | Zambia | 1108 | 1108 | - | - | - | 41 | 34-49 | 39.9 | 60.1 | - | | - | - | | - | | Good |
| 26 | Hanley S, et al [26] | 2021 | Cross-sectional study | South Africa | 372 | 372 | - | 33.5 | 6.1 | - | - | 0 | 100 | - | | - | - | | - | | Fair |
| 27 | Woldu M, et al [27] | 2022 | Cross-sectional analysis from cohort study | Ethiopia | 288 | 288 | - | 43.5 | 11.3 | - | - | 43.8 | 56.3 | - | | - | - | | - | | Good |
| 28 | Ojong E, et al [28] | 2022 | Cross-sectional study | Nigeria | 150 | 75 | 75 | Patients on ART mean(sd)=39.96(9.12);  Naïve patients mean(sd)=37.84(9.54) years | | | | 48.9 | 51.1 | - | | - | - | | - | | Good |
| 29 | Woldeyes E, et al [29] | 2022 | Cross-sectional study | Ethiopia | 333 | 333 | - | 45 | 10.4 | - | - | 30.9 | 69.1 | 10.58 | | 3.9 | - | | - | | Good |
| 30 | Kiyimba T, et al [30] | 2022 | Cross-sectional study | Uganda | 254 | 254 | - | 41.7 | 10.7 | - | - | 28.7 | 71.3 | 9.6 | | 7.3 | - | | - | | Good |
| 31 | Malindisa E, et al [31] | 2023 | Cross-sectional study | Tanzania | 223 | 223 | - | 44 | 12 | - | - | 20.2 | 79.8 | Duration<10 years=41.8%; ≥10 years=58.2% | | | | | | | Good |
| 32 | Jumare J,et al [32] | 2023 | Cross-sectional analysis of baseline data from a prospective cohort study | Nigeria | 440 | 440 | - | - | - | 45 | 36-52 | 49.5 | 50.5 | - | | - | - | | - | | Good |
| 33 | Abdela AA, et al [33] | 2023 | A multicenter cross-sectional study | Ethiopia | 518 | 518 | - | 45 | 10.7 | - | - | 32.8 | 67 | 10.8 | | 3.85 | - | | - | | Good |
| 34 | Amutuhaire W, et al [34] | 2023 | Cross-sectional analysis of baseline data from a prospective cohort study | Uganda | 309 | - | 309 | - | - | 31 | 27-39 | 40.8 | 59.2 | - | | - | - | | - | | Good |
| **Region of the Americas** | | | | | | | | | | | | | | | | | | | | | |
| 35 | Johnsen S, et al [35] | 2006 | Cross-sectional study | US | 97 | 79 | 18 | - | - | 41 | 36-46 | 0 | 100 | - | | - | 8 | | 4.6-11.9 | | Fair |
| 36 | Salyer J, et al [36] | 2006 | Cross-sectional study | US | 95 | 95 | - | 41.3 | 8.4 | - | - | 83 | 17 | 7.7 | | 4.31 | - | | - | | Poor |
| 37 | Jacobson DL, et a [37] | 2006 | Cross-sectional analysis at baseline visit and Cohort study for HIV-infected group | US | 477 | 342 | 135 | 42 | 7 | - | - | 73 | 27 | - | | - | - | | - | | Good |
| 38 | Mondy K, et al [38] | 2007 | Prospective, cross-sectional study | US | 472 | 343 | - | 40.2 | 0 | - | - | 64.5 | 34.5 | Patients with MetS, mean(sd)=8.6(0.5); without MetS, mean(sd)=7.4(0.3) years | | | | | | | Good |
| 39 | Adeyemi O,  et al [39] | 2008 | Cross-sectional study | US | 111 | - | - | - | - | 54 | 50-66 | 79 | 21 | 12 | | 5.4 | - | | - | | Fair |
| 40 | Sobieszczyk ME, et al [40] | 2008 | Cross-sectional study | US | 1725 | - | - | 40 | 8.7 | - | - | 0 | 100 | - | | - | - | | - | | Good |
| 41 | Ances BM,  et al [41] | 2009 | Cross-sectional study | US | 130 | 130 | - | Age range 20-60 years | | | | 85 | 15 | - | | - | - | | - | | Good |
| 42 | Leite L, et al [42] | 2010 | Cross-sectional study | Brazil | 100 | - | - | 41.78 | 9.86 | - | - | 63 | 37 | 6 | | 3.38 | - | | - | | Fair |
| 43 | Pullinger CR, et al [43] | 2010 | Cross-sectional study | US | 267 | 191 | 76 | 45.3 | 8.3 | - | - | 26.7 | 73.3 | 12.1 | | 6.9 | - | | - | | Fair |
| 44 | Ramírez-Marrero FA, et al [44] | 2010 | Cross-sectional study | US | 897 | - | - | 44.7 | 10.1 | - | - | 64 | 36 | - | | - | - | | - | | Fair |
| 45 | de Carvalho EH, et al [45] | 2010 | Cross-sectional study | Brazil | 256 | - | - | 41 | 9.2 | - | - | 62.1 | 37.9 | 5.54 | | 3.78 | - | | - | | Fair |
| 46 | Alencastro PR, et al [46] | 2011 | Cross-sectional study | Brazil | 1227 | 811 | 416 | 38.6 | 10.1 | - | - | 50.6 | 49.4 | 4.9 | | 4.2 | - | | - | | Good |
| 47 | Lauda LG,  et al [47] | 2011 | Cross-sectional study | Brazil | 249 | - | - | Male: mean(sd) =42.03(9.56); Female: mean(sd)=40.12(9.75) years | | | | 52.2 | 47.8 | - | | - | - | | - | | Fair |
| 48 | Tiozzo E, et al [48] | 2015 | Cross-sectional study | US | 89 | 89 | - | 48 | 7 | - | - | 47 | 53 | 14 | | 12 | - | | - | | Fair |
| 49 | Raposo MA, et al [49] | 2017 | Cross-sectional study | Brazil | 87 | - | 87 | 36.57 | 9.38 | - | - | 75.9 | 24.1 | - | | - | - | | - | | Fair |
| 50 | Akl LD, et al [50] | 2017 | Cross-sectional study | Brazil | 273 | - | - | 47.7 | 5.8 | 44.4 | 35.3-54.4 | 34.2 | 65.8 | 9.9 | | 5.4 | - | | - | | Good |
| 51 | Cibrián-Ponce A, et al [51] | 2018 | Prospective cohort study | Mexico | 460 | 460 | - | - | - | 43 | 34-52 | 79.3 | 20.7 | - | | - | - | | - | | Poor |
| 52 | Sears S, et al [52] | 2019 | Cross-sectional study | US | 1861 | 1771 | 43 | Age 18-39 years =26.2%;  40-49 years=28%;  50-59 years=31.3%;  ≥60 years=14.5% | | | | 62 | 38 | Duration<5 years=23%;  5-9 years=22%;  ≥10 years=55% | | | | | | | Good |
| 53 | Yu B, et al [53] | 2019 | Cross-sectional study | US | 109 | - | - | 50.5 | 8.5 | - | - | 83.5 | 16.5 | - | | - | 18.4 | | 9-25.1 | | Fair |
| 54 | Tagliari CFdS, et al [54] | 2020 | Cross-sectional study | Brazil | 832 | 832 | - | 43.3 | 10 | - | - | 55.3 | 44.7 | - | | - | - | | - | | Fair |
| 55 | Kileel EM,  et al [55] | 2021 | Cross-sectional analysis from cohort study | 100 clinical sites across 12 countries (High Income and Latin America/Caribbean) | 4500 | 4500 | - | - | - | 51 | 46-55 | 77 | 23 | - | | - | - | | - | | Good |
| **European Region** | | | | | | | | | | | | | | | | | | | | | |
| 56 | Jericó C, et al [56] | 2005 | Cross-sectional study | Spain | 710 | 626 | 84 | 41.9 | 9.2 | - | - | 72 | 28 | - | | - | 9.4 | | 6.2-13.1 | | Good |
| 57 | Magny Bergersen B, et al [57] | 2006 | Cross-sectional study | Norway | 263 | 207 | 56 | 43.4 | 9.4 | - | - | 81.6 | 18.4 | Patients on ART: median(iqr)=7.1(3.7-11.5);  Naive patients: median(iqr)=3.1(0.5-7.9) years | | | | | | | Good |
| 58 | Estrada V,  et al [58] | 2006 | Cross-sectional study | Spain | 146 | 146 | - | 40.6 | 8 | - | - | 65.8 | 34.2 | - | | - | - | | - | | Good |
| 59 | Bonfanti P,  et al [59] | 2007 | Cross-sectional study | Italy | 1239 | 1053 | 186 | 43.2 | 9.2 | - | - | 71.8 | 28.2 | - | | - | - | | - | | Good |
| 60 | Palacios R,  et al [60] | 2007 | Prospective, observational study | Spain | 60 | 60 | - | - | - | 40.9 | 35.2-47.1 | 83.3 | 16.7 | - | | - | 3.2 | | 0.09-4.96 | | Poor |
| 61 | Badiou S, et al [61] | 2008 | Cross-sectional study | France | 232 | - | - | 41 | 9 | - | - | 75 | 25 | - | | - | - | | - | | Fair |
| 62 | de Saint Martin L, et al [62] | 2008 | Cross-sectional study | France | 161 | - | - | Patients with MetS mean(sd)=50(2.23); without MetS mean(sd)=41(0.68) years | | | | 72.1 | 27.9 | Patients with MetS mean(sd)=11.9(0.93); without MetS mean(sd)=10.1(0.44) years | | | | | | | Fair |
| 63 ^b^ | Squillace N,  et al [63] | 2009 | Cross-sectional study | Italy | 1324 | 1324 | - | 45 | 7 | - | - | 63 | 37 | Patients with MetS mean(sd)=14.11(5.18);  without MetS mean(sd)=14.36(5.37) years | | | | | | | Fair |
| 64 ^b^ | Guaraldi G,  et al [64] | 2009 | Cross-sectional study | Italy | 567 | 567 | - | 45 | 7 | - | - | 62 | 38 | 9.75 | | 5.25 | - | | - | | Fair |
| 65 | Young J, et al [65] | 2009 | Multi-center Cohort study | Switzerland | 1218 | 1218 | - | Patients with MetS median=39; without MetS median=37 years | | | | 68.1 | 31.9 | Patients with MetS median=0.6;  without MetS median=1.17 years | | | | | | | Good |
| 66 ^c^ | De Socio G,  et al [66] | 2009 | Nested cross-sectional study | Italy | 72 | - | 72 | 41.6 | 1.4 | - | - | 79.2 | 20.8 | 3 | | 0.6 | - | | - | | Good |
| 67 ^c^ | Bonfanti P,  et al [67] | 2010 | Cross-sectional study | Italy | 292 | - | 292 | - | - | 37 | 31-45 | 75 | 25 | - | | - | - | | - | | Good |
| 68 ^c^ | Bonfanti P,  et al [68] | 2012 | Prospective study | Italy | 235 | 129 | 59 | - | - | 38 | 32-47 | 75.5 | 24.5 | - | | - | - | | - | | Good |
| 69 | Hansen B.R, et al [69] | 2009 | Cross-sectional study | Denmark | 566 | 490 | 76 | - | - | 44.1 | 38.6-52.4 | 81.4 | 18.6 | - | | - | 9.5 | | 5-14.2 | | Good |
| 70 | Elgalib A, et al [70] | 2011 | Cross-sectional study | UK | 678 | 501 | 177 | 39.5 | 8.8 | - | - | 74 | 26 | Duration range 0 - 41.83 years | | | | | | | Good |
| 71 | Biron A, et al [71] | 2012 | Multi-center Cohort study | France | 269 | 269 | - | Patients with MetS mean(sd)=49(12); without MetS mean(sd)=41(10) years | | | | 66.9 | 33.1 | Patients with MetS mean(sd)=5.1(4.3); without MetS mean(sd)=4.7(3.9) years | | | | | | | Good |
| 72 | Pirro M, et al [72] | 2016 | Cross-sectional study | Italy | 170 | 170 | - | 51 | 11 | - | - | 80 | 20 | - | | - | - | | - | | Fair |
| 73 | Calza L, et al [73] | 2017 | Cross-sectional study | Italy | 586 | 488 | 98 | 45.2 | 17.1 | - | - | 81.7 | 18.3 | 8.3 | | 2.4 | - | | - | | Good |
| 74 | Arrive E, et al [74] | 2018 | Cross-sectional study | France | 268 | - | - | Age range 18-30 years | | | | 47 | 53 | - | | - | - | | - | | Good |
| 75 | Rogalska-Płońska M,  et al [75] | 2018 | Cross-sectional study | Poland | 270 | - | - | - | - | 37 | 21-27 | 71.5 | 28.5 | - | | - | 10 | | 2-27 | | Fair |
| 76 | Duro M, et al [76] | 2018 | Restospective study | Portugal | 266 | 180 | 69 | - | - | 42 | 35.3–50.8 | 69.5 | 30.5 | - | | - | 7 | | 3-12 | | Good |
| 77 | Pommier J-D, et al [77] | 2019 | Case-control study | France | 292 | - | - | Case group: median(iqr)=39(34–44); Control group: median(iqr)=41 (36–47) years | | | | 100 | 0 | Case group: median(iqr)=11.3 (5.3–10.1);  Control group: median(iqr)=7.9 (3.1–12.4) years | | | | | | | Good |
| 78 | Guaraldi G,  et al [78] | 2019 | Cross-sectional study | Italy | 665 | 665 | - | 53.11 | 7.98 | - | - | 81.05 | 18.95 | - | | - | 21.8 | | 15.3-26.9 | | Fair |
| 79 | Taramasso L, et al [79] | 2021 | Multicenter, nationwide cohort study | Italy | 3014 | - | - | SiMone study mean(sd)=43.2(9.2);  HIV-HY study mean(sd)=50.3(9.4);  STOPHIV study mean(sd)=48.7(10.6) years | | | | 73.3 | 26.7 | - | | - | - | | - | | Good |
| 80 | Mazzitelli M, et al [80] | 2022 | Cross-sectional study | Italy | 356 | 356 | - | 49 | 12 | - | - | 68.5 | 31.5 | Patients with MetS mean(sd)=15.9(0.6); without MetS mean(sd)=14.2(0.6) years | | | | | | | Good |
| 81 ^d^ | Borjesson RP, et al [81] | 2023 | Cohort study | Italy | 1564 | 1564 | - | - | - | 51.2 | 43.3-55.7 | 76.5 | 24.5 | - | - | | | 16.1 | | 8.8-26.2 | Good |
| **South-East Asian and Western Pacific regions** | | | | | | | | | | | | | | | | | | | | | |
| 82 | Samaras K,  et al [82] | 2007 | Cross-sectional study | Australia | 788 | 788 | - | - By IDF criteria:  Patients with MetS mean(sd)=45(10); without MetS mean(sd)=41(9) years;  - By ATP III criteria:  Patients with MetS mean(sd)=46(9); without MetS mean(sd)=41(9) years | | | | 84 | 16 |  | |  |  | |  | | Fair |
| 83 | Wu P-Y, et al [83] | 2012 | Cross-sectional study | Taiwan | 803 | 803 | - | Patients with MetS mean(sd)=44.5 (9.7); without MetS mean(sd)=36.8 (10.6) years | | | | 94.9 | 5.1 | - | | - | - | | - | | Good |
| 84 | Jeong SJ,  et al [84] | 2012 | Cross-sectional study | South Korea | 98 | 98 | - | 40.3 | 10.6 | - | - | 94.9 | 5.1 | - | | - | 2.25 | | 1.08-4.36 | | Fair |
| 85 | Bajaj S, et al [85] | 2013 | Cross-sectional study | India | 70 | 47 | 23 | 82.9% of patients aged between 30-45 years | | | | 71.4 | 28.6 | - | | - | - | | - | | Fair |
| 86 | Jantarapakde J, et al [86] | 2014 | Cross-sectional study | Thailand | 580 | 410 | 170 | - | - | 37 | 32–43 | 46.2 | 53.8 | - | | - | 5 | | 2-9 | | Good |
| 87 | Price J, et al [87] | 2015 | Cross-sectional analysis from cohort study | Australia | 144 | 144 | - | 42.1 | 8.8 | - | - | 100 | 0 | - | | - | 6.6 | | 2.75-12 | | Good |
| 88 | Kolgiri V, et al [88] | 2017 | Case-control study | India | 300 | 200 | 100 | Age range 20-60 years  - 1st line ART patients:  Age 20-40 years group mean(sd)=34.3(5.2) years;  Age 40-60 years group mean(sd)=48.3(4.8) years  - 2nd line ART patients:  Age 20-40 years group mean(sd)=35.6(4.3) years;  Age 40-60 years group mean(sd)=47.5(5.3) years | | | | 70.3 | 29.7 | - 1st line ART patients:  Age 20-40 years group mean=5.67 years;  Age 40-60 years group mean=6.7 years  - 2nd line ART patients  Age 20-40 years group mean=4.48 years;  Age 40-60 years group mean=6.54 years | | | | | | | Poor |
| 89 | Teekawong C, et al [89] | 2017 | Cross-sectional study | Thailand | 252 | 252 | - | 41.27 | 9.49 | - | - | 53.2 | 46.8 | - | | - | - | | - | | Good |
| 90 | Thongpool P, et al [90] | 2017 | Retrospective study | Thailand | 266 | 266 | - | 44.7 | 7.9 | - | - | 62.8 | 37.2 | - | | - | - | | - | | Good |
| 91 | Khaokaew P, et al [91] | 2018 | Cross-sectional study | Thailand | 424 | 424 | - | 43.04 | 9.88 | - | - | 50 | 50 | 8.13 | | 5.22 | Duration range 0.5-27.42 years | | | | Good |
| 92 | Zhao D, et al [92] | 2019 | Cross-sectional study | China | 296 | 264 | 32 | Age 40-49 years =52.7%;  50-59 years=25.7%;  ≥60 years=21.6% | | | | 77.4 | 22.6 | - | | - | - | | - | | Good |
| 93 | Shi R, et al [93] | 2020 | Cross-sectional analysis from cohort study | China | 2227 | 2227 | - | 44.1 | 14 | - | - | 78 | 22 | - | | - | 2.3 | | 0.6-5.1 | | Good |
| 94 | Mallya SD,  et al [94] | 2020 | Cross-sectional study | India | 182 | 123 | 59 | Age ≤45 years=43.4%;  >45 years=56.6% | | | | 63 | 37 | Duration≤3 years=54%;  >3 years=46% | | | | | | | Fair |
| 95 | Aurpibul L,  et al [95] | 2020 | Cross-sectional study | Thailand | 120 | 120 | - | 20.3 | 2.6 | - | - | 52 | 48 | - | | - | - | | - | | Fair |
| 96 | Sashindran V, et al [96] | 2021 | Cross-sectional study | India | 1208 | 1208 | - | 40.6 | 9.26 | - | - | 65.6 | 34.4 | - | | - | - | | - | | Good |
| 97 | Ang LW, et al [97] | 2021 | Retrospective study | Singapore | 2231 | 2231 | - | - | - | 41 | 32–50 | 93.9 | 6.1 | - | | - | 6.8 | | 4.1-9.6 | | Fair |
| 98 | Lu W-L, et al [98] | 2021 | Prospective cross-sectional study | Taiwan | 200 | 155 | 45 | 32.9 | 8.2 | - | - | 100 | 0 | - | | - | - | | - | | Fair |
| 99 | Han WM, et al [99] | 2022 | Cohort study | 12 countries in Asia and Australia | 4931 | 4931 | - | - | - | 34 | 29-41 | 66 | 34 | - | | - | - | | - | | Good |
| 100 | Haridas RS, et al [100] | 2022 | Single-center, prospective, observational study | India | 500 | 392 | 108 | 37 | 9.1 | - | - | 62.2 | 37.8 | Duration 12-24 months  =46.2%;  25-48 months=25.4%;  >48 months=28.4% | | | | | | | Poor |
| 101 | Shidhaye P, et al [101] | 2023 | Cross-sectional study | India | 440 | 440 | - | - Patients on ART>6 months mean(sd)=43.4(8.9), median(iqr)=43(38-49) years;  - Patients on ART≤6 month mean(sd)=38.6(9.8), median(iqr)=37(32-44) years | | | | 45 | 55 | Duration≤5 years=16.4%;  >5 years=83.6% | | | | | | | Good |
| **The international** | | | | | | | | | | | | | | | | | | | | | |
| 102 | Worm SW, et al [102] | 2010 | Prospective, observational study (cross-sectional analysis) | 11 cohorts in Europe, Australia and the US | 33347 | 33347 | - | - | - | 38 | 33-45 | 74 | 26 | - | | - | - | | - | | Fair |

* Year, Publication year; ART, patients treated with antiretroviral therapy; non-ART, untreated patients; M, male; F, female

^a^ Studies ID 20 and 21 used data from two-hospitals and health center in Gedio zone, southern Ethiopia. ID 20 is a cross-sectional study; ID 21 is a case-control study; ID 20 was selected to estimate the MetS prevalence.

^b^ Studies ID 63 and 64 used data from the University of Modena and Reggio Emilia, Italy. ID 64, a subset of ID 63, concentrated on patients receiving ART for longer than six months. ID 63 was selected to estimate the MetS prevalence.

^c^ Studies ID 66, 67 and 68 used data from the HERMES study. ID 66 is a nested cross-sectional study; ID 67 is a cross-sectional analysis from a cohort study and ID 68 is a cohort study. ID 67 was selected to estimate the MetS prevalence, and ID 68 was chosen to estimate the MetS incidence.

^d^ Study ID 81, data during the COVID-19 pandemic period were selected to estimate the prevalence and incidence of MetS.

**Table S5 Characteristics of the studies included in the review (continued).**

| **ID** | **Duration of ART exposure**  **(years)** | | | |  | **CD4 cell count (cells/mm^3^)** | |  | **BMI of HIVpos**  **(kg/mm^2^)** | | | |
| --- | --- | --- | --- | --- | --- | --- | --- | --- | --- | --- | --- | --- |
|  | **Mean** | **SD** | **Median** | **IQR** | **Mean** | **SD** | **Median** | **IQR** | **Mean** | **SD** | **Median** | **IQR** |
| **African Region** | | | | | | | | | | | | |
| 1 | - | - | 1.93 | 1.86-1.98 | 105.3 | 68.8 | - | - | Male: mean(sd)=18.47(29);  Female: mean(sd)=19.61(3) kg/mm^2^ | | | |
| 2 | - | - | - | - | Patients on ART: mean(sd)=350.7(171.5);  Naïve patients: mean(sd)=379.2(261.9) cells/mm^3^ | | | | Patients on ART: mean(sd)=25.3(5.3);  Naïve patients: mean(sd)=24.8(6.1) kg/mm^2^ | | | |
| 3 | Duration<12 months=24.9%;  ≥12 months=75.1% | | | | CD4 count <200 cells/mm^3^ =70.6%;  ≥200 cells/mm^3^ =29.4% | | | | - | - | - | - |
| 4 | 3.55 | - | range 0.5-8 | | Patients on ART: mean(sd)=441.6(224.4);  Naïve patients: mean(sd)=493(211.8) cells/mm^3^ | | | | Patients on ART: mean(sd)=22.1(3.94);  Naïve patients: mean(sd)=22.2(3.49) kg/mm^2^ | | | |
| 5 | Patient with MetS mean(sd)=2.4(0.7); without MetS mean(sd)=1.9(0.3) years | | | | - Patients on 1st line ART mean(sd)=382(173.2);  - Patients on 2nd line ART mean(sd)=215.1(119);  - Naïve patients mean(sd)=399(212.7) cells/mm^3^ | | | | - Patients on 1st line ART mean(sd)=24.6(4.2);  - Patients on 2nd line ART mean(sd)=23(3.5);  - Naïve patients mean(sd)=23.6(3.8) kg/mm^2^ | | | |
| 6 | - | - | - | - | 233.29 | 159.16 | - | - | 24.27 | 7.56 | - | - |
| 7 | 1.08 | 0.08 | - | - | Patients with MetS mean(sd)=199.5(157.9); without MetS mean(sd)=181.5(193.9) cells/mm^3^ | | | | Patients with MetS mean(sd)=23.1(4.4); without MetS mean(sd)=20.5(4.1) kg/mm^2^ | | | |
| 8 | - | - | - | - | CD4>200 cells/uL =43.7% | | | | 24.4 | 3.3 | - | - |
| 9 | - | - | - | - | - | - | - | - | - | - | 27 | 23-32 |
| 10 | - | - | - | - | - | - | - | - | 22.8 | 0.3 | - | - |
| 11 | - | - | - | - | - | - | 392 | 240-604 | - | - | 26.3 | 22.1-32 |
| 12 | Patients with MetS mean(sd)=6.68(3.97);  without MetS mean(sd)=3.86(2.43) years | | | | - Patients on ART group:  Patients with MetS mean(sd)=482.3(164.8); without MetS mean(sd)=397.4(213.3) cells/mm^3^  - Naïve patients’ group:  Patients with MetS mean(sd)=224.1(145.4); without MetS mean(sd)=297(194.2) cells/mm^3^ | | | | - Patients on ART group:  Patients with MetS mean(sd)=28.9(7.2); without MetS mean(sd)=22.9(5.0) kg/mm^2^  - Naïve patients’ group:  Patients with MetS mean(sd)=25(6.7); without MetS mean(sd)=21.4(5.5) kg/mm^2^ | | | |
| 13 | - | - | 3.8 | 2.1-5.6 | - | - | 587 | 406-816 | - | - | - | - |
| 14 | - | - | - | - | - | - | - | - | - | - | - | - |
| 15 | 5.4 | 3.3 | - | - | Patients on ART mean(sd)=191.4(127.8);  Naïve patients mean(sd)=417.9(206.2) cells/mm^3^ | | | | Patients on ART mean(sd)=25.1(5.9)  Naïve patients mean(sd)=23.8(5.2) kg/mm^2^ | | | |
| 16 | 4.5 | 3.2 | - | - | 357.8 | 223.6 | - | - | - | - | - | - |
| 17 | Duration<6 years=60.8%;  ≥6 years= 36.9% | | | | CD4<350 cells/uL=83.2%;  351-550 cells/uL=14.2%; ≥551cells/uL=2.6% | | | | Underweight =18.7%;  Normal BMI =68.7%; Overweight/Obesity=12.7% | | | |
| 18 | - | - | 5 | 2-8 | - | - | 476 | 309-645.5 | - | - | 22.48 | 20.56–24.86 |
| 19 | Duration≥5 years=64.4% | | | | 519 | 263 | - |  | - | - | 22.8 | 16.1-34.5 |
| 20 | - | - | - | - | - | - | - | - | 22.19 | 4.48 | - | - |
| 21 | - | - | - | - | CD4≥350 cells/mm^3^ =86% | | | | BMI<16 kg/mm^2^ =8.5%; BMI 16-18.5 kg/mm^2^ =37.4%;  BMI 18.5-25 kg/mm^2^ =51.4%; BMI >25 kg/mm^2^ =2.7% | | | |
| 22 | - | - | - | - | CD4>200 cells/mm^3^ =39.2% | | | | - | - | - | - |
| 23 | - | - | - | - | - | - | - | - | BMI<18.5 kg/mm^2^=20.3%; BMI 18.5-24.9 kg/mm^2^ =63.1%; BMI 25-29.5 kg/mm^2^ =14.5%; BMI ≥30 kg/mm^2^=2% | | | |
| 24 | - | - | 8 | 4-10 | - | - | 512 | 364-666 | - | - | - | - |
| 25 | - | - | 9 | 5-12 | - | - | 475 | 301-697 | Patients with MetS: median(iqr)=23.1(20.4-25.9);  without MetS: median(iqr)=22.0(19.4-25.2) kg/mm^2^ | | | |
| 26 | 4.3 | 2.2 | - | - | - | - | 803 | 558-1030 | - | - | 27.3 | 23.2-33.1 |
| 27 | 9.81 | 4.67 | - | - | 450 | 257 | - | - | 23.6 | 4.18 | - | - |
| 28 | - | - | - | - | Patients on ART mean(sd)=434.79(243.03);  Naïve patients mean(sd)=409.47(262.37) cells/mm^3^ | | | | Patients on ART mean(sd)=24.77(4.05);  Naïve patients mean(sd)=25.22(5.11) | | | |
| 29 | 10.07 | 3.9 | - | - | 579 | 351 | - | - | 24.3 | 4.5 | - | - |
| 30 | 8.5 | 6.3 | - | - | - | - | - | - | 26.2 | 5.3 | - | - |
| 31 | - | - | - | - | - | - | - | - | BMI<18.5 kg/mm^2^=8.1%;  BMI 18.5-24.9 kg/mm^2^ =48.9%;  BMI ≥25 kg/mm^2^ =43% | | | |
| 32 | - | - | 12 | 7-15 | - | - | 549 | 403-737 | - | - | - | - |
| 33 | 9.97 | 3.63 | - | - | - | - | 460 | 326.5-633.5 | 24.6 | 5.02 | - | - |
| 34 | - | - | - | - | - | - | 318 | 163-550 | - | - | 22.2 | 19.7-25.6 |
| **Region of the Americas** | | | | | | | | | | | | |
| 35 | - | - | - | - | - | - | 390 | 271-508 | - | - | 25.4 | 21.9–28.9 |
| 36 | 2.32 | 1.76 | - | - | - | - | - | - | 24.85 | 5.2 | - | - |
| 37 | - | - | - | - | Patients on ART:  CD4 count <200 cells/mm^3^ =20%  Naïve patients:  CD4<200 cells/mm^3^ =21% | | | | - | - | - | - |
| 38 | - | - | - | - | - | - | - | - | 27.3 | 0 | - | - |
| 39 | - | - | - | - | 382 | 220 | - | - | 25.05 | 4.6 | - | - |
| 40 | - | - | - | - | 474 | - | - | - | 28 | 7.3 | - | - |
| 41 | - | - | - | - | - | - | 513 | - | - | - | - | - |
| 42 | - | - | 4.18 | 3.47 | 525.34 | 270.39 | - | - | - | - | - | - |
| 43 | - | - | - | - | CD4 range 4-1740 cells/mm^3^ | | | | - | - | - | - |
| 44 | - | - | - | - | 473 | 322 | - | - | - | - | - | - |
| 45 | - | - | - | - | - | - | - | - | 23.8 | 3.9 | - | - |
| 46 | - | - | - | - | CD4>350 cells/mm^3^ 61.2% | | | | 24.9 | 4.4 | - | - |
| 47 | - | - | - | - | - | - | - | - | - | - | - | - |
| 48 | - | - | - | - | - | - | - | - | Patients with MetS mean(sd)=31.8(7); without MetS mean(sd)=31(8.2) kg/mm^2^ | | | |
| 49 | - | - | - | - | 264.93 | 152.12 | - | - | 23.9 | 4.3 | - | - |
| 50 | - | - | 3.8 | 2.1-5.6 | - | - | 587 | 406-816 | - | - | - | - |
| 51 | - | - | 6 | 3-14 | - | - | - | - | - | - | - | - |
| 52 | - | - | - | - | CD4≥500 cells/mm^3^ 51% | | | | - | - | - | - |
| 53 | - | - | - | - | - | - | 629 | 422-853 | 27.7 | 5.41 | - | - |
| 54 | - | - | 5.5 | 2.75-9.08 | - | - | - | - | 25.2 | 4.6 | - | - |
| 55 | 11 | 7 | 10 | 5-16 | CD4≥500 cells/mm^3^ 69% | | | | 27.5 | 5.7 | 26.5 | 23.7-30.1 |
| **European Region** | | | | | | | | | | | | |
| 56 | - | - | - | - | - | - | 479 | 302-688 | 23.4 | 3.9 | - | - |
| 57 | - | - | - | - | Patients on ART mean(sd)=0.38(0.23) ×10^9^//l  Naïve patients  Mean(sd)=0.38(0.18) ×10^9^//l | | | | 22.9 | 3 | - | - |
| 58 | - | - | - | - | - | - | - | - | 23.2 | 3.5 | - | - |
| 59 | - | - | - | - | - | - | - | - | 23.6 | 3.3 | - | - |
| 60 | - | - | - | - | - | - | 186  x 10^6^/L | 61-259 | - | - | 23.1 | 20.4–25.06 |
| 61 | - | - | - | - | 465 | 286 | - | - | 22 | 3.1 | - | - |
| 62 | - | - | - | - | Patients with MetS mean(sd)=661(76); without MetS mean(sd)=501(24) cells/mm^3^ | | | | Patients with MetS mean(sd)=26(3.3); without MetS mean(sd)=22.5(0.34) kg/mm^2^ | | | |
| 63 | - | - | - | - | Patients with Mets median(iqr)=500(342-701); without MetS median(iqr)=502(357-681) cells/mm^3^ | | | | Patient with Mets mean(sd)=25.17(4.4); without ART mean(sd)=22.8(3.38) kg/mm^2^ | | | |
| 64 | - | - | - | - | 527 | 274 | - | - | - | - | - | - |
| 65 | - | - | - | - | Patients with MetS median=190;  without MetS median=200 cells/mm^3^ | | | |  |  |  |  |
| 66 | - | - | - | - | 412 | 31 | - |  | 23.4 | 0.4 | - | - |
| 67 | - | - | - | - | CD4<200 cells/mm^3^ =19.5%; 200-500 cells/mm^3^ =51.7%; >500 cells/mm^3^ =28.8% | | | | - | - | - | - |
| 68 | - | - | - | - | CD4 at enrollment: <200 cells/mm^3^ =18.1%; 200-500 cells/mm^3^ =49.5%; >500 cells/mm^3^ =32.4%  CD4 at last visit: <200 cells/mm^3^ =5.4%; 200-500 cells/mm^3^ =44.4%; >500 cells/mm^3^ =50.3% | | | | 23.2 | 3.1 | - | - |
| 69 | - | - | 5.2 | 2.1-7.9 | - | - | 494 | 361.5-676 | - | - | 23.7 | 21.6-25.8 |
| 70 | - | - | - | - | CD4>300 cells/mm^3^ =73% | | | | 25.3 | 4.8 | - | - |
| 71 | - | - | - | - | Patients with MetS mean(sd)=443(260); without MetS mean(sd)=437(222) cells/mm^3^ | | | | Patients with MetS mean(sd)=28.3(5.6); without MetS mean(sd)=23.6(3.8) kg/mm^2^ | | | |
| 72 | - | - | - | - | - | - | 590 | 436-768 | 25.8 | 5.1 | - | - |
| 73 | 4.3 | 1.9 | - | - | 437 | 231 | - | - | 24.1 | 6.4 | - | - |
| 74 | Male group: median(iqr)=18.8(12.4-22.1);  Female group: median(iqr)=18.9(12.1-21.8) years | | | | Male: median(iqr)=559(372-755); Female: median(iqr)==552(399-704) cells/mm^3^ | | | | - | - | - | - |
| 75 | - | - | 7 | 1-18 | Patients with MetS and CD4>350 cells/mm^3^ =57%;  Patients without MetS and CD4>350 cells/mm^3^=63% | | | | - | - | - | - |
| 76 | - | - | - | - | - | - | 484 | 342-656 | - | - | - | - |
| 77 | Case group: median(iqr)=8.6(3.9-8.0);  Control group: median(iqr)=4.1(2.0-10.5) years | | | | Case group: median(iqr)=655 (510–840); Control group: median(iqr)=570 (460–790) cells/mm^3^ | | | | - | - | - | - |
| 78 | NRTI group: median(iqr)=13.21(7.31-17.52);  NNRTI group: median(iqr)=5.08(2.08-9.58);  PI group: median(iqr)=8.13(4-12.9);  INSI group: median(iqr)=3.25(1.42-5.71) years | | | | 730.61 | 330.52 | - | - | 24.15 | 3.78 | - | - |
| 79 | - | - | - | - | SiMOne study median(iqr)=440(286-636);  HIV-HY study median(iqr)=638(470-857);  STOPSHIV study median(iqr)=640(442-830) cells/mm^3^ | | | | SiMone study mean(sd)=23.6(3.4);  HIV-HY study mean(sd)=24.5(3.9); STOPSHIV study mean(sd)=24.5(4.0) kg/mm^2^ | | | |
| 80 | - | - | - | - | Patients with MetS mean(sd)=669(21); without MetS mean(sd)=705(37) cells/mm^3^ | | | | - | - | - | - |
| 81 | - | - | 12.3 | 5.6-20.9 | - | - | 729 | 552-945 | - | - | 23.8 | 21.6-25.9 |
| **South-East Asian and Western Pacific regions** | | | | | | | | | | | | |
| 82 | - | - | - | - | - | - | - | - | - By IDF criteria:  Patient with MetS mean(sd)=26(5); without MetS mean(sd)=23(3) kg/mm^2^  - By ATP III criteria:  Patient with MetS mean(sd)=27(4); without MetS mean(sd)=23(3) kg/mm^2^ | | | |
| 83 | Patient with MetS mean(sd)=5.2(3.7); without MetS mean(sd)=3.4(3.1) years | | | | Patient with MetS mean(sd)=500.6 (265.9); without MetS mean(sd)=439.9 (224.6) cells/mm^3^ | | | | Patients with MetS mean(sd)=24.5 (3.6); without MetS mean(sd)=21.7 (2.6) kg/mm^2^ | | | |
| 84 | - | - | 1.58 | 0.9-2.75 | 431.7 | 184 | - | - | 22.6 | 2.7 | - | - |
| 85 | - | - | - | - | - | - | - | - | - | - | - | - |
| 86 | NNRTI group: median(iqr)=2.6(1.75-5.0);  PI group: median(iqr)=2(0.63-5.21) years | | | | - | - | 393.5 | 267.0–534.5 | - | - | 22 | 19.9-24.6 |
| 87 | - | - | 3 | 1.75-6 | - | - | 320 | 178-560 | 24.2 | 3.1 | - | - |
| 88 | - 1st line ART group:  Age 20-40 years group mean=5.3;  Age 40-60 years group mean=5.48 years  - 2nd line ART group:  Age 20-40 years group mean=4.28;  Age 40-60 years group mean=6.4 years | | | | -1st line ART group:  Age 20-40 years group mean(sd)=502(233);  Age 40-60 years group mean(sd)=465(165) cells/mm^3^  - 2nd line ART group:  Age 20-40 years group mean(sd)=400(144);  Age 40-60 years group mean(sd)=376(183) cells/mm^3^  - Naïve patients  Age 20-40 years group mean(sd)=624(202);  Age 40-60 years group mean(sd)=602(134) cells/mm^3^ | | | | - | - | - | - |
| 89 | Duration ≤0.5 years=3.9%;  0.58-1 years=15.9%;  1.08-2 years=15.1%;  ≥25 years=65.1% | | | | CD4>200 cells/mm^3^ =12.3% | | | | - | - | - | - |
| 90 | - | - | 7.38 | 5.5-9 | - | - | 107 | 46-200 | - | - | - | - |
| 91 | 5.95 | 3.9 | range 0.5-19 years | | 416.75 | 208.33 | - | - | 22.19 | 3.3 | BMI range 14.80-35.60 kg/mm^2^ | |
| 92 | - | - | - | - | CD4≥200 cells/mm^3^ =84.8% | | | | - | - | - | - |
| 93 | - | - | 1.7 | 0.4-4.0 | - | - | 425 | 277-577 | 22 | 3.06 | - | - |
| 94 | Duration≤3 years=36%; >3 years= 64% | | | | CD4≤500 cells/mm^3^ =80%; >500 cells/mm^3^ =20.3% | | | | - | - | - | - |
| 95 | - | - | 14.1 | 10.4-14.9 | CD4≥350 cells/mm^3^ =86% | | | | - | - | - | - |
| 96 | 5.47 | 3.86 | - | - | 437 | 261 | - | - | 22.8 | 3.89 | - | - |
| 97 | - | - | 5.6 | 3.2-8.2 | - | - | 205 | 46-360 | - | - | 22.6 | 20.3-25 |
| 98 | - | - | - | - | - | - | 472.5 | 342.0-633.8 | 22.8 | 3.8 | - | - |
| 99 | - | - | - | - | - | - | 125 | 40-228 | - | - | 20.5 | 18.4-22.9 |
| 100 | - | - | - | - | 543 | 134 | - | - | Patients with MetS mean(sd)=24.33(3.38);  without MetS mean(sd)=22.63(3.34) kg/mm^2^ | | | |
| 101 | Duration ≤2 years=8.7%;  >2 – 5 years=15.3%;  >5 years=75.9% | | | | CD4≤200 cells/mm^3^ =47.6%;  >200 cells/mm^3^=52.4% | | | | BMI<23 kg/mm^2^ =64%;  ≥23 kg/mm^2^ =36% | | | |
| **The international** | | | | | | | | | | | | |
| 102 | - | - | - | - | - | - | - | - | - | - | - | - |

**References**

1. Zannou DM, Denoeud L, Lacombe K, Amoussou-Guenou D, Bashi J, Akakpo J, et al. Incidence of lipodystrophy and metabolic disorders in patients starting non-nucleoside reverse transcriptase inhibitors in Benin. Antiviral therapy. 2009;14:371-80.

2. Awotedu K, Ekpebegh C, Longo-Mbenza B, Iputo J. Prevalence of metabolic syndrome assessed by IDF and NCEP ATP 111 criteria and determinants of insulin resistance among HIV patients in the Eastern Cape Province of South Africa. Diabetes & Metabolic Syndrome: Clinical Research & Reviews. 2010;4:210-4.

3. Berhane T, Yami A, Alemseged F, Yemane T, Hamza L, Kassim M, et al. Prevalence of lipodystrophy and metabolic syndrome among HIV positive individuals on Highly Active Anti-Retroviral treatment in Jimma, South West Ethiopia. Pan African Medical Journal. 2012;13.

4. Tesfaye DY, Kinde S, Medhin G, Megerssa YC, Tadewos A, Tadesse E, et al. Burden of metabolic syndrome among HIV-infected patients in Southern Ethiopia. Diabetes & Metabolic Syndrome: Clinical Research & Reviews. 2014;8:102-7.

5. Mbunkah HA, Meriki HD, Kukwah AT, Nfor O, Nkuo-Akenji T. Prevalence of metabolic syndrome in human immunodeficiency virus-infected patients from the South-West region of Cameroon, using the adult treatment panel III criteria. Diabetology & metabolic syndrome. 2014;6:1-7.

6. Uwanuruochi VN, Michael FS, Uwanuruochi K, Okafor C, Ofoegbu EN, Onwubere BJ, et al. Assessment of Metabolic syndrome among adult human immunodeficiency virus/acquired immunodeficiency syndrome patients in a tertiary health facility in Southeast Nigeria. Journal of HIV and Human Reproduction. 2015;3:41.

7. Longo-Mbenza B, Apalata T, Longokolo M, Mambimbi MM, Mokondjimobe E, Gombet T, et al. Association of Helicobacter pylori infection with the metabolic syndrome among HIV-infected black Africans receiving highly active antiretroviral therapy. Cardiovasc J Afr. 2015;26:52-6.

8. Guira O, Tiéno H, Diendéré AE, Sagna Y, Diallo I, Yaméogo B, et al. Features of metabolic syndrome and its associated factors during highly active antiretroviral therapy in Ouagadougou (Burkina Faso). Journal of the International Association of Providers of AIDS Care (JIAPAC). 2016;15:159-63.

9. Sobieszczyk ME, Werner L, Mlisana K, Naicker N, Feinstein A, Gray CM, et al. Metabolic syndrome after HIV acquisition in South African women. JAIDS Journal of Acquired Immune Deficiency Syndromes. 2016;73:438-45.

10. Obirikorang C, Quaye L, Osei-Yeboah J, Odame EA, Asare I. Prevalence of metabolic syndrome among HIV-infected patients in Ghana: A cross-sectional study. Nigerian medical journal: journal of the Nigeria Medical Association. 2016;57:86.

11. Nguyen KA, Peer N, De Villiers A, Mukasa B, Matsha TE, Mills EJ, et al. Metabolic syndrome in people living with human immunodeficiency virus: an assessment of the prevalence and the agreement between diagnostic criteria. International journal of endocrinology. 2017;2017.

12. Muhammad FY, Gezawa ID, Uloko A, Yakasai AM, Habib AG, Iliyasu G. Metabolic syndrome among HIV infected patients: a comparative cross sectional study in northwestern Nigeria. Diabetes & Metabolic Syndrome: Clinical Research & Reviews. 2017;11:S523-S9.

13. Labhardt ND, Müller UF, Ringera I, Ehmer J, Motlatsi MM, Pfeiffer K, et al. Metabolic syndrome in patients on first‐line antiretroviral therapy containing zidovudine or tenofovir in rural Lesotho, Southern Africa. Tropical Medicine & International Health. 2017;22:725-33.

14. Ikama M, Othende FE, Makani J, Mbolla BE, Kafata LO, Mongo-Ngamami S, et al. Global cardiovascular risk of the HIV-positive patients under antiretroviral therapy in Brazzaville. Archives of Cardiovascular Diseases Supplements. 2018;10:131.

15. Osoti A, Temu TM, Kirui N, Ngetich EK, Kamano JH, Page S, et al. Metabolic syndrome among antiretroviral therapy-naive versus experienced HIV-infected patients without preexisting cardiometabolic disorders in Western Kenya. AIDS Patient Care and STDs. 2018;32:215-22.

16. Kiama CN, Wamicwe JN, Oyugi EO, Obonyo MO, Mungai JG, Roka ZG, et al. Prevalence and factors associated with metabolic syndrome in an urban population of adults living with HIV in Nairobi, Kenya. Pan African Medical Journal. 2018;29:1-9.

17. Bosho DD, Dube L, Mega TA, Adare DA, Tesfaye MG, Eshetie TC. Prevalence and predictors of metabolic syndrome among people living with human immunodeficiency virus (PLWHIV). Diabetology & metabolic syndrome. 2018;10:1-9.

18. Katoto PD, Thienemann F, Bulabula AN, Esterhuizen TM, Murhula AB, Lunjwire PP, et al. Prevalence and risk factors of metabolic syndrome in HIV‐infected adults at three urban clinics in a post‐conflict setting, eastern Democratic Republic of the Congo. Tropical Medicine & International Health. 2018;23:795-805.

19. Phalane E, Fourie CM, Schutte AE. The metabolic syndrome and renal function in an African cohort infected with human immunodeficiency virus. Southern African Journal of HIV Medicine. 2018;19.

20. Bune GT, Yalew AW, Kumie A. The global magnitude of metabolic syndrome among antiretroviral therapy (ART) exposed and ART-naïve adult HIV-infected patients in gedio-zone, southern Ethiopia: Comparative cross-sectional study, using the Adult Treatment Panel III criteria. Diabetes & Metabolic Syndrome: Clinical Research & Reviews. 2019;13:2833-41.

21. Bune GT, Yalew AW, Kumie A. Predictors of metabolic syndrome among people living with HIV in Gedeo-Zone, Southern-Ethiopia: a case–control study. HIV/AIDS-Research and Palliative Care. 2020:535-49.

22. Møller SP, Amare H, Christensen DL, Yilma D, Abdissa A, Friis H, et al. HIV and metabolic syndrome in an Ethiopian population. Annals of Human Biology. 2020;47:457-64.

23. Gebrie A. The burden of metabolic syndrome in patients living with HIV/AIDS receiving care at referral hospitals of Northwest Ethiopia: A hospital-based cross-sectional study, 2019. Diabetes & Metabolic Syndrome: Clinical Research & Reviews. 2020;14:1551-6.

24. Masyuko SJ, Page ST, Kinuthia J, Osoti AO, Polyak SJ, Otieno FC, et al. Metabolic syndrome and 10-year cardiovascular risk among HIV-positive and HIV-negative adults: A cross-sectional study. Medicine. 2020;99.

25. Hamooya BM, Mulenga LB, Masenga SK, Fwemba I, Chirwa L, Siwingwa M, et al. Metabolic syndrome in Zambian adults with human immunodeficiency virus on antiretroviral therapy: Prevalence and associated factors. Medicine. 2021;100.

26. Hanley S, Moodley D, Naidoo M. Obesity in young South African women living with HIV: A cross-sectional analysis of risk factors for cardiovascular disease. PLoS One. 2021;16:e0255652.

27. Woldu M, Minzi O, Shibeshi W, Shewaamare A, Engidawork E. Biomarkers and prevalence of cardiometabolic syndrome among people living with HIV/AIDS, Addis Ababa, Ethiopia: a hospital-based study. Clinical Medicine Insights: Endocrinology and Diabetes. 2022;15:11795514221078029.

28. Ojong E, Iya B, Djeufouata J, Ndeh F, Nsonwu A, Njongang V, et al. Metabolic syndrome and its components among HIV/AIDS patients on Antiretroviral Therapy and ART-Naïve Patients at the University of Calabar Teaching Hospital, Calabar, Nigeria. African Health Sciences. 2022;22:410-7.

29. Woldeyes E, Fisseha H, Mulatu HA, Ephrem A, Benti H, Alem MW, et al. Prevalence of Clinical Cardiovascular Disease Risk factors among HIV infected patients on anti-retroviral treatment in a Tertiary Hospital in Ethiopia. HIV/AIDS-Research and Palliative Care. 2022:297-309.

30. Kiyimba T, Kigozi F, Yiga P, Mukasa B, Ogwok P, Van der Schueren B, et al. The cardiometabolic profile and related dietary intake of Ugandans living with HIV and AIDS. Frontiers in Nutrition. 2022;9:976744.

31. Malindisa E, Balandya E, Njelekela M, Kidenya BR, Francis F, Mmbaga BT, et al. Metabolic syndrome among people living with HIV on antiretroviral therapy in Mwanza, Tanzania. BMC Endocrine Disorders. 2023;23:88.

32. Jumare J, Dakum P, Sam-Agudu N, Memiah P, Nowak R, Bada F, et al. Prevalence and characteristics of metabolic syndrome and its components among adults living with and without HIV in Nigeria: a single-center study. BMC Endocrine Disorders. 2023;23:160.

33. Abdela AA, Yifter H, Reja A, Shewaamare A, Ofotokun I, Degu WA. Prevalence and risk factors of metabolic syndrome in Ethiopia: describing an emerging outbreak in HIV clinics of the sub-Saharan Africa–a cross-sectional study. BMJ open. 2023;13.

34. Amutuhaire W, Mulindwa F, Castelnuovo B, Brusselaers N, Schwarz J-M, Edrisa M, et al., editors. Prevalence of Cardiometabolic Disease Risk Factors in People With HIV Initiating Antiretroviral Therapy at a High-Volume HIV Clinic in Kampala, Uganda. Open Forum Infectious Diseases; 2023: Oxford University Press US.

35. Johnsen S, Dolan SE, Fitch KV, Kanter JR, Hemphill LC, Connelly JM, et al. Carotid intimal medial thickness in human immunodeficiency virus-infected women: effects of protease inhibitor use, cardiac risk factors, and the metabolic syndrome. The Journal of Clinical Endocrinology & Metabolism. 2006;91:4916-24.

36. Salyer J, Lyon DE, Settle J, Elswick R, Rackley D. Coronary heart disease risks and lifestyle behaviors in persons with HIV infection. Journal of the Association of Nurses in AIDS Care. 2006;17:3-17.

37. Jacobson DL, Tang AM, Spiegelman D, Thomas AM, Skinner S, Gorbach SL, et al. Incidence of metabolic syndrome in a cohort of HIV-infected adults and prevalence relative to the US population (National Health and Nutrition Examination Survey). JAIDS Journal of Acquired Immune Deficiency Syndromes. 2006;43:458-66.

38. Mondy K, Overton ET, Grubb J, Tong S, Seyfried W, Powderly W, et al. Metabolic syndrome in HIV-infected patients from an urban, midwestern US outpatient population. Clinical Infectious Diseases. 2007;44:726-34.

39. Adeyemi O, Rezai K, Bahk M, Badri S, Thomas-Gossain N. Metabolic syndrome in older HIV-infected patients: data from the CORE50 cohort. AIDS Patient Care and STDs. 2008;22:941-5.

40. Sobieszczyk ME, Hoover DR, Anastos K, Mulligan K, Tan T, Shi Q, et al. Prevalence and predictors of metabolic syndrome among HIV-infected and HIV-uninfected women in the Women's Interagency HIV Study. JAIDS Journal of Acquired Immune Deficiency Syndromes. 2008;48:272-80.

41. Ances BM, Vaida F, Rosario D, Marquie-Beck J, Ellis RJ, Simpson DM, et al. Role of metabolic syndrome components in HIV associated sensory neuropathy. AIDS (London, England). 2009;23:2317.

42. Leite L, Sampaio A. Dietary calcium, dairy food intake and metabolic abnormalities in HIV‐infected individuals. Journal of human nutrition and dietetics. 2010;23:535-43.

43. Pullinger CR, Aouizerat BE, Gay C, Coggins T, Movsesyan I, Davis H, et al. Metabolic abnormalities and coronary heart disease risk in human immunodeficiency virus–infected adults. Metabolic syndrome and related disorders. 2010;8:279-86.

44. Ramírez-Marrero FA, De Jesús E, Santana-Bagur J, Hunter R, Frontera W, Joyner MJ. Prevalence of cardio-metabolic risk factors in Hispanics living with HIV. Ethnicity & disease. 2010;20:423.

45. de Carvalho EH, Filho DdBM, Ximenes RAdA, de Albuquerque MdFPM, de Melo HRL, Gelenske T, et al. Prevalence of hyperapolipoprotein B and associations with other cardiovascular risk factors among human immunodeficiency virus–infected patients in Pernambuco, Brazil. Metabolic Syndrome and Related Disorders. 2010;8:403-10.

46. Alencastro PR, Fuchs SC, Wolff FH, Ikeda ML, Brandão AB, Barcellos NT. Independent predictors of metabolic syndrome in HIV-infected patients. AIDS patient care and STDs. 2011;25:627-34.

47. Lauda LG, Mariath AB, Grillo LP. Metabolic syndrome and its components in HIV-infected individuals. Revista da Associacao Medica Brasileira. 2011;57:182-6.

48. Tiozzo E, Konefal J, Adwan S, Martinez LA, Villabona J, Lopez J, et al. A cross-sectional assessment of metabolic syndrome in HIV-infected people of low socio-economic status receiving antiretroviral therapy. Diabetology & metabolic syndrome. 2015;7:1-8.

49. Raposo MA, Armiliato GNdA, Guimarães NS, Caram CA, Silveira RDdS, Tupinambás U. Metabolic disorders and cardiovascular risk in people living with HIV/AIDS without the use of antiretroviral therapy. Revista da Sociedade Brasileira de Medicina Tropical. 2017;50:598-606.

50. Akl LD, Valadares AL, Moraes MJd, Pinto-Neto AM, Lagrutta B, Costa-Paiva L. Metabolic syndrome in HIV-infected middle-aged women on antiretroviral therapy: prevalence and associated factors. Brazilian Journal of Infectious Diseases. 2017;21:263-9.

51. Cibrián-Ponce A, Sánchez-Alemán MA, García-Jiménez S, Pérez-Martínez E, Bernal-Fernández G, Castañon-Mayo M, et al. Changes in cardiovascular risk and clinical outcomes in a HIV/AIDS cohort study over a 1-year period at a specialized clinic in Mexico. Therapeutics and Clinical Risk Management. 2018:1757-64.

52. Sears S, Buendia JR, Odem S, Qobadi M, Wortley P, Mgbere O, et al. Metabolic syndrome among people living with HIV receiving medical care in Southern United States: prevalence and risk factors. AIDS and Behavior. 2019;23:2916-25.

53. Yu B, Pasipanodya E, Montoya JL, Moore RC, Gianella S, McCutchan A, et al. Metabolic syndrome and neurocognitive deficits in HIV infection. Journal of acquired immune deficiency syndromes (1999). 2019;81:95.

54. Tagliari CFdS, de Oliveira CN, Vogel GM, da Silva PB, Linden R, Lazzaretti RK, et al. Investigation of SIRT1 gene variants in HIV-associated lipodystrophy and metabolic syndrome. Genetics and Molecular Biology. 2020;43.

55. Kileel EM, Lo J, Malvestutto C, Fitch KV, Zanni MV, Fichtenbaum CJ, et al., editors. Assessment of obesity and cardiometabolic status by integrase inhibitor use in REPRIEVE: a propensity-weighted analysis of a multinational primary cardiovascular prevention cohort of people with human immunodeficiency virus. Open forum infectious diseases; 2021: Oxford University Press US.

56. Jericó C, Knobel H, Montero M, Ordoñez-Llanos J, Guelar A, Gimeno JL, et al. Metabolic syndrome among HIV-infected patients: prevalence, characteristics, and related factors. Diabetes care. 2005;28:132-7.

57. Magny Bergersen B, Schumacher A, Sandvik L, Bruun JN, Birkeland K. Important differences in components of the metabolic syndrome between HIV-patients with and without highly active antiretroviral therapy and healthy controls. Scandinavian journal of infectious diseases. 2006;38:682-9.

58. Estrada V, Martínez-Larrad MT, González-Sánchez JL, de Villar NG, Zabena C, Fernández C, et al. Lipodystrophy and metabolic syndrome in HIV-infected patients treated with antiretroviral therapy. Metabolism. 2006;55:940-5.

59. Bonfanti P, Giannattasio C, Ricci E, Facchetti R, Rosella E, Franzetti M, et al. HIV and metabolic syndrome: a comparison with the general population. JAIDS Journal of Acquired Immune Deficiency Syndromes. 2007;45:426-31.

60. Palacios R, Santos J, Gonzalez M, Ruiz J, Marquez M. Incidence and prevalence of the metabolic syndrome in a cohort of naive HIV-infected patients: prospective analysis at 48 weeks of highly active antiretroviral therapy. International journal of STD & AIDS. 2007;18:184-7.

61. Badiou S, Thiébaut R, Aurillac-Lavignolle V, Dabis F, Laporte F, Cristol J-P, et al. Association of non-HDL cholesterol with subclinical atherosclerosis in HIV-positive patients. Journal of Infection. 2008;57:47-54.

62. de Saint Martin L, Pasquier E, Roudaut N, Vandhuick O, Vallet S, Bellein V, et al. Metabolic syndrome: a major risk factor for atherosclerosis in HIV-infected patients (SHIVA study). La Presse Médicale. 2008;37:579-84.

63. Squillace N, Zona S, Stentarelli C, Orlando G, Beghetto B, Nardini G, et al. Detectable HIV viral load is associated with metabolic syndrome. JAIDS Journal of Acquired Immune Deficiency Syndromes. 2009;52:459-64.

64. Guaraldi G, Ventura P, Garlassi E, Orlando G, Squillace N, Nardini G, et al. Hyperhomocysteinaemia in HIV‐infected patients: determinants of variability and correlations with predictors of cardiovascular disease. HIV medicine. 2009;10:28-34.

65. Young J, Glass TR, Bernasconi E, Rickenbach M, Furrer H, Hirschel B, et al. Hierarchical modeling gave plausible estimates of associations between metabolic syndrome and components of antiretroviral therapy. Journal of clinical epidemiology. 2009;62:632-41.

66. De Socio G, Martinelli C, Ricci E, Orofino G, Valsecchi L, Vitiello P, et al. Relations between cardiovascular risk estimates and subclinical atherosclerosis in naive HIV patients: results from the HERMES study. International journal of STD & AIDS. 2010;21.

67. Bonfanti P, De Socio GL, Marconi P, Franzetti M, Martinelli C, Vichi F, et al. Is metabolic syndrome associated to HIV infection per se? Results from the HERMES study. Current HIV research. 2010;8:165-71.

68. Bonfanti P, De Socio GV, Ricci E, Antinori A, Martinelli C, Vichi F, et al. The feature of Metabolic Syndrome in HIV naive patients is not the same of those treated: results from a prospective study. Biomedicine & Pharmacotherapy. 2012;66:348-53.

69. Hansen BR, Petersen J, Haugaard SB, Madsbad S, Obel N, Suzuki Y, et al. The prevalence of metabolic syndrome in Danish patients with HIV infection: the effect of antiretroviral therapy. HIV medicine. 2009;10:378-87.

70. Elgalib A, Aboud M, Kulasegaram R, Dimian C, Duncan A, Wierzbicki AS, et al. The assessment of metabolic syndrome in UK patients with HIV using two different definitions: CREATE 2 study. Current medical research and opinion. 2011;27:63-9.

71. Biron A, Bobin-Dubigeon C, Volteau C, Piroth L, Perré P, Leport C, et al. Metabolic syndrome in French HIV-infected patients: prevalence and predictive factors after 3 years of antiretroviral therapy. AIDS research and human retroviruses. 2012;28:1672-8.

72. Pirro M, Mannarino MR, Francisci D, Schiaroli E, Bianconi V, Bagaglia F, et al. Urinary albumin-to-creatinine ratio is associated with endothelial dysfunction in HIV-infected patients receiving antiretroviral therapy. Scientific reports. 2016;6:28741.

73. Calza L, Colangeli V, Magistrelli E, Rossi N, Rosselli Del Turco E, Bussini L, et al. Prevalence of metabolic syndrome in HIV-infected patients naive to antiretroviral therapy or receiving a first-line treatment. HIV clinical trials. 2017;18:110-7.

74. Arrive E, Viard J-P, Salanave B, Dollfus C, Matheron S, Reliquet V, et al. Metabolic risk factors in young adults infected with HIV since childhood compared with the general population. PloS one. 2018;13:e0206745.

75. Rogalska-Płońska M, Grzeszczuk A, Rogalski P, Łucejko M, Flisiak R. Metabolic syndrome in HIV infected adults in Poland. Kardiologia Polska (Polish Heart Journal). 2018;76:548-53.

76. Duro M, Manso M, Barreira S, Rebelo I, Medeiros R, Almeida C. Metabolic syndrome in human immunodeficiency virus-infected patients. International journal of STD & AIDS. 2018;29:1089-97.

77. Pommier J-D, Laouénan C, Michard F, Papot E, Urios P, Boutten A, et al. Metabolic syndrome and endocrine status in HIV-infected transwomen. AIDS. 2019;33:855-65.

78. Guaraldi G, Franconi I, Milic J, Besutti G, Pintassilgo I, Scaglioni R, et al., editors. Thymus imaging detection and size is inversely associated with metabolic syndrome and frailty in people with HIV. Open forum infectious diseases; 2019: Oxford University Press US.

79. Taramasso L, Bonfanti P, Ricci E, Maggi P, Orofino G, Squillace N, et al. Metabolic syndrome and body weight in people living with HIV infection: analysis of differences observed in three different cohort studies over a decade. HIV medicine. 2022;23:70-9.

80. Mazzitelli M, Fusco P, Brogna M, Vallone A, D’Argenio L, Beradelli G, et al. Weight of clinical and social determinants of metabolic syndrome in people living with HIV. Viruses. 2022;14:1339.

81. Borjesson RP, Galli L, Muccini C, Poli A, Clemente T, Bottanelli M, et al. Increasing incidence and prevalence of metabolic syndrome in people living with HIV during the COVID-19 pandemic. Frontiers in Medicine. 2023;10.

82. Samaras K, Wand H, Law M, Emery S, Cooper D, Carr A. Prevalence of metabolic syndrome in HIV-infected patients receiving highly active antiretroviral therapy using International Diabetes Foundation and Adult Treatment Panel III criteria: associations with insulin resistance, disturbed body fat compartmentalization, elevated C-reactive protein, and hypoadiponectinemia. Diabetes care. 2007;30:113-9.

83. Wu P-Y, Hung C-C, Liu W-C, Hsieh C-Y, Sun H-Y, Lu C-L, et al. Metabolic syndrome among HIV-infected Taiwanese patients in the era of highly active antiretroviral therapy: prevalence and associated factors. Journal of Antimicrobial Chemotherapy. 2012;67:1001-9.

84. Jeong SJ, Chin BS, Chae YT, Jin SJ, Ku NS, Baek JH, et al. Serum retinol-binding protein-4 levels are increased in HIV-infected subjects with metabolic syndrome receiving highly active antiretroviral therapy. Yonsei medical journal. 2012;53:1211-5.

85. Bajaj S, Tyagi SK, Bhargava A. Metabolic syndrome in human immunodeficiency virus positive patients. Indian journal of endocrinology and metabolism. 2013;17:117.

86. Jantarapakde J, Phanuphak N, Chaturawit C, Pengnonyang S, Mathajittiphan P, Takamtha P, et al. Prevalence of metabolic syndrome among antiretroviral-naive and antiretroviral-experienced HIV-1 infected Thai adults. AIDS patient care and STDs. 2014;28:331-40.

87. Price J, Hoy J, Ridley E, Nyulasi I, Paul E, Woolley I. Changes in the prevalence of lipodystrophy, metabolic syndrome and cardiovascular disease risk in HIV-infected men. Sexual Health. 2015;12:240-8.

88. Kolgiri V, Nagar V, Patil V. Association of metabolic syndrome and oxidative DNA damage in HIV/AIDS patients. Indian Journal of Clinical Biochemistry. 2018;33:273-81.

89. Teekawong C, Apidechkul T, Cassely M, Chansareewittaya K. Prevalence and Factors Associated with Metabolic Syndrome Among HIV/AIDS Infected Patients Who Use ARV, Nan Province, 2015-1016. Siriraj Medical Journal. 2017;69:319-29.

90. Thongpool P, Sangviroon A, Puttilerpong C. Factors Associated with Metabolic Syndrome in HIV-infected Persons. Thai Journal of Pharmacy Practice 2017;9:171-9.

91. Khaokaew P, Maneesriwongul W, Putwatana P. Selected Factors Associated with Metabolic Syndrome in Persons Living with HIV/AIDS Receiving Antiretroviral Therapy. Vajira Medical Journal: Journal of Urban Medicine. 2018;62:399-410.

92. Zhao D, Ding Y, Lin H, Chen X, Shen W, Gao M, et al. Mitochondrial haplogroups N9 and G are associated with metabolic syndrome among human immunodeficiency virus-infected patients in China. AIDS Research and Human Retroviruses. 2019;35:536-43.

93. Shi R, Chen X, Lin H, Shen W, Xu X, Zhu B, et al. Association of HIV infection with metabolic syndrome among normal or underweight young adults: evidence from the CHART cohort. BioScience Trends. 2020;14:450-6.

94. Mallya SD, Reddy T SK, Kamath A, Pandey AK, Saravu K. Determinants of metabolic syndrome and 5-year cardiovascular risk estimates among HIV-positive individuals from an Indian tertiary care hospital. AIDS Research and Treatment. 2020;2020.

95. Aurpibul L, Namwongprom S, Sudjaritruk T, Ounjaijean S. Metabolic syndrome, biochemical markers, and body composition in youth living with perinatal HIV infection on antiretroviral treatment. PloS one. 2020;15:e0230707.

96. Sashindran V, Singh AR. A study of effect of anti-retroviral therapy regimen on metabolic syndrome in people living with HIV/AIDS: Post hoc analysis from a tertiary care hospital in western India. Diabetes & Metabolic Syndrome: Clinical Research & Reviews. 2021;15:655-9.

97. Ang LW, Ng OT, Boudville IC, Leo YS, Wong CS. An observational study of the prevalence of metabolic syndrome in treatment-experienced people living with HIV in Singapore. Plos one. 2021;16:e0252320.

98. Lu W-L, Lee Y-T, Sheu G-T. Metabolic syndrome prevalence and cardiovascular risk assessment in HIV-positive men with and without antiretroviral therapy. Medicina. 2021;57:578.

99. Han WM, Law MG, Choi JY, Ditangco R, Kumarasamy N, Chaiwarith R, et al. Weight changes, metabolic syndrome and all‐cause mortality among Asian adults living with HIV. HIV medicine. 2022;23:274-86.

100. Haridas RS, Shelke SA, Deshmukh A. Study of metabolic syndrome, diabetes, and cardiovascular risk in patients with HIV at a tertiary hospital. Journal of Cardiovascular Disease Research. 2022;13:2911-7.

101. Shidhaye P, Ghate M, Gurav S, Gupte MD, Panda S. Metabolic syndrome in people living with antiretroviral therapy: A cross-sectional investigation from Pune, India. Indian Journal of Public Health. 2023;67:84-91.

102. Worm SW, Friis-Møller N, Bruyand M, Monforte ADA, Rickenbach M, Reiss P, et al. High prevalence of the metabolic syndrome in HIV-infected patients: impact of different definitions of the metabolic syndrome. Aids. 2010;24:427-35.
