# Supplementary material for: Burden of metabolic syndrome in the global adult HIV-infected population: a systematic review and meta-analysis
Source: BMC Public Health. 2024 Sep 28;24:2657. doi: 10.1186/s12889-024-20118-3 (PMC11438355; doi:10.1186/s12889-024-20118-3)
Supplement: Supplementary file 6 — Additional File 6 [file 12889_2024_20118_MOESM6_ESM.pdf]

Additional file 6

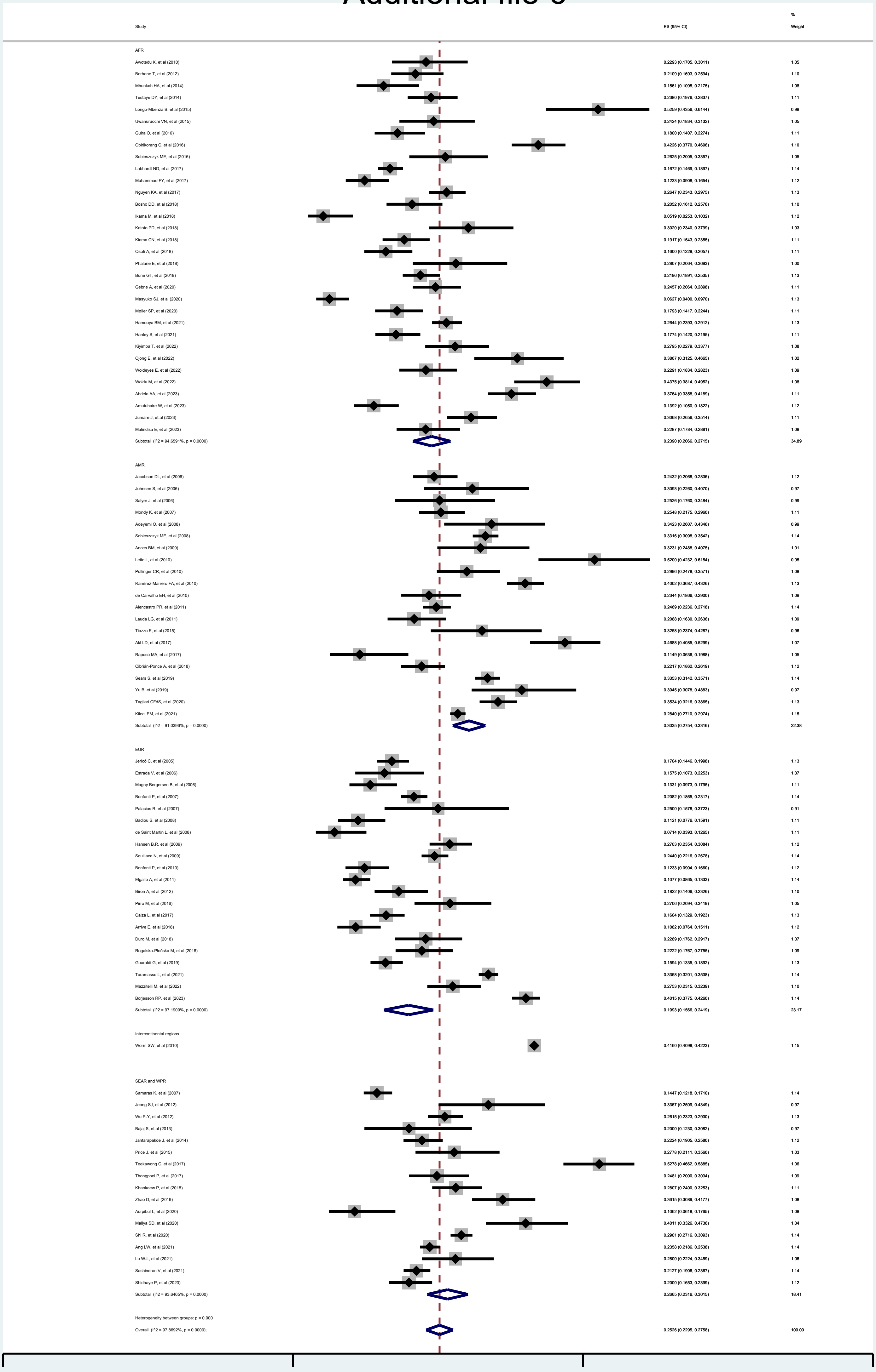

Figure S6.1 Forest plot of MetS prevalence by WHO regions for overall HIV-infected patients.

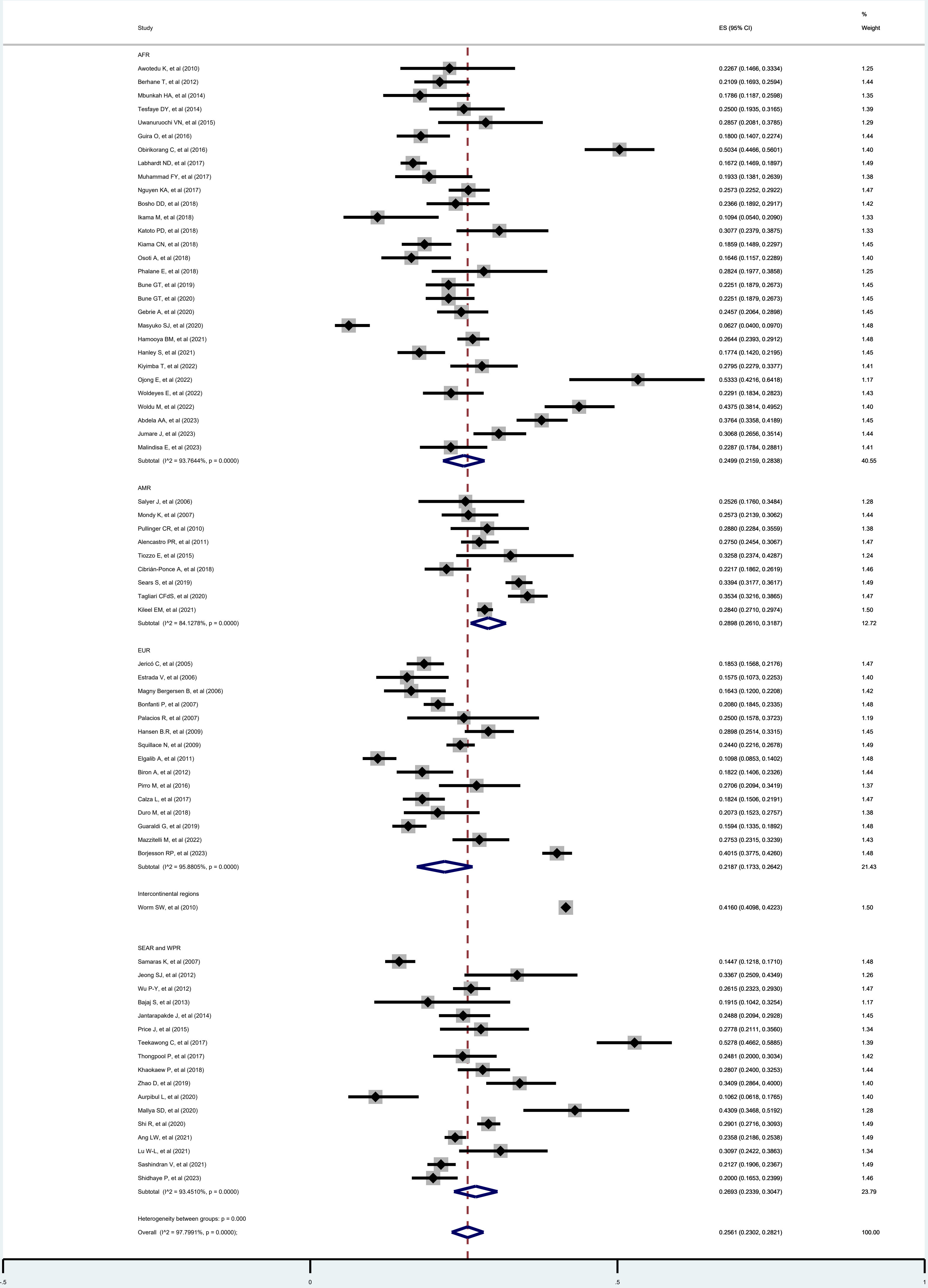

Figure S6.2 Forest plot of MetS prevalence by WHO regions for HIV-infected patients treated with AR T

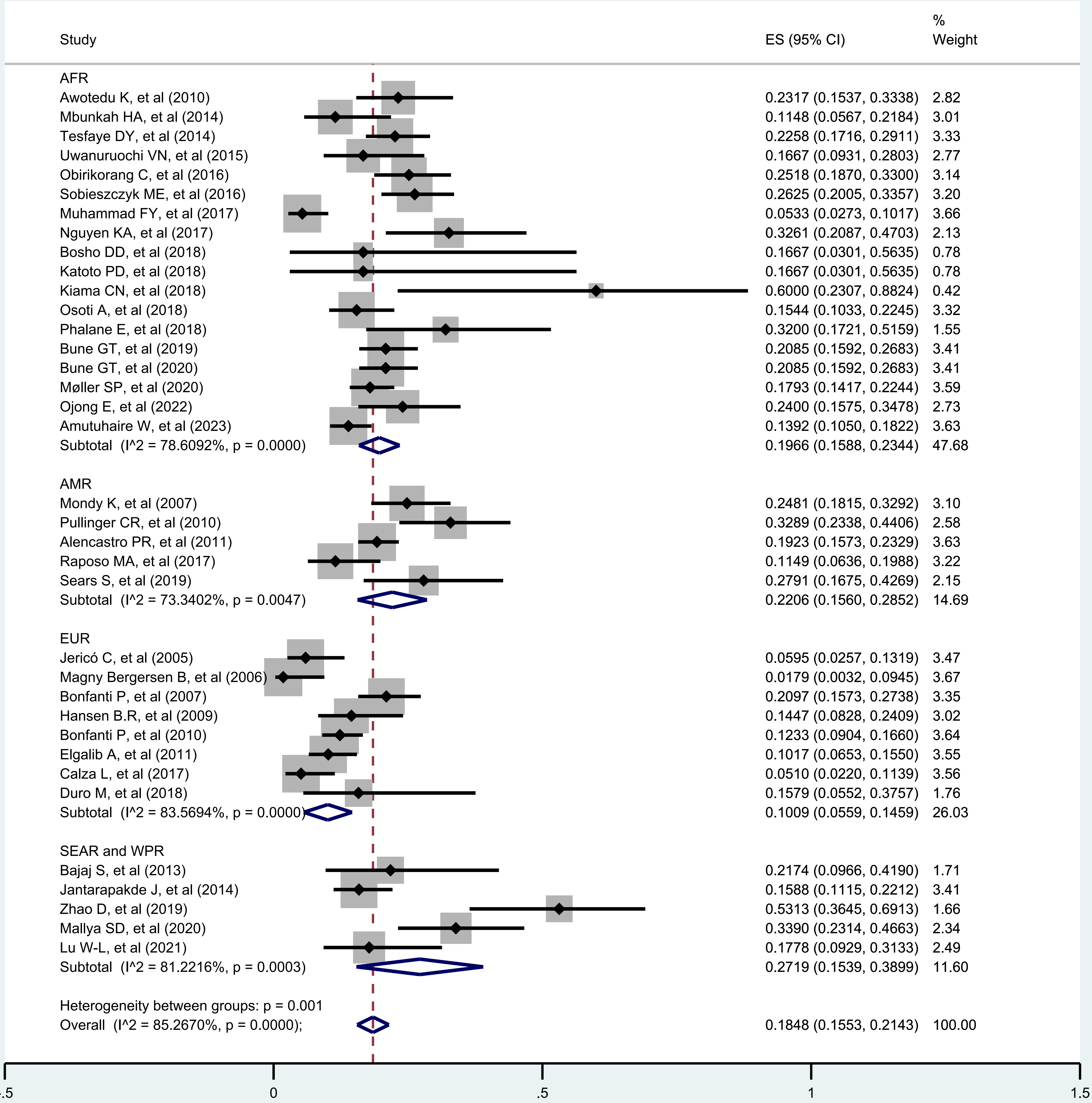

Figure S6.3 Forest plot of MetS prevalence by WHO regions for untreated patients.
