# Supplementary material for: Burden of metabolic syndrome in the global adult HIV-infected population: a systematic review and meta-analysis
Source: BMC Public Health. 2024 Sep 28;24:2657. doi: 10.1186/s12889-024-20118-3 (PMC11438355; doi:10.1186/s12889-024-20118-3)
Supplement: Supplementary file 7 — Additional File 7 [file 12889_2024_20118_MOESM7_ESM.docx]

**Additional file 7**

**Sensitivity analyses were divided by WHO regions, criteria of Metabolic syndrome, age groups of HIV-infected patients and quality assessment**.

**Table S7.1 Meta-analysis by WHO regions.**

**S7.1.1) African Region**

| **Burden of disease** | **Pooled Prevalence % (95% CI)** | **Method** |
| --- | --- | --- |
| Overall HIV-infected patients | 23.90(20.66-27.15) | Random effect, I^2^=94.7%, n=32 |
| ART-treated patients | 24.99(21.59-28.38) | Random effect, I^2^=93.8%, n=29 |
| Untreated patients | 19.66(15.88-23.44) | Random effect,  I^2^=78.6%, n=18 |

^†^ I-squared (I^2^) = variation in effect size attributable to heterogeneity

**S7.1.2) Region of the Americas**

| **Burden of disease** | **Pooled Prevalence % (95% CI)** | **Method** |
| --- | --- | --- |
| Overall HIV-infected patients | 30.35(27.54-33.16) | Random effect,  I^2^= 91%, n=21 |
| ART-treated patients | 28.98(26.10-31.87) | Random effect,  I^2^= 84.1%, n=9 |
| Untreated patients | 22.06(15.60-28.52) | Random effect,  I^2^= 73.3%, n=5 |

^†^ I-squared (I^2^) = variation in effect size attributable to heterogeneity

**S7.1.3) European Region**

| **Burden of disease** | **Pooled Prevalence % (95% CI)** | **Method** |
| --- | --- | --- |
| Overall HIV-infected patients | 19.93(15.66-24.19) | Random effect,  I^2^=97.2%, n=21 |
| ART-treated patients | 21.87(17.33-26.42) | Random effect, I^2^=95.9%, n=15 |
| Untreated patients | 10.09(5.59-14.59) | Random effect, I^2^=83.6=%, n=8 |

^†^ I-squared (I^2^) = variation in effect size attributable to heterogeneity

**S7.1.4) South-East Asian and Western Pacific Regions**

| **Burden of disease** | **Pooled Prevalence % (95% CI)** | **Method** |
| --- | --- | --- |
| Overall HIV-infected patients | 26.65(23.16-30.15) | Random effect,  I^2^= 93.6%, n=17 |
| ART-treated patients | 26.93(23.39-30.47) | Random effect,  I^2^= 93.5%, n=17 |
| Untreated patients | 27.19(15.39-38.99) | Random effect,  I^2^=73.3%, n=5 |

^†^ I-squared (I^2^) = variation in effect size attributable to heterogeneity

**Table S7.2 Meta-analysis by criteria of Metabolic syndrome**.

**S7.2.1) National Cholesterol Education Program Adult Treatment Panel (NCEP ATP) III**

| **Burden of disease** | **Pooled Prevalence % (95% CI)** | **Method** |
| --- | --- | --- |
| Overall HIV-infected patients | 25.29(22.42-28.15) | Random effect, I^2^=98%, n=64 |
| ART-treated patients | 23.11(19.60-26.62) | Random effect, I^2^=98.5%, n=49 |
| Untreated patients | 16.44(13.11-19.78) | Random effect, I^2^=85%, n=27 |

^†^ I-squared (I^2^) = variation in effect size attributable to heterogeneity

**S7.2.2) International Diabetes Federation (IDF)**

| **Burden of disease** | **Pooled Prevalence % (95% CI)** | **Method** |
| --- | --- | --- |
| Overall HIV-infected patients | 25.23(21.30-29.16) | Random effect,  I^2^=97.1%, n=30 |
| ART-treated patients | 25.80(21.33-30.27) | Random effect,  I^2^=97.2%, n=24 |
| Untreated patients | 21.17(15.53-26.81) | Random effect,  I^2^=83.2%, n=14 |

^†^ I-squared (I^2^) = variation in effect size attributable to heterogeneity

**S7.2.3) Other Definitions^‡^**

| **Burden of disease** | **Pooled Prevalence % (95% CI)** | **Method** |
| --- | --- | --- |
| Overall HIV-infected patients | 21.57(17.60-25.53) | Random effect,  I^2^=94.6%, n=14 |
| ART-treated patients | 23.20(20.20-26.21) | Random effect,  I^2^=79.7%, n=9 |
| Untreated patients | 17.05(14.19-19.92) | Random effect,  I^2^=37.6%, n=6 |

^†^ I-squared (I^2^) = variation in effect size attributable to heterogeneity

^‡^ WHO criteria; the American Heart Association (AHA) and the National Heart, Lung, and Blood Institute (NHLBI); the Harmonized Joint Scientific Statement (HJSS); the revised National Cholesterol Educational Program (NCEP) from the American Heart Association/National Heart, Lung, and Blood Institute; the Harmonization criterion for the Asian population; and unspecific criteria.

**Table S7.3 Meta-analysis by age groups of HIV-infected patients.**

**S7.3.1)** **Age >41 years**

| **Burden of disease** | **Pooled Prevalence % (95% CI)** | **Method** |
| --- | --- | --- |
| Overall HIV-infected patients | 26.78(23.96-29.59) | Random effect,  I^2^=96.7, n=47 |
| ART-treated patients | 25.29(22.44-28.14) | Random effect,  I^2^=95.7%, n=37 |
| Untreated patients | 14.69(8.85-20.54) | Random effect,  I^2^=86.2%, n=11 |

^†^ I-squared (I^2^) = variation in effect size attributable to heterogeneity

**S7.3.2) Age ≤41 years**

| **Burden of disease** | **Pooled Prevalence % (95% CI)** | **Method** |
| --- | --- | --- |
| Overall HIV-infected patients | 22.86(18.71-27.01) | Random effect,  I^2^=98.5%, n=36 |
| ART-treated patients | 25.48(20.59-30.38) | Random effect,  I^2^=98.4%, n=26 |
| Untreated patients | 17.79(14.73-20.85) | Random effect,  I^2^=80.1%, n=19 |

^†^ I-squared (I^2^) = variation in effect size attributable to heterogeneity

**Table S7.4 Meta-analysis by quality of included studies.**

**S7.4.1) Good level**

| **Burden of disease** | **Pooled Prevalence % (95% CI)** | **Method** |
| --- | --- | --- |
| Overall HIV-infected patients | 24.82(22.52-27.12) | Random effect,  I^2^=96.1%, n=55 |
| ART-treated patients | 25.45(23.07-27.83) | Random effect,  I^2^=95.1%, n=46 |
| Untreated patients | 17.24(13.85-20.63) | Random effect,  I^2^=86.8%, n=26 |

^†^ I-squared (I^2^) = variation in effect size attributable to heterogeneity

**S7.4.2) Fair level**

| **Burden of disease** | **Pooled Prevalence % (95% CI)** | **Method** |
| --- | --- | --- |
| Overall HIV-infected patients | 26.09(21.42-30.76) | Random effect,  I^2^= 98.5%, n=34 |
| ART-treated patients | 25.98(20.34-31.63) | Random effect,  I^2^=98.6%, n=22 |
| Untreated patients | 21.58(16.52-26.64) | Random effect,  I^2^=67.1%, n=10 |

^†^ I-squared (I^2^) = variation in effect size attributable to heterogeneity

**S7.4.3) Poor level**

| **Burden of disease** | **Pooled Prevalence % (95% CI)** | **Method** |
| --- | --- | --- |
| Overall HIV-infected patients | 22.88(19.56-26.20) | Fixed effect,  I^2^<25%, n=3 |
| ART-treated patients | 22.88(19.56-26.20) | Fixed effect,  I^2^<25%, n=3 |
| Untreated patients | n/a | |

^†^ I-squared (I^2^) = variation in effect size attributable to heterogeneity
