# Supplementary material for: Burden of metabolic syndrome in the global adult HIV-infected population: a systematic review and meta-analysis
Source: BMC Public Health. 2024 Sep 28;24:2657. doi: 10.1186/s12889-024-20118-3 (PMC11438355; doi:10.1186/s12889-024-20118-3)
Supplement: Supplementary file 9 — Additional File 9 [file 12889_2024_20118_MOESM9_ESM.docx]

**Additional file 9**

**Sensitivity analyses were divided by WHO regions, age groups of HIV-infected patients and quality assessment**.

**Table S9.1 Meta-analysis by WHO regions.**

**S9.1.1) African Region**

| **Common effect size** | **Pooled OR**  **(95% CI), p-value^†^** | **Method** |
| --- | --- | --- |
| HIV-infected patients vs. uninfected people | 1.428(0.776-2.628),  p=0.252 | Random effect,  I^2^= 84.6%, n=7 |
| ART-treated vs. Untreated patients | 1.417(1.029-1.952),  p=0.033 | Random effect,  I^2^= 67.3%, n=15 |

^†^ I-squared (I^2^) = variation in effect size and OR attributable to heterogeneity

P-value of Test OR=1

**S9.1.2) Region of the Americas**

| **Common effect size** | **Pooled OR**  **(95% CI), p-value^†^** | **Method** |
| --- | --- | --- |
| HIV-infected patients vs. uninfected people | 1.637(0.882-3.038),  p=0.118 | Random effect,  I^2^=93.9 %, n=5 |
| ART-treated vs. Untreated patients | 1.225(0.900-1.667),  p=0.197 | Random effect,  I^2^=41.6%, n=4 |

^†^ I-squared (I^2^) = variation in effect size and OR attributable to heterogeneity

P-value of Test OR=1

**S9.1.3) European Region**

| **Common effect size** | **Pooled OR (95% CI)**  **p-value^†^** | **Method** |
| --- | --- | --- |
| HIV-infected patients vs. uninfected people | 1.733(0.978-3.068),  p=0.059 | Random effect,  I^2^= 74.9%, n=4 |
| ART-treated vs. Untreated patients | 2.027(1.174-3.499),  p=0.01 | Random effect,  I^2^= 70.6%, n=7 |

^†^ I-squared (I^2^) = variation in effect size and OR attributable to heterogeneity

P-value of Test OR=1

**S9.1.4) South-East Asian and Western Pacific Regions**

| **Common effect size** | **Pooled OR**  **(95% CI), p-value^†^** | **Method** |
| --- | --- | --- |
| HIV-infected patients vs. uninfected people | 1.906(0.248-14.638),  p=0.535 | Random effect,  I^2^= 99.1%, n=2 |
| ART-treated vs. Untreated patients | 1.556(0.788-3.074),  p=0.2 | Random effect,  I^2^= 83.4 %, n=6 |

^†^ I-squared (I^2^) = variation in effect size and OR attributable to heterogeneity

P-value of Test OR=1

**Table S9.2 Meta-analysis by age groups of HIV-infected patients.**

**S9.2.1)** **Age >41 years**

| **Common effect size** | **Pooled OR**  **(95% CI), p-value^†^** | **Method** |
| --- | --- | --- |
| HIV-infected patients vs. uninfected people | 1.111(0.716-1.726),  p=0.638 | Random effect,  I^2^=95.3%, n=9 |
| ART-treated vs. Untreated patients | 1.930(1.219-3.058),  p=0.005 | Random effect,  I^2^= 76.1%, n=12 |

^†^ I-squared (I^2^) = variation in effect size and OR attributable to heterogeneity

P-value of Test OR=1

**S9.2.2) Age ≤41 years**

| **Common effect size** | **Pooled OR**  **(95% CI), p-value^†^** | **Method** |
| --- | --- | --- |
| HIV-infected patients vs. uninfected people | 2.513(1.535-4.116),  p<0.001 | Random effect,  I^2^=84.1%, n=8 |
| ART-treated vs. Untreated patients | 1.550(1.200-2.003),  p=0.001 | Random effect,  I^2^= 64.2%, n=14 |

^†^ I-squared (I^2^) = variation in effect size and OR attributable to heterogeneity

P-value of Test OR=1

**Table S9.3 Meta-analysis by quality of included studies.**

**S9.3.1) Good level**

| **Common effect size** | **Pooled OR**  **(95% CI), p-value^†^** | **Method** |
| --- | --- | --- |
| HIV-infected patients vs. uninfected people | 1.248(0.920-1.692),  p=0.154 | Random effect,  I^2^= 92%, n=14 |
| ART-treated vs. Untreated patients | 1.441(1.136-1.828),  p=0.003 | Random effect,  I^2^= 63.1%, n=22 |

^†^ I-squared (I^2^) = variation in effect size and OR attributable to heterogeneity

P-value of Test OR=1

**S9.3.2) Fair level**

| **Common effect size** | **Pooled OR**  **(95% CI), p-value^†^** | **Method** |
| --- | --- | --- |
| HIV-infected patients vs. uninfected people | 3.716(1.416-9.750),  p=0.008 | Random effect,  I^2^= 66.2%, n=3 |
| ART-treated vs. Untreated patients | 1.389(0.962-2.006),  p=0.08 | Random effect,  I^2^= 64.6%, n=9 |

^†^ I-squared (I^2^) = variation in effect size and OR attributable to heterogeneity

P-value of Test OR=1

**S9.3.3) Poor level**

| **Common effect size** | **Pooled OR**  **(95% CI), p-value^†^** | **Method** |
| --- | --- | --- |
| HIV-infected patients vs. uninfected people | n/a | |
| ART-treated vs. Untreated patients | n/a | |

^†^ I-squared (I^2^) = variation in effect size and OR attributable to heterogeneity

P-value of Test OR=1

**Table S9.4 Assessing the source of heterogeneity for the pooled odds ratio of HIV infection using Meta-regression.**

|  | **Coefficient** | **SE** | **95% CI** | **P-value** | **Tau^2^** |
| --- | --- | --- | --- | --- | --- |
| **Model without covariate** | 0.502 | 0.214 | 0.051 to 0.954 | 0.031 | 0.677 |
| **Study design** |  |  |  |  | 0.578* |
| Cross-sectional study (ref) |  |  |  |  |  |
| Cohort study | -0.957 | 0.836 | -2.739 to 0.824 | 0.27 |  |
| Case-control study | 1.212 | 0.851 | -0.60 to 3.026 | 0.175 |  |
| **Year of Publication** |  |  |  |  | 0.73 |
| 2000-2014 (ref) |  |  |  |  |  |
| 2015-2023 | 0.024 | 0.442 | -0.913 to 0.96 | 0.958 |  |
| **Duration of study** |  |  |  |  | 0.731 |
| ≤1 year (ref) |  |  |  |  |  |
| >1 year | -0.01 | 0.443 | -0.95 to 0.93 | 0.983 |  |
| **WHO region** |  |  |  |  | 0.853 |
| AMR (ref) |  |  |  |  |  |
| AFR | -0.197 | 0.586 | -1.454 to 1.06 | 0.742 |  |
| EUR | 0.045 | 0.67 | -1.393 to 1.482 | 0.948 |  |
| SEAR, WPR | 0.062 | 0.807 | -1.669 to 1.793 | 0.94 |  |
| **Age** |  |  |  |  | 0.636* |
| Age of HIV-infected patients (>41 years) | -1.104 | 0.515 | -2.216 to 0.009 | 0.052 |  |
| Age of HIV-uninfected controls (>41 years) | 0.253 | 0.513 | -0.856 to 1.362 | 0.631 |  |
| **BMI** |  |  |  |  | 0.728 |
| BMI of HIV-infected patients (≥25 kg/m^2^) | 0.896 | 0.61 | -0.485 to 2.276 | 0.176 |  |
| BMI of HIV-uninfected controls (≥25 kg/m^2^) | -0.51 | 0.816 | -2.356 to 1.337 | 0.548 |  |
| **Gender** |  |  |  |  | 0.572* |
| Male proportion (>60%) of HIV-infected patients | 0.104 | 0.443 | -0.846 to 1.055 | 0.817 |  |
| Male proportion (>60%) of HIV-uninfected controls | -1.119 | 0.506 | -2.203 to  -0.034 | 0.044 |  |
| **Study size** |  |  |  |  | 0.549* |
| Study size of HIV-infected patients (>300) | 0.063 | 0.544 | -1.105 to 1.23 | 0.91 |  |
| Study size of HIV-uninfected controls (>200) | -0.787 | 0.543 | -1.953 to 0.378 | 0.17 |  |
| **Matching method** | -0.034 | 0.442 | -0.97 to 0.903 | 0.94 | 0.729 |
| **Handing missing value** | omitted | | | |  |
| **Multivariable analysis** | -0.117 | 0.617 | -1.425 to 1.191 | 0.852 | 0.725 |
| **Study quality** |  |  |  |  |  |
| Poor (ref) |  |  |  |  | 0.481* |
| Fair | -0.379 | 0.919 | -2.339 to 1.58 | 0.686 |  |
| Good | -1.439 | 0.787 | -3.116 to 0.238 | 0.087 |  |

From the table S9.4, study design, age groups of both HIV-infected and uninfected samples, proportion of gender, study size and study quality were found to be the source of heterogeneity. Subgroup analyses were performed according to these variables (table S9.5); however, the levels of heterogeneity remained significant (more than 25%).

**Table S9.5 Subgroup analyses for the pooled odds ratio of HIV infection.**

| **Covariates** | **Pooled OR (95% CI)**  **p-value^†^** | **Method** |
| --- | --- | --- |
| **Study design** |  |  |
| Cross-sectional study | 1.550(1.125-2.134)  p=0.007 | Random effect,  I^2^= 91.4%, n=16 |
| Cohort study | n/a | |
| Case-control study | n/a | |
| **Age of HIV-infected patients** |  |  |
| Age ≤41 years | 2.513(1.535-4.116) p<0.001 | Random effect,  I^2^=84.1%, n=8 |
| Age >41 years | 1.111(0.716-1.726) p=0.638 | Random effect,  I^2^=95.3%, n=9 |
| **Age of HIV-uninfected controls** |  |  |
| Age ≤41 years | 1.759(1.133-2.732) p=0.012 | Random effect,  I^2^=86%, n=8 |
| Age >41 years | 1.315(0.791-2.186) p=0.291 | Random effect,  I^2^=95.8%, n=8 |
| **Gender** **of HIV-infected patients** |  |  |
| Male proportion ≤60% | 1.746(1.120-2.722) p=0.014 | Random effect,  I^2^=84.1%, n=10 |
| Male proportion >60% | 1.454(0.906-2.333)  p=0.121 | Random effect,  I^2^=96%, n=8 |
| **Gender** **of HIV-uninfected controls** |  |  |
| Male proportion ≤60% | 2.063(1.372-3.103) p<0.001 | Random effect,  I^2^=92%, n=13 |
| Male proportion >60% | 0.731(0.580-0.920)  p=0.008 | Random effect,  I^2^=50.2%, n=4 |
| **Study size of HIV-infected patients** |  |  |
| Study size ≤300 | 1.927(1.027-3.616)  p=0.041 | Random effect,  I^2^= 86.4%, n=10 |
| Study size >300 | 1.152(0.796-1.667)  p=0.455 | Random effect,  I^2^=94.7%, n=7 |
| **Study size of HIV-uninfected controls** |  |  |
| Study size ≤200 | 2.299(1.182-4.472)  p=0.014 | Random effect,  I^2^=85.2%, n=9 |
| Study size >200 | 1.267(0.841-1.908)  p=0.258 | Random effect,  I^2^=96%, n=9 |
| **Study quality** |  |  |
| Good | 1.248(0.920-1.692)  p=0.154 | Random effect,  I^2^= 92%, n=14 |
| Fair | 3.716(1.416-9.750)  p=0.008 | Random effect,  I^2^= 66.2%, n=3 |
| Poor | n/a | |

^†^ I-squared (I^2^) = variation in effect size and OR attributable to heterogeneity

P-value of Test OR=1

**Table S9.6 Assessing the source of heterogeneity for the pooled odds ratio of Antiretroviral treatment using Meta-regression.**

|  | **Coefficient** | **SE** | **95% CI** | **P-value** | **Tau^2^** |
| --- | --- | --- | --- | --- | --- |
| **Model without covariate** | 0.409 | 0.118 | 0.169 to 0.650 | 0.002 | 0.256 |
| **Study design** |  |  |  |  | 0.254* |
| Cross-sectional study (ref) |  |  |  |  |  |
| Cohort study | omitted | | | |  |
| Case-control study | 0.457 | 0.429 | - 0.419 to 1.333 | 0.295 |  |
| **Year of Publication** |  |  |  |  | 0.259 |
| 2000-2014 (ref) |  |  |  |  |  |
| 2015-2023 | 0.137 | 0.573 | -0.355 to 0.629 | 0.573 |  |
| **Duration of study** |  |  |  |  | 0.27 |
| ≤1 year (ref) |  |  |  |  |  |
| >1 year | -0.09 | 0.257 | -0.616 to 0.436 | 0.729 |  |
| **WHO region** |  |  |  |  | 0.29 |
| AMR (ref) |  |  |  |  |  |
| AFR | 0.184 | 0.355 | -0.545 to 0.912 | 0.610 |  |
| EUR | 0.531 | 0.409 | -0.306 to 1.369 | 0.204 |  |
| SEAR, WPR | 0.308 | 0.41 | -0.532 to 1.148 | 0.459 |  |
| **Age (>41 years)** | 0.188 | 0.253 | -0.335 to 0.71 | 0.466 | 0.254* |
| **BMI (≥25 kg/m^2^)** | -0.259 | 0.258 | -0.798 to 0.28 | 0.327 | 0.181* |
| **Male proportion (>60%)** | 0.172 | 0.241 | -0.321 to 0.665 | 0.482 | 0.269 |
| **Study size** |  |  |  |  | 0.208* |
| Study size of treated patients (>300) | -0.136 | 0.245 | -0.637 to 0.365 | 0.583 |  |
| Study size of untreated patients (>100) | 0.026 | 0.245 | -0.476 to 0.528 | 0.916 |  |
| **Matching method** | -0.077 | 0.332 | -0.755 to 0.601 | 0.818 | 0.269 |
| **Handing missing value** | omitted | | | |  |
| **Multivariable analysis** | -0.062 | 0.266 | -0.606 to 0.481 | 0.817 | 0.268 |
| **Study quality** |  |  |  |  | 0.197* |
| Poor (ref) |  |  |  |  |  |
| Fair | -1.307 | 0.591 | -2.516 to  -0.098 | 0.035 |  |
| Good | -1.267 | 0.572 | -2.436 to  -0.098 | 0.035 |  |

From the table S9.6, study design, age groups of HIV-infected patients, BMI of HIV-infected patients, study size and study quality were found to be the source of heterogeneity. Subgroup analyses were performed according to these variables (table S9.7); however, the levels of heterogeneity remained significant (more than 25%).

**Table S9.7 Subgroup analyses for the pooled odds ratio of Antiretroviral treatment.**

| **Covariates** | **Pooled OR (95% CI)**  **p-value^†^** | **Method** |
| --- | --- | --- |
| **Study design** |  |  |
| Cross-sectional study | 1.444(1.177-1.771)  p=0.001 | Random effect,  I^2^= 63%, n=30 |
| Cohort study | n/a | |
| Case-control study | 2.356(0.522-10.635)  p=0.265 | Random effect,  I^2^= 95.1%, n=2 |
| **Age of HIV-infected patients** |  |  |
| Age ≤41 years | 1.550(1.200-2.003)  p=0.001 | Random effect,  I^2^= 64.2%, n=14 |
| Age >41 years | 1.930(1.219-3.058)  p=0.005 | Random effect,  I^2^= 76.1%, n=12 |
| **BMI of HIV-infected patients** |  |  |
| BMI <25 kg/m^2^ | 1.757(1.366-2.260)  p<0.001 | Random effect,  I^2^= 58.7%, n=15 |
| BMI ≥25 kg/m^2^ | 1.396(0.883-2.205) p=0.153 | Random effect,  I^2^=72.5%, n=7 |
| **Study size of treated patients** |  |  |
| Study size≤300 | 1.526(1.108-2.100)  p=0.01 | Random effect,  I^2^=63.7%, n=18 |
| Study size>300 | 1.329(1.046-1.689)  p=0.02 | Random effect,  I^2^=61.1%, n=13 |
| **Study size of untreated patients** |  |  |
| Study size≤100 | 1.449(1.043-2.013)  p=0.027 | Random effect,  I^2^=62.4%, n=20 |
| Study size>100 | 1.399(1.105-1.771)  p=0.005 | Random effect,  I^2^=66.1%, n=11 |
| **Study quality** |  |  |
| Good | 1.441(1.136-1.828)  p=0.003 | Random effect,  I^2^= 63.1%, n=22 |
| Fair | 1.389(0.962-2.006)  p=0.08 | Random effect,  I^2^= 64.6%, n=9 |
| Poor | n/a | |

^†^ I-squared (I^2^) = variation in effect size and OR attributable to heterogeneity

P-value of Test OR=1
