# Supplementary material for: Burden of metabolic syndrome in the global adult HIV-infected population: a systematic review and meta-analysis
Source: BMC Public Health. 2024 Sep 28;24:2657. doi: 10.1186/s12889-024-20118-3 (PMC11438355; doi:10.1186/s12889-024-20118-3)
Supplement: Supplementary file 10 — Additional File 10 [file 12889_2024_20118_MOESM10_ESM.docx]

**Additional file 10**

**Publication Bias Tests**

**1. Egger's test**

**Table S10 Egger's test.**

|  | **Number of studies** | **Slope** | | | **Bias** | | |
| --- | --- | --- | --- | --- | --- | --- | --- |
|  |  | **Coefficient** | **SE** | **P-value** | **Coefficient** | **SE** | **P-value** |
| Pooled OR of HIV-infected patients vs. uninfected people | 18 | -0.357 | 0.212 | 0.114 | 3.028 | 1.458 | 0.057 |
| Pooled OR of ART-treated vs. Untreated patients | 32 | 0.275 | 0.241 | 0.261 | 0.369 | 0.783 | 0.641 |

**2. Funnel plots**

**Figure S10.1 Funnel plot for pooled OR of HIV-infected patients vs. uninfected people.**

**Figure S10.2 Funnel plot for pooled OR of ART-treated vs. Untreated patients.**
